# From Soil to Plate: Lithium and Other Trace Metals Uptake in Vegetables Under Variable Soil Conditions

**DOI:** 10.3390/toxics13110956

**Published:** 2025-11-05

**Authors:** Nadia Paun, Ramona Zgavarogea, Violeta-Carolina Niculescu, Ana Maria Nasture, Iulian Voicea, Diana Ionela Popescu (Stegarus)

**Affiliations:** 1 National Research and Development Institute for Cryogenic and Isotopic Technologies—ICSI Ramnicu Valcea, 4th Uzinei Street, P.O. Box Raureni 7, 240050 Ramnicu Valcea, Romania; nadia.paun@icsi.ro (N.P.); ramona.zgavarogea@icsi.ro (R.Z.); ana.nasture@icsi.ro (A.M.N.); 2 National Institute of Research—Development for Machines and Installations Designed for Agriculture and Food Industry, 6th Ion Ionescu de la Brad Blvd., Sector 1, 013813 Bucharest, Romania

**Keywords:** transfer factor, food safety, lithium, trace metal, Romanian agriculture, soil–plant transfer

## Abstract

The bioaccumulation of trace metals in edible crops is a key pathway of dietary exposure, with direct implications for environmental health and food safety. This study specifically investigated the bioaccumulation and soil–plant transfer of lithium (Li) in edible crops, alongside other selected trace metals (Cu, Mn, Sr, Zn), to understand its unique environmental mobility and dietary exposure risks in onion, garlic, green salad, cucumber, and zucchini cultivated across Romania. Forty-two paired samples of vegetable tissues and rhizosphere soils were collected from eleven agricultural regions, and were analyzed using spectroscopic techniques. Soils were predominantly neutral to slightly acidic, conditions that significantly affected metal mobility and uptake. Results revealed element-specific decoupling between soil and plant concentrations. Essential micronutrients (Zn, Cu) showed higher transfer factors, consistent with active physiological uptake, while toxic non-essential metals (Pb, Cd) remained below European regulatory thresholds, reflecting effective exclusion mechanisms. Lithium exhibited spatially heterogeneous transfer patterns, strongly influenced by local geochemical variability. Curvilinear soil–plant relationships for Fe, Zn, Sr, Mn, Cu, and Li further underscored the role of soil chemistry in shaping translocation. These findings establish a robust baseline for assessing dietary risks, confirming the current low-risk status of vegetables in the surveyed regions, and provide valuable guidance for sustainable agricultural management and food safety monitoring.

## 1. Introduction

The uptake of lithium (Li) and other trace metals by vegetables has become an area of growing interest due to their implications for plant health, agricultural productivity, and food safety. Although lithium is not an essential nutrient for plants, its presence in soil can influence growth and development. Similarly, trace metals such as zinc (Zn) and copper (Cu) are essential at low concentrations, but may become toxic when present in excess. Understanding the mechanisms of uptake and accumulation of these elements is critical for assessing environmental and health risks associated with vegetable consumption.

Lithium is a naturally occurring element found in trace amounts in soil, water, plants, and in animals consuming these plants. While commonly associated with psychiatric treatment, research since the 1970s has suggested that low levels of lithium may act as a micronutrient. Animal studies have shown that lithium deficiency can significantly impair reproductive success [1,2]. In humans, lithium at low doses may confer cardiovascular, musculoskeletal, metabolic, and cognitive benefits, as well as exerting anti-inflammatory and antioxidant effects, whereas excessive intake without medical supervision can lead to neurotoxicity and renal impairment [3]. In plants, lithium content varies depending on the species, organ, and environmental conditions. Low lithium concentrations can stimulate growth, whereas higher concentrations induce toxicity [4,5]. Data on lithium content in foods remain limited but suggest variability. Nuts, legumes, grains, and vegetables are the primary dietary sources, with green leafy vegetables often containing higher concentrations, although definitive conclusions are constrained by data scarcity [6]. Lithium is readily absorbed by plants, and its uptake is influenced by soil pH, with acidic soils promoting higher accumulation compared to alkaline soils [7]. For example, Solanaceae family members, such as tomatoes and potatoes, can accumulate lithium concentrations up to 30 mg/kg in dry weight, while corn grains may contain as little as 0.05 µg/kg [6,7]. However, excessive lithium levels can impair growth and disrupt physiological processes. Although lithium is not considered essential like sodium, potassium, or calcium, it interacts with other trace elements and biomolecules, potentially modulating metabolic pathways, including those relevant to human health [7].

Trace metals in vegetables have received significant attention due to their dual roles in plant physiology and human nutrition [8]. Plants acquire essential elements such as Co, Cu, Fe, Ni, and Zn from soil, water, and air; however, they may also accumulate non-essential and potentially toxic elements such as Pb, Hg, Li, Cd, and As. Metal uptake is a highly complex process involving transfer from soil into root cells [9]. Roots enhance uptake through mechanisms including rhizosphere acidification, proton pumping, and exudation of low-molecular-weight chelators, while intracellular sequestration prevents excessive accumulation [10]. Environmental pollution from anthropogenic sources, including industrial and agricultural activities, has exacerbated trace metal accumulation in soils, increasing the risk of uptake by vegetables [11]. Essential microelements function as cofactors in enzymes and proteins involved in major metabolic pathways [12], influencing photosynthesis, respiration, transpiration, and cell membrane integrity. Conversely, non-essential metals may impair physiological processes, and their presence in edible plant tissues represents a potential health risk [10,12].

In Romania, vegetables are a central component of the diet, providing minerals, fiber, vitamins, and antioxidants [4,13]. However, they are susceptible to contamination by toxic metals due to both natural processes and anthropogenic activities [12,14]. Monitoring trace metal accumulation is therefore essential for food safety [15,16]. Trace element toxicity manifests when concentrations exceed thresholds: essential metals support metabolic processes at low levels, while non-essential metals, including Pb, Hg, Li, Cd, and As, pose risks even at minimal concentrations [16,17]. For example, Cu facilitates iron absorption, energy production, and nerve function [18], while Zn supports immune function, wound healing, and DNA synthesis [19]. Ca is vital for bones, muscles, and nerve signaling; Fe is critical for oxygen transport; Mg regulates neuromuscular function and metabolic processes; iodine is required for thyroid hormone synthesis; and Na and K regulate fluid balance and neuromuscular activity [20,21]. In contrast, non-essential trace metals and metalloids can impair neurological development, renal function, and other systems. Mercury, cadmium, arsenic, and lithium, when present in excess, are associated with systemic toxicity, carcinogenicity, and organ-specific damage [22]. Strontium (Sr) uptake in vegetables occurs primarily via root absorption through pathways like those used for calcium, reflecting their chemical similarity. Its bioavailability is influenced by soil pH, calcium levels, organic matter content, and plant species, with acidic soils and low calcium generally promoting greater uptake. Although stable strontium is not inherently toxic, excessive accumulation can disrupt calcium homeostasis, impair growth, and induce nutrient imbalances, particularly in sensitive crops such as Chinese cabbage [23,24].

The main objective of the present study was to investigate the accumulation of lithium and trace metals in various vegetables, including onion, garlic, green salad, cucumber, and zucchini, and their corresponding rhizosphere soils. The aim of environmental monitoring must now expand beyond conventional pollutants to include elements of emerging concern, with lithium being a prime example. This focus is justified by Li’s unique physiological role. Unlike other non-essential elements, Li has recognized pharmacological properties, and debate persists regarding its potential to act as a micronutrient or a cumulative toxicant at low environmental concentrations. Given its established biological activity, the dietary exposure pathway through crops becomes a crucial subject for food safety assessment. Therefore, while this study provides a comprehensive analysis of several key trace metals (Cu, Mn, Sr, and Zn), its primary objective was to specifically isolate and characterize the transfer and bioaccumulation of lithium from regionally variable soils into edible crops, a critical data gap that must be addressed in the current environmental literature. To bridge these knowledge gaps and establish the unique scientific advance of this work, this study employed a multi-tiered analytical framework that provides three critical novelties: (a) the systematic inclusion of Li alongside classical trace metals; (b) the establishment of a broad regional geochemical baseline and Transfer Factor (TF) dataset for Eastern European agroecosystems; and (c) a novel side-by-side comparison of metal uptake in field-grown versus greenhouse-grown garlic. This comprehensive approach allowed to determine which metal uptake patterns are driven by regional geogenic background versus by localized agricultural practice. Considering these emerging concerns, this comprehensive assessment, conducted within the context of sustainable agriculture and environmental health in Romania, focused on determining the concentrations and chemical species of trace metals, as well as evaluating the physical and chemical characteristics of soils. The study aimed to provide critical insights into the interactions between soil composition, trace element uptake, and potential implications for plant and human health.

## 2. Materials and Methods

### 2.1. Study Area and Sampling

Vegetables and their corresponding cultivation soils were collected from various regions across Romania during 2024. The sampled vegetables included onion, garlic, salad, cucumber, and zucchini, alongside the associated soils. These matrices were selected based on their prevalence in typical daily diets. The study assessed the metal concentrations in a total of 190 vegetable samples and 38 soil samples. The vegetable samples were collected from 19 unique locations and comprised five crop types: onion (*Allium cepa* L., *n* = 38), garlic (*Allium sativum* L., *n* = 38), green salad (lettuce, *Lactuca sativa* L., *n* = 38), cucumber (*Cucumis sativus* L., *n* = 38), and zucchini (*Cucurbita pepo* L., *n* = 38). For each of these 38-sample sets, half of the samples (*n* = 19) were collected from the field, and the other half (*n* = 19) were collected from a greenhouse setting to evaluate the influence of growing conditions on trace element uptake. The 38 soil samples were collected from the same 19 locations (Supplementary Material File S1, Figure S1) at two distinct depth intervals: the topsoil layer (0–10 cm, forward denoted “5 cm”) and the subsoil layer (10–20 cm, forward denoted “15 cm”) to evaluate vertical distribution patterns of trace elements (19 locations × 2 depths = 38 soil samples). For subsequent discussion, figures, and tables, these samples are referred to by their respective mid-depths: 5 cm and 15 cm.

### 2.2. Sample Preparation and Analytical Procedures

All the reagents, including hydrochloric acid (37%), nitric acid (65% for analysis EMSURE) were purchased and used without further purification from Merck KGaA, Darmstadt, Germany. Ultrapure water was made using an Elga Veolia PURELAB Flex3 system (Elga LabWater, High Wycombe, UK).

Since samples have a very complex composition, the strong acid extraction of the matrix is mandatory to ensure complete metal solubility. Transformation of solid pieces into homogeneous liquid phase before sample analysis is first required. Before elemental analysis, both the soil and food samples were oven-dried at 60 °C for 12 h or until a constant weight was obtained. Then, the dried samples were ground in a stainless-steel mill and passed through a 0.45 mm mesh. The internal laboratory method for digestion of samples was optimized to be 0.5 g of sample and 10 mL of 65% HNO_3_ for vegetables and 0.5 g of sample and 10 mL aqua regia (2.5 mL 65% HNO_3_ + 7.5 mL 37% HCl) for soil.

A microwave oven model MARS6 CEM One Touch (CEM Corporation, Matthews, NC, USA) was used to digest vegetables and soil samples for quantitative concentration. The digestion was performed with the controlled program (pressure and temperature) for 15 min at 200 °C. After complete digestion and cooling, the samples were filtered, transferred to 50 mL graduated polypropylene tubes, and diluted to volume with deionized water.

A representative sample of 0.5 g of soil is mineralized using the EasyPrep, iwave MARS6 CEM One Touch closed vessel system (CEM Corporation, Matthews, NC, USA) with the concentrated acid mixture 10 mL aqua regia, according to the temperature-controlled digestion program (Supplementary Material File S1, Table S1) (Microwave digestion of soil, Montana-CRM Leach from CEM Mars 6 Method Compendium Note 2019) [25].

Plant material samples (onion, garlic, lettuce and cucumber) in an amount of 0.5 g of dry sample + 10 mL of 65% HNO_3_ are digested using the same digestion system according to the digestion program (Supplementary Material File S1, Table S2) (Microwave digestion of Plant Tissue-CEM Mars 6 Method Compendium Note 2019/Plum CEM Mars 6 from Compendium Note 2019) [25].

After cooling, the samples were made up to the mark in 50 mL volumetric flasks. The samples were clear, colorless, and free of particles after dilution.

### 2.3. Analytical Procedure

Inductively coupled plasma atomic emission spectroscopy (ICP-OES Plasma Quant 9100, Analytic Jena GmbH, Jena, Germany) was used for multielement identification and quantification. To minimize instrumental multielement background, the standard sample introduction system of the ICP optic spectrometer was replaced with inert components, namely a ceramic D-torch and a ceramic injector (Glass Expansion, Melbourne, Australia), with a PFA cyclonic spray chamber and concentric nebulizer Opal Mist & Dura Mist (Glass Expansion, Melbourne, Australia). The optimized ICP-OES operating parameters are summarized in Supplementary Material File S1, Table S3. Based on the literature [26,27,28], the most sensitive emission wavelength of the elements analyzed was chosen, working close to the detection limit. The results were calculated by reference to a standard calibration in the range of 0.5 to 10 mg/L for soil matrix and 0.5–25 µg/L for vegetables matrix. Linearity was checked using the Mandel test [28,29].

The instrumental parameters were carefully adjusted to secure accuracy and reproducibility. The plasma gas and argon gas flow rates were configured at 12.0 L/min, while the flows of auxiliary and nebulizer gas were kept at 0.5 L/min and 0.7 L/min, respectively. The RF power was adjusted to 1200 W, and the purge gas flow rate was maintained at high level. A peristaltic flow of 1.1 mL/min and a wash rate of 1.5 mL/min were used, while the rinsing time was 60 s to avoid cross-contamination. The read delay was set at 30 s, the actual read time being 3 s per replicate. Measurements were conducted in triplicates. All elemental concentrations reported for the vegetable samples are expressed on a dry weight (DW) basis.

The quality assurance/quality control QA/QC of the analytical method was validated using certified reference materials and spike recoveries, confirming high accuracy (Recovery % from 91.5% to 109.6%) and precision (% RSDr typically below 5%). Detailed analytical performance parameters, including R^2^ values, limits of detection (LOD), limits of quantification (LOQ), and strategies for spectral interference correction (where necessary), are presented for both soil and vegetable matrices in Supplementary Material File S1, Table S4.

### 2.4. Data Analysis

Transfer factor (TF) allows direct comparison of metal transfer efficiency from soil to plant and it is defined as

(1) $$TF=\frac{Metal\;concentration\;in\;plant\;tissue\;(mg/kg)}{Metal\;concentration\;in\;soil\;(mg/kg)}$$

The Transfer Factor (TF) was calculated for all metals where quantifiable concentrations existed. According to the defined scope, metal concentration in soil was derived exclusively from soil samples collected at the 5 cm depth. All available data were included in calculating the regional minimum and maximum TF ranges, including those reported at the analytical Limit of Detection (LOD) for certain elements such as Cd and Li.

The Standard Error of the Mean (SE) and the Mean Transfer Factor (Mean TF) have been calculated for each metal in each vegetable, aggregated across all 19 regions:

(2) $$SE=\frac{SD}{\sqrt{n}}$$

where SD is the standard deviation of TF values across all regions, and n is the number of regions.

The statistical analysis examined the vertical distribution of trace metals in Romanian agricultural soils and investigates species, as well as morphotype-specific accumulation patterns in vegetables cultivated under both greenhouse and open-field conditions. A combination of non-parametric statistical tests and network-based co-occurrence analyses was employed to identify distinct spatial and biological patterns, which are associated with legacy industrial activities and specific cultivation environments. In this context, “legacy industrial activities” refer to the proximity of agricultural sites to areas historically impacted by mining, metallurgy, and chemical industries, which may contribute to elevated trace metal concentrations in soils. “Specific cultivation environments” refer to differences between greenhouse and open-field cultivation systems, including soil management practices, fertilizer application, and controlled irrigation. These parameters (regional industrial background, cultivation system, and soil depth) were included as explanatory variables in non-parametric statistical tests and multivariate analyses to identify spatial and environmental patterns of trace metal distribution and plant uptake. All statistical analyses were conducted using Python 3.12.3 (Python Software Foundation, Wilmington, DE, USA) [30], key libraries included pandas 2.2.3 (Seattle, DC, USA, WatchGuard Technologies) for tabular data management and preprocessing [31], numpy 1.26.4 for numerical operations and array manipulation, scipy 1.13.1 for statistical testing (including Mann–Whitney U and Shapiro–Wilk tests), statsmodels 0.14.1 for ANOVA and advanced statistical modeling, scikit-learn 1.5.2 for KMeans clustering, and plotly 5.22.0 for interactive visualization and figure generation, including boxplots and multi-panel subplots [31,32,33]. Network-based co-occurrence analyses were performed using VOSviewer version 1.6.19 (Centre for Science and Technology Studies, Leiden University, Leiden, The Netherlands), selected for its robust clustering algorithms and intuitive visualization capabilities for bibliometric and entity-relationship mapping.

## 3. Results and Discussion

### 3.1. Experimental Results

#### 3.1.1. Soil Concentrations of Metals and Metalloids

The geochemical profile of agricultural soils in Romania is characterized by a dual system: a naturally high geogenic background of certain elements, mainly in metalliferous zones, overlaid by acute, localized anthropogenic contamination [34,35].

The full dataset (Supplementary Material File S2), encompassing 19 distinct counties across Romania, revealed significant heterogeneity in soil contamination reflective of localized anthropogenic pressure versus natural background [36,37].

The metal profiles of soils reveal marked spatial heterogeneity in their concentrations, reflecting both natural geochemical backgrounds and anthropogenic influences (Table 1 and Supplementary Material File S2).

Across most counties, soils exhibited higher total concentrations of Fe, Mn, Zn, Sr, and Li compared to other trace metals, with notable enrichment in industrially influenced areas such as Ilfov, Buzau, and Dolj (Supplementary Material File S2). Vertical distribution patterns were element- and site-specific. In most regions, surface soils (0–5 cm) exhibited higher concentrations of Zn, Pb, Ni, and Cu compared to deeper soils (15 cm), reflecting recent anthropogenic inputs such as fertilizer application, traffic emissions, and industrial deposition. However, in certain locations (e.g., Bacau, Buzau, Dolj), Pb, Ni, and Cr occasionally presented higher values at 15 cm depth, which may indicate downward migration or legacy contamination from earlier deposition events. Elements including Sr, Fe, Mn, and Li displayed relatively uniform concentrations between soil depths, suggesting that these elements are primarily geogenic and less influenced by surface inputs. Cd and Hg were mostly below detection limits, except in regions such as Buzau and Bihor, indicating occasional contamination hotspots. These depth-related trends underscore the importance of the topsoil layer in determining trace metal availability for plant uptake.

The correlation of mean concentrations (provided within the Supplementary Material File S2) with the regulatory ranges (Table 1) and Natural Geochemical Background (NGB) values provides a comprehensive understanding of the regional distribution, geogenic influence, and anthropogenic inputs of metals in the investigated soils. Elements such as Fe, Mn, and Sr exhibit consistently high concentrations that align with the expected natural levels derived from the parent geological material of Romanian soils. The relatively stable vertical profiles observed for Mn, Cd, and Co can be attributed to the homogenizing effect of agricultural tillage within the 0–20 cm layer, which minimizes sharp concentration gradients between the sampled depths. Sr also reflects a pronounced geogenic control, maintaining high concentrations across both depths and exceeding typical global averages, consistent with the regional lithological background. In contrast, Li represents a clear exception, showing concentrations far above the expected regional NGB (approximately 20 mg/kg) [39,40]. This enrichment, reaching up to 22-fold relative to the background, indicates that Li accumulation is not due to localized pollution but rather to a broader geological or anthropogenic influence not fully captured by the current regulatory framework. Spatial analysis further supports this interpretation, revealing distinct hotspots in counties such as Dolj and Calarasi (Figure S2, Supplementary Material File S1), suggesting regionally variable sources of enrichment. Cr, on the other hand, is found at concentrations significantly below both the statutory Normal Value (NV) and the expected NGB, signifying that although Cr may be naturally abundant in certain Romanian geological formations, the specific study sites are comparatively depleted. The distribution patterns of Ni and Cr, with slightly higher or similar mean values in deeper layers, indicate that their presence is primarily governed by the natural composition of the parent material rather than anthropogenic surface deposition. The contaminants such as Cu and Pb display distinct evidence of surface accumulation, suggesting anthropogenic influence. Cu shows a marked depth gradient, with higher values in the surface layer compared to subsurface soil, and concentrations approaching the Alert Threshold (AT). This trend demonstrates consistent topsoil enrichment rather than isolated contamination events. Pb exhibits a similar pattern, with higher values in the surface layer exceeding the NV but remaining below the AT. The elevated Pb and Cu concentrations observed in areas such as Ilfov, Ialomița, and Calarasi indicate recent surface deposition, while the higher values in Bacau, Buzau, and Dolj likely reflect downward migration or legacy contamination.

Other elements, including Zn, Cd, As, and Hg, remain below levels of regulatory concern. Their mean concentrations fall below the NV and NGB limits, confirming negligible contamination risk at the regional scale. Despite some isolated exceedances in the range values, the overall environmental risk associated with these elements remains low.

Overall, the integrated evaluation of concentration data and regulatory thresholds highlights a dual influence of natural and anthropogenic factors. The widespread geogenic enrichment of elements such as Li and Sr contrasts with the localized surface accumulation of Cu, Pb, and Ni. The limited vertical variability across most elements reflects the mixing effect of agricultural tillage, while the spatial heterogeneity observed across counties underscores the combined impact of geological background and land-use practices on the regional metal distribution in Romanian soils.

#### 3.1.2. Vegetables Concentrations of Metals and Metalloids and Their Transfer Factors

Onion samples, collected from both open-field and greenhouse conditions, demonstrated clear cultivation-dependent bioaccumulation (Table 2 and Supplementary Material File S2).

Zn, Cu, and Mn were the most consistently accumulated metals, whereas Pb, Cd, Hg, and As were detected at trace levels, indicating limited translocation from soils to edible tissues (Supplementary Material File S2). A pronounced soil–plant decoupling was observed for Sr and Fe: despite high soil concentrations (often exceeding species-specific 5000 mg/kg Fe and 1000 mg/kg Sr), onion uptake was minimal, confirming their restricted mobility in plant tissues. In contrast, Li showed moderate accumulation in several regions (e.g., Ialomita, Olt, and Calarasi), pointing to potential bioavailability under specific geochemical settings. Regional differences highlighted the role of both soil geochemistry and cultivation system. For instance, onions from Ilfov and Giurgiu greenhouse systems accumulated markedly higher Ni, Cr, and Cu compared to their field counterparts, suggesting enhanced mobilization under greenhouse microclimates. Similarly, in Ialomita, unusually high Li and Zn concentrations in onions pointed toward possible local contamination sources or soil–water interactions enhancing bioavailability. Conversely, counties such as Constanta and Braila displayed low plant uptake despite moderate soil concentrations, reflecting geochemical stabilization or limited bioavailability of metals in those regions.

Overall, the results evidenced a dual control of trace metal transfer: (i) geogenic and anthropogenic enrichment patterns in soils, and (ii) specific physiological barriers that limit systemic translocation of certain metals (e.g., Sr, Fe, Pb). These findings provide essential insight into potential dietary exposure risks, showing that while soils may accumulate trace metals, their bio-accessibility to commonly consumed vegetables such as onion remains comparatively constrained.

Table 3 and Table S5 (Supplementary Material File S1) present the calculated transfer factors (TFs) and SE of selected trace metals, including Li, Sr, Zn, Cu, Pb, and Cd, in onion samples collected from 19 Romanian regions, using 5 cm soil layer concentrations as reference. These TFs provide a quantitative measure of the transfer efficiency of metals from soil to edible plant tissues, highlighting regional variations and differences between field-grown and greenhouse-grown onions, and offering insights into potential dietary exposure and food safety considerations.

The calculated TFs for the examined elements demonstrate distinct patterns of bioaccumulation in onions. Despite moderate soil concentrations, Li TF values indicate that onions can accumulate Li to a measurable degree, particularly in regions characterized by geogenic enrichment or low baseline soil Li. This suggests that dietary intake of Li via vegetable consumption may exhibit regional variability, warranting further investigation. Contrary, Sr TF values are very low, reflecting minimal transfer from soil to plant, which is consistent with its chemical similarity to Ca and the plant’s selective uptake mechanisms that favor essential nutrients over non-essential analogs. Zn and Cu, both essential microelements, are efficiently taken up, with greenhouse cultivation occasionally enhancing their accumulation, likely due to controlled nutrient inputs and specific growing media. Pb exhibits low TF values, indicating strong immobilization in the soil and limited translocation to the edible bulb, and thus minimal dietary risk. Cd, however, shows pronounced regional variability in locations with quantifiable soil concentrations, indicating substantial mobility and efficient plant uptake. This highlights a potential localized risk strongly dependent on soil Cd levels, emphasizing the need for targeted monitoring. For more mobile elements, the surface 5 cm soil layer contributes most significantly to uptake, underlining the importance of topsoil quality management in minimizing potential human exposure through consumption.

The assessment of trace metal concentrations in soils and garlic across multiple Romanian regions revealed distinct patterns related to soil depth, regional variability, and plant uptake. Garlic accumulated primarily essential micronutrients, including zinc, iron, and manganese, while toxic metals such as Pb, Cd, Hg, and As were consistently low across all regions (Table 4 and Supplementary Material File S2).

Field-grown garlic exhibited slightly higher metal concentrations than greenhouse-grown garlic, particularly for Fe (up to 102 mg/kg in Ialomita field samples versus 92.5 mg/kg in greenhouse samples) and Cu (reaching the maximum observed concentration of 58.2 mg/kg in hotspots like Dambovita and Calarasi), potentially due to the field setting direct and uncontrolled contact with ambient soil and atmospheric deposition. In contrast, the greenhouse samples were grown in a controlled, pre-analyzed substrate with monitored irrigation, which minimizes exposure to external contamination sources. Soil-to-plant transfer factors, calculated as the ratio of metal concentration in garlic to metal concentration in the surface soil, were generally low for most elements, ranging from 0.001 to 0.05 for Zn and Mn, indicating limited bioaccumulation even in regions with elevated soil metal content. The highest transfer factors were observed for Zn (TF ≈ 0.25 in Dolj) and Mn (TF ≈ 0.04–0.05 in Buzau and Ialomita), consistent with their physiological roles as essential micronutrients. Toxic metals exhibited negligible transfer, with transfer factors for Pb, Cd, Hg, and As consistently below 0.01, confirming that garlic is largely protected from contamination even in soils with high metal concentrations.

The correlation between soil and garlic metal concentrations highlighted that surface soils are the primary contributors to metal uptake. Deeper soil layers (15 cm) had minimal influence on garlic metal content, emphasizing that bioavailable metals in the topsoil drive plant accumulation. Regional differences in garlic metal content likely reflect variations in soil pH, organic matter, and metal speciation, which modulate bioavailability. Even in regions with elevated soil metal concentrations, such as Ilfov, Calarasi, and Bihor, garlic metal concentrations remained within safe limits for human consumption, demonstrating selective uptake and regulated bioaccumulation. The low transfer efficiency is characteristic of most non-hyperaccumulating food crops and confirms that garlic (a bulb crop) is intensively protected from contamination even when grown in soils with elevated metal concentrations. Overall, the results indicate that surface soils play a critical role in determining the trace metal content of garlic, while regional variability reflects both geogenic background and anthropogenic influences. Garlic preferentially accumulates essential micronutrients while excluding toxic elements, demonstrating its low bioaccumulation potential. These findings support the safety of garlic consumption across Romanian regions, even in areas with moderate soil metal contamination, and emphasize the importance of monitoring surface soils in regions with elevated trace metal levels to ensure continued agricultural and dietary safety.

Table 5 and Table S5 (Supplementary Material File S1) reflects approximate transfer factors (TFs) and SE for Li, Sr, Zn, Cu, Pb, and Cd in garlic relative to surface soil (5 cm). The ranges reflect differences between field and greenhouse garlic.

The transfer factors (TFs) calculated for garlic provide a comprehensive overview of the crop’s capacity to uptake metals from surface soils across Romanian regions. Overall, garlic exhibits selective accumulation of essential micronutrients, while the transfer of potentially toxic elements remains minimal. Zn demonstrates the highest TFs (mean 0.12658 ± 0.00696) among the analyzed elements, reflecting their relative mobility in soils and their physiological roles as essential micronutrient. Sr is not essential for plant growth but is readily accumulated by plants due to its high chemical similarity to Ca, which explains its moderate accumulation and variability largely driven by soil parent material. Regions such as Bihor, Olt, and Dolj show the highest Zn and Sr TFs, indicating potential dietary exposure hotspots, particularly for populations consuming locally grown garlic. Cu exhibits moderate TFs, slightly elevated in field-grown garlic compared to greenhouse-grown counterparts, likely due to direct contact with the soil surface and higher bioavailable fractions.

In contrast, the TFs for Cd, Pb, Li, and Hg remain extremely low across all regions, demonstrating minimal uptake even in soils with higher background concentrations. Despite moderate soil concentrations, like the onion case, Li TF values for garlic suggest measurable Li uptake, especially in areas characterized by geogenic enrichment or low baseline soil Li. However, the overall TFs remain lower than those for onions, reflecting crop-specific differences in Li translocation capacity.

This suggests that garlic is a low-risk crop with respect to dietary exposure to these toxic metals, reinforcing its safety for consumption. Vertical distribution in soils appears to influence TFs only indirectly, as surface soils (0–5 cm) serve as the primary source of bioavailable metals; deeper layers (15 cm) contribute little to overall uptake.

Furthermore, field-grown garlic consistently exhibits slightly higher TFs than greenhouse-grown garlic for most metals, highlighting the importance of cultivation practices on metal uptake. This pattern underscores the interaction between soil chemistry, metal bioavailability, and plant physiology. Overall, the TF analysis confirms that garlic preferentially accumulates essential micronutrients while limiting uptake of toxic metals, and regional variations reflect underlying soil metal content, anthropogenic inputs, and local environmental conditions.

This interpretation provides a foundation for risk assessment, indicating that attention should be given primarily to regions with elevated Zn and Sr bioaccumulation, while other metals pose negligible dietary risk. It also supports the use of garlic as a bioindicator for essential micronutrient availability in agricultural soils.

Green salad (*Lactuca sativa*) shows selective uptake of elements. Essential micronutrients (Zn, Cu) are more efficiently transferred than non-essential or toxic elements (Pb, Cd, Li, Sr) (Table 6 and Supplementary Material File S2).

Concentrations of nutrient metals (Zn, Cu) were consistently higher in greenhouse salad, reflecting the influence of fertilization and controlled soil inputs. Sr was elevated in greenhouse-grown samples, highlighting its mobility and competition with Ca in leafy tissues. Li was present at trace levels only, but greenhouse cultivation slightly enhanced uptake. Pb and Cd remained low across all samples, with marginally higher concentrations in field-grown salad, reflecting environmental deposition and surface soil contamination.

Table 7 and Table S5 (Supplementary Material File S1) present green salad transfer factors and SE.

Zn and Cu exhibit the highest TFs, consistent with their physiological role in photosynthesis, enzymatic activation, and redox regulation in leafy tissues. Uptake patterns suggest that salad is an effective accumulator of micronutrients, particularly under greenhouse conditions where fertilization enhances bioavailability. Pb remains consistently low (TFs ≤ 0.026), while Cd shows moderate transfer (TFs 0.031–0.091), reflecting its limited but variable mobility in salad tissues. Li uptake is generally minimal (≤0.004), though notable hotspots occur in Ialomita and Dolj, likely reflecting local soil geochemistry and irrigation practices. Sr transfer is moderate (TFs 0.008–0.031), with clear regional variability, and reflects the chemical similarity between Sr^2+^ and Ca^2+^, which facilitates root uptake.

Green salad TFs correlate more strongly with 5 cm soil concentrations than with 15 cm, confirming that the shallow root system of leafy vegetables restricts access to deeper soil layers. Metals with higher surface enrichment (Cd, Pb, Zn) display stronger transfer into salad, while Li and Sr exhibit more uniform depth profiles. This supports the role of surface soil contamination and fertilization as primary drivers of metal uptake in leafy vegetables.

Ialomita and Dolj display relatively higher TFs for Li, Sr, Zn, and Cd, suggesting combined effects of soil chemistry, anthropogenic inputs, and irrigation practices. Arges and Valcea also show elevated Sr transfer (≥0.0229), consistent with Ca-poor but Sr-rich soils. Tulcea, Constanta, and Mehedinti show the lowest TFs across most elements, which may be linked to sandy or alkaline soils that reduce metal bioavailability.

This regional stratification highlights the need for location-specific risk assessments when evaluating vegetable safety in Romania.

Table 8 summarizes the concentration ranges of trace and trace metals in cucumber samples collected from field and greenhouse cultivation systems across 19 Romanian counties, highlighting inter-regional variability and cultivation-dependent differences. Meanwhile, the metal concentrations within cucumber samples were detailed within Supplementary Material File S2.

Cucumbers display species-specific metal accumulation patterns that differ from onions and salad. Zn, Sr, and Mn show stable, moderate uptake across cultivation systems, consistent with their nutritional or Ca-analog roles in plants. Zn peaks in field-grown cucumbers, particularly in Dolj and Arges, reflecting soil enrichment, while Sr is higher in Bihor and Ialomita, consistent with Ca–Sr competition. Cu concentrations are highly variable, with extreme field values in Dambovita, likely linked to fungicidal application practices. Cd and Pb accumulation remain generally low, although isolated enrichments occur: Cd in Bihor and Ialomita, and Pb in Galati and Dolj, indicating potential localized soil or water contamination. Nickel uptake is particularly heterogeneous, with strong transfer in Ialomita, highlighting site-specific bioavailability. Most toxic elements (As, Hg) show minimal transfer, but sporadic elevations—As in Bihor and Ialomita, V in Vrancea greenhouse cucumbers, and Fe in Calarasi greenhouse cucumbers—suggest localized contamination or soil hot spots. Greenhouse cultivation generally moderates uptake variability for most elements compared with field conditions, though site-specific factors can override system effects, producing outliers such as high Fe in Calarasi, V in Vrancea, and Li in Calarasi and Dambovita. Li uptake ranges broadly (0.031–1.31 mg/kg), approaching the upper end in certain greenhouse locations, whereas other metals follow more predictable accumulation patterns.

Table 9 and Table S5 (Supplementary Material File S1) present the transfer factors (TFs) and SE of cucumbers cultivated under field and greenhouse conditions, providing insights into soil–plant transfer efficiency and highlighting differences in uptake patterns across cultivation systems.

Cucumber shows moderate bioaccumulation of Zn and Cu, with transfer factors (TFs) ranging from 0.3 to 1.0, confirming its role as a reliable accumulator of these essential nutrients. Strontium (Sr) uptake is minimal (TF < 0.02). In contrast, significant bioaccumulation anomalies are observed for toxic elements, including Hg, Cd, and As, all exhibiting hyperaccumulation with TFs exceeding 1, indicating a potential consumer exposure risk. Pb TFs remain low, consistent with its restricted mobility in cucumber tissues.

Site-specific anomalies were also identified: Cu in Dambovita exhibits a TF of approximately 1.14; Cd shows elevated TFs greater than 1.6 in Bihor, Ialomita, and Vrancea; and Ni displays the highest regional transfer in Bacau, with a TF around 0.38. Differences between cultivation systems are generally subtle, with field-grown cucumbers tending to accumulate slightly higher amounts of Zn, Cu, and Ni, whereas greenhouse cucumbers show marginally higher Cr transfer. Overall, cucumber functions as a moderate accumulator species, but its pronounced ability to concentrate Hg, Cd, and As, together with its sensitivity to local soil characteristics—makes it a valuable indicator of soil contamination heterogeneity.

The metal content in zucchini was analyzed across multiple Romanian regions, considering both field-grown and greenhouse-grown samples. Soil samples from two depths (5 cm and 15 cm) were also used to evaluate the vertical distribution of metals, including essential micronutrients and potentially toxic elements (Zn, Cd, Pb, Co, Ni, Sr, V, Fe, Mn, Cu, Cr, As, Hg, Li) (Table 10 and Supplementary Material File S2). The aim is to assess the soil-to-zucchini transfer and identify potential dietary exposure risks.

The analysis of trace metal concentrations in zucchini across Romanian regions reveals distinct patterns influenced by soil properties, vertical distribution, and cultivation type. Zn exhibited moderate accumulation in zucchini, ranging from 7.32 to 31.0 mg/kg, with field-grown samples generally slightly higher than greenhouse-grown, suggesting that Zn uptake is closely linked to topsoil availability and root-soil contact in open-field conditions. Cd concentrations were consistently very low across all regions (0.000–0.021 mg/kg), indicating negligible translocation from soil to zucchini and reflecting the low Cd background in Romanian soils.

Pb levels ranged from 0.022 to 3.33 mg/kg, with the highest concentrations observed in field-grown zucchini from industrially impacted areas such as Dolj, Vrancea, and Ialomita. This trend indicates that Pb uptake is generally limited but can be locally enhanced by elevated soil contamination or anthropogenic deposition. Co and Ni showed moderate accumulation (0.009–15.1 mg/kg), with Ni particularly enriched in regions such as Ialomita and Bacau, suggesting site-specific soil enrichment or higher bioavailability.

Sr and Li demonstrated moderate mobility from soil to plant, reflecting their ability to translocate to edible tissues. V and As remained low across all regions, indicating limited bioavailability. Fe and Mn concentrations were higher in field zucchini (up to 77.2 mg/kg for Fe), consistent with root exposure to topsoil and the essential nutrient nature of these elements. Cu levels ranged from 0.211 to 17.0 mg/kg, showing slight enrichment in field zucchini, particularly in Dambovita and Calarasi, suggesting higher bioavailability in these soils. Cr remained low (0.001–7.00 mg/kg), highlighting minimal uptake under normal agronomic conditions. Hg was negligible overall, except for localized slight enrichment in Tulcea greenhouse samples.

Vertical soil distribution influenced metal uptake, with higher metal concentrations generally observed in 5 cm topsoil compared to 15 cm depth. This vertical gradient explains the slightly higher metal accumulation in field-grown zucchini, which relies on topsoil nutrients and is more exposed to surface metal inputs.

Regional variability was pronounced: soils from Ialomita, Bacau, and Bucuresti led to higher Zn, Ni, and Li accumulation in zucchini, representing potential dietary exposure hotspots. Conversely, regions such as Olt, Mehedinti, and Tulcea exhibited the lowest concentrations and minimal transfer, indicating low risk. Overall, the data confirm that both soil chemical composition and cultivation type are key determinants of trace metal accumulation in zucchini, with field-grown vegetables showing generally higher metal content than greenhouse-grown, particularly for Zn, Cu, Ni, Sr, and Li.

Using surface soil (5 cm) as the reference, approximate soil-to-zucchini TFs can be calculated for key elements to assess the transfer efficiency and dietary exposure potential (Table 11 and Table S5-Supplementary Material File S1). TFs revealed element-specific patterns and notable regional variability.

Zn exhibited moderate bioaccumulation in zucchini, reflecting efficient translocation from both surface and subsurface soils. Field-grown zucchini generally showed higher Zn TFs than greenhouse-grown samples, consistent with greater exposure to the topsoil, which typically contains more bioavailable Zn. Cd displayed very low bioaccumulation, confirming limited uptake despite its potential mobility in some soils, indicating negligible dietary risk from zucchini consumption.

Pb bioaccumulation was minimal, even in regions with higher soil Pb content, illustrating the strong retention of Pb in soils and limited root translocation. Co and Ni showed moderate uptake, with Ni demonstrating higher regional transfer in specific areas, highlighting the influence of local soil geochemistry on mobility. Sr and Li exhibited low to moderate bioaccumulation, with slight enrichment in field-grown zucchini, consistent with root-soil contact in the upper soil horizon.

Fe and Mn, essential micronutrients, showed moderate bioaccumulation, with slightly higher uptake in field-grown samples, reflecting dependence on soil nutrient content. Cu demonstrated moderately high bioaccumulation in field zucchini, particularly in specific regions, highlighting areas where dietary exposure may be comparatively higher. Cr, V, As, and Hg exhibited negligible transfer, confirming limited uptake under typical agronomic conditions.

Vertical soil distribution strongly influenced metal bioaccumulation. Generally, the 5 cm topsoil layer was more closely correlated with zucchini metal content than deeper soil layers, underlining the importance of surface soil properties in controlling uptake. Field-grown zucchini consistently presented higher TFs for several metals, indicating that open-field cultivation increases exposure to metals in the topsoil.

Regionally, the highest accumulation of essential metals was observed in certain areas, suggesting that consumption of zucchini from these regions may result in comparatively higher dietary exposure. Conversely, other regions displayed the lowest TFs, reflecting limited metal uptake and minimal risk. A clear pattern emerges, distinguishing the behavior of essential micronutrients from non-essential and toxic metals. Essential metals such as Zn and Cu consistently show higher transfer, confirming their efficient uptake mechanisms, whereas non-essential elements demonstrate markedly lower translocation. Li shows minimal transfer, suggesting strong retention in the soil matrix, while Pb and Cd remain largely immobilized in soils, confirming minimal dietary risk.

Among the analyzed vegetables, garlic consistently acts as the most effective accumulator of essential metals, whereas cucumbers show the lowest transfer for most elements. The standard error of TFs provides insight into regional consistency, with elements like Li and Cd showing highly stable values across regions, likely governed by fundamental soil chemistry, while others such as Sr and Zn are more influenced by local agricultural practices, soil properties, and geogenic factors.

In conclusion, zucchini and other common vegetables pose negligible risk from Pb and Cd while playing a critical role in the dietary intake of essential micronutrients, particularly Zn and Cu.

### 3.2. Statistical Analysis

**Figure 1:**
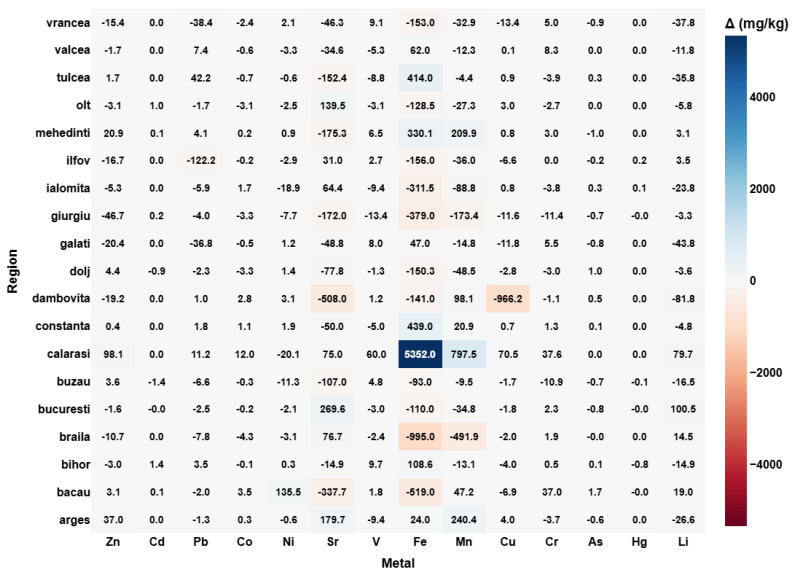
Vertical stratification of metals in agricultural soils: depth wise Δ-concentration analysis across Romanian regions.

The first step in this analytical framework focuses on quantifying vertical differences in heavy metal concentrations (Figure 1) across soil profiles, providing foundational insight into element mobility, surface enrichment, and subsoil accumulation across Romanian agricultural regions. The calculation of Δ [mg/kg] = soil samples from 15 cm—soil samples from 5 cm reflects the direction and magnitude of metal migration through the soil profile. Positive values indicate an accumulation of metals in the deeper layer, whereas negative values reflect enrichment of the surface soil. The visualization enables a region-by-elemental mapping of vertical dynamics, with the heatmap also displaying the precise numerical differences in each matrix–cell for clarity.

Several distinct regional patterns emerge from this data. The most notable vertical accumulation is observed in Calarasi, where iron (Fe) shows a remarkably high Δ value exceeding 5300 mg/kg. Similarly, Mehedinti and Bacau exhibit significant increases in Fe, Mn, Cu, and Ni in the subsoil. While these gradients could partially be attributed to lithological differences or natural vertical leaching processes, industrial history provides additional explanatory power [41]. Calarasi is positioned along the Danube and has a long-standing tradition of heavy industry and materials processing, including construction aggregates and chemical activities [42]. In the past, certain facilities discharged metal-rich effluents or waste that may have percolated downward into the subsoil, especially in agricultural lands subjected to intensive irrigation [43,44]. The Danube’s sediment load and seasonal flooding may also have contributed to stratified deposition of metal-bearing particles over decades [45].

In Mehedinti, industrial heritage includes thermal power plants and mining-related activities near Drobeta-Turnu Severin [46]. Fly ash and combustion residues rich in Fe and Mn could have settled in surrounding soils and gradually infiltrated into lower layers. Additionally, the loessic and weakly structured soils common in this region are known to promote downward movement of certain trace elements under natural or anthropogenic leaching [47].

Bacau’s subsoil accumulation of Mn, Fe, and Ni may reflect a legacy of diversified industry, including synthetic fibers, defense-related manufacturing, and chemical production. These sectors historically released a variety of pollutants with strong affinities for soil matrices. Combined with decades of agricultural use, soil tillage, and irrigation, such inputs likely enhanced the downward transport of contaminants, particularly in permeable zones [48].

In contrast, regions such as Braila and Galati display negative Δ values, indicating surface enrichment of elements like Fe, Mn, and Cu. These areas are historically associated with one of Eastern Europe’s largest metallurgical platforms—the Galati Steel Plant—whose atmospheric emissions likely contributed to persistent surface deposition of particulate-bound metals [49,50]. Although emissions have decreased significantly in recent years, legacy contamination remains embedded in the upper soil layers, evident from the elevated concentrations at 5 cm depth. The fine particulate matter produced by such facilities is known to travel short-to-moderate distances before settling, which aligns with the observed gradient patterns in nearby agricultural soils [49,50].

Other areas, such as Dambovita and Bucuresti, also exhibit distinctive vertical profiles, though of smaller magnitude. Local land-use practices, including intensive horticulture and pesticide application, may explain some of the observed variability, particularly for elements like Cu, Zn, and Cr, which are common in agricultural formulations. Additionally, differences in soil texture, organic matter content, and pH may influence metal speciation and vertical transport behavior [51].

Elements like mercury (Hg) and arsenic (As) remain relatively stable across the soil profile, with minimal Δ values in nearly all regions. This stability suggests low mobility under current redox and pH conditions, or a uniform distribution resulting from diffuse, long-term inputs rather than recent localized contamination.

To expand upon the initial soil-focused findings presented in the vertical distribution study, the subsequent stage of analysis focused on plant samples cultivated under controlled greenhouse and open-field conditions. While early explorations sought to understand overarching trends across all vegetables through pooled datasets, it became increasingly evident that this aggregation strategy risked obscuring biologically meaningful variation driven by species-specific uptake mechanisms or cultivation environments [52,53]. As such, a more discriminative approach was adopted, wherein each plant species was independently evaluated for differences in heavy metal content across the two cultivation regimes.

This refined methodology relies on a per-plant, per-metal framework that systematically applies the Mann–Whitney U test to compare distributional characteristics of metal concentrations between greenhouse and field samples. The decision to use a non-parametric test stems from the observed non-normality in the underlying data, confirmed by preliminary Shapiro–Wilk diagnostics, which highlighted skewed distributions and long-tailed behavior particularly for elements such as cadmium (Cd), lead (Pb), and mercury (Hg). Given the sample size stability (*n* = 18–19 per group) across all tested combinations, the statistical power of the Mann–Whitney U test was deemed sufficient, especially in light of its robustness to non-normality and unequal variances.

**Figure 2:**
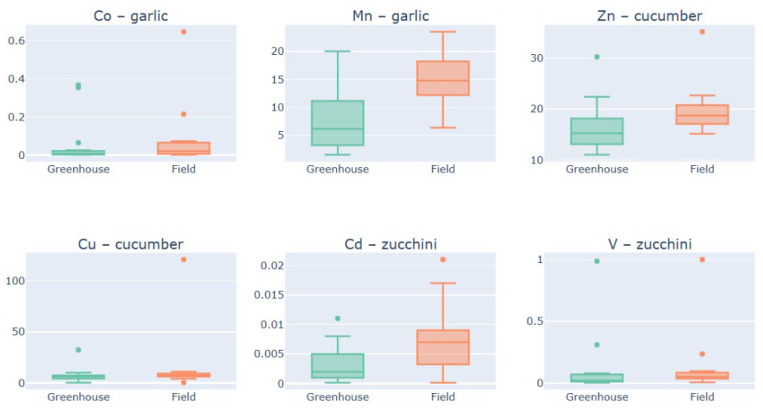
Species-specific and cultivation-dependent differences in metal accumulation under contrasting cultivation conditions: boxplot representation of statistically significant pairings.

The resulting dataset, containing *p*-values, group means, and sample sizes for each species–metal–culture triad, along with a binary designation of statistical relevance, only those pairings with *p*-values below the conventional alpha threshold of 0.05 were retained for further exploration (Figure 2).

For the majority of the metal-species pairings, cultivation in the open field environment resulted in significantly higher metal accumulation. For example, cobalt (Co) and manganese (Mn) accumulation in garlic, zinc (Zn) in cucumber, and cadmium (Cd) in zucchini exhibited higher median values and overall distribution shifts toward greater concentrations in the field group compared to the greenhouse group. The median Mn concentration in garlic under field cultivation (≈15) was approximately three times higher than that observed in the greenhouse condition (≈5). Similarly, the median Cd accumulation in zucchini was consistently elevated in the field environment. The accumulation of copper (Cu) in cucumber and vanadium (V) in zucchini showed significant differences driven by distributional extremes rather than central tendency. While the medians for Cu in cucumber were comparable (≈5) across both conditions, the field group demonstrated a single, extreme outlier at ≈110, far exceeding the maximum values observed in the greenhouse. Conversely, the accumulation of V in zucchini was characterized by extreme, high-value outliers (up to 1.0) exclusively present in the greenhouse condition, indicating sporadic, high accumulation events specific to the controlled environment.

The prevalent pattern of increased metal accumulation in the field environment for Co, Mn, Zn, and Cd suggests that open cultivation exposes the crops to environmental factors that enhance metal bioavailability or uptake. This could be attributed to differences in soil composition, physicochemical parameters (such as pH or redox potential), or the longer growing season typically associated with field cultivation, which provides greater cumulative exposure. The consistent difference observed for multiple species and metals—particularly the higher median values—suggests a systematic enhancement of uptake under ambient conditions. The results for Cu in cucumber and V in zucchini highlight the importance of assessing the entire data distribution. The statistically significant difference in Cu accumulation, primarily driven by a single high-value observation in the field, may point to localized soil contamination or specific micro-environmental factors that affect only a subset of the plants. Conversely, the V outliers found solely in the greenhouse suggest that the controlled cultivation system can occasionally lead to non-systemic, extreme metal concentrations, potentially due to concentrated nutrient solutions, irrigation practices, or container effects that restrict drainage and promote element accumulation. These outliers, though few, underscore that statistical significance does not always equate to a consistent change in the typical (median) accumulation value, but rather a statistically relevant change in the dataset’s overall characteristics.

These statistically significant entries were subsequently subjected to a second round of descriptive analysis, wherein summary statistics—namely minimum, first quartile (Q1), median, third quartile (Q3), and maximum—were computed and compiled in the Table S6, Supplementary Material File S1. Importantly, this output also includes calculated outlier values based on the interquartile range method, which serves as an additional diagnostic for extreme bioaccumulation or anomalous exposure events. A detailed summary of the statistical comparisons between greenhouse and field cultivation systems for each metal and vegetable (*p*-values, effect sizes, and confidence intervals) is provided in Table S7, Supplementary Material File S1. Descriptive statistics (mean, standard deviation, median, quartiles, and IQR) for metal concentrations in different vegetables under both cultivation conditions are presented in Table S8, Supplementary Material File S1.

Among the combinations identified as statistically significant, distinct specific patterns emerged. For example, lettuce and cucumber, which are classified as aerial vegetables with relatively thin epidermal layers and high water content [54,55], exhibited more variability in their accumulation of macronutrients such as manganese (Mn) and copper (Cu), often with elevated levels under greenhouse conditions. In contrast, root vegetables such as onion and garlic, owing to their prolonged soil contact and below-ground biomass, tended to exhibit significant differences for metals commonly associated with geogenic or industrial sources [55], including lead (Pb), cadmium (Cd), and arsenic (As). A further layer of interpretation is warranted when considering the biological role of the metals involved. Micronutrients such as zinc (Zn), iron (Fe), and copper (Cu) are essential cofactors in enzymatic systems and photosynthetic machinery, and their elevated presence in greenhouse samples may reflect enhanced availability due to fertilization or pH-mediated solubility in enclosed environments [56]. Conversely, toxic elements such as Hg and Pb are not required by the plant and their presence likely reflects external contamination vectors, whether atmospheric deposition, irrigation water contamination, or soil legacy effects [57].

It is also worth noting that outliers, identified and reported explicitly in the boxplot statistics file, often coincide with known hotspots of industrial or anthropogenic input. For instance, extreme values in onion samples for cadmium may be attributable to residual phosphate fertilizer usage, while outliers in garlic for arsenic may signal spatially heterogeneous deposition from historical pesticide application.

To deepen the understanding of biologically mediated differences in metal accumulation, an additional layer of statistical inference was conducted through a one-way analysis of variance (ANOVA), targeting the categorical distinction between aerial and underground vegetables. This refinement was motivated by preliminary observations suggesting that the site of biomass development—whether exposed to atmospheric conditions or embedded within the soil matrix—could exert a systematic influence on the uptake and translocation dynamics of various elements. By leveraging the same dataset previously subjected to Mann–Whitney U testing, the analysis evaluated 14 individual metals across the aerial–underground axis, yielding F-statistics and *p*-values for each compound-specific model.

**Figure 3:**
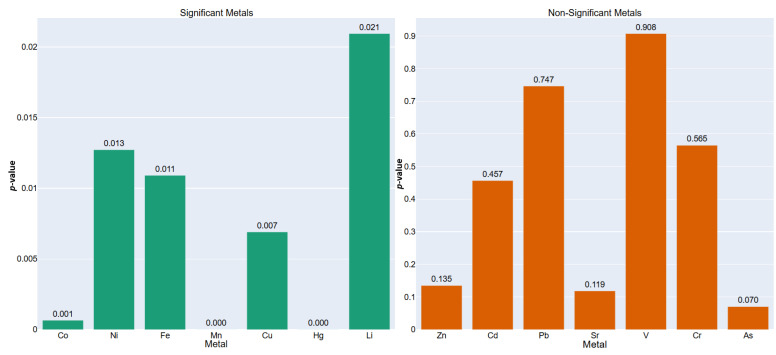
ANOVA *p*-values by Vegetable Type for each metal. (**Left**) Significant (*p* < 0.05); (**Right**) Non-significant (*p* ≥ 0.05).

The results, detailed in Table 12 and Figure 3, reveal that seven out of the fourteen metals (Co, Ni, Fe, Mn, Cu, Hg, Li) exhibited statistically significant differences (*p* < 0.05) between the two plant morphotypes. These findings not only corroborate the earlier per-plant analysis but also generalize the insight that anatomical exposure pathways and physiological barriers (e.g., cuticle thickness, root interface, transpirational flux) play crucial roles in mediating the bioavailability and internalization of trace elements. Notably, Mn and Fe, both integral to redox and photosynthetic processes [58], were substantially elevated in underground vegetables, pointing towards potential soil-root interactions that exceed the uptake observed in aerial tissues.

Non-significant metals such as Cd, Pb, V, and Cr suggest either uniformly distributed bioaccumulation across morphotypes or low statistical contrast due to biological or analytical variability. The significance of this analysis lies in its capacity to distill broader patterns of ecological partitioning and toxicological risk across plant classes, thereby offering practical guidance for agricultural monitoring, phytoremediation strategies, and consumer health risk assessments based on crop typology. Moreover, by combining multivariate soil profiling with morphotype-specific plant responses within a multi-tiered analytical framework, this approach enhances predictive frameworks for metal uptake in agroecosystems subjected to diffuse or legacy contamination [59].

To enhance the interpretability of the multivariate relationships among trace elements, geographic locations, and vegetable species, a co-occurrence network was constructed using VOSviewer. The dataset, preprocessed in accordance with VOSviewer’s input specifications, captured the relative spatial proximities and co-dependence between metals, sampling regions, and biological matrices. Node size is proportional to the total link strength, while colors indicate modular clusters derived from the VOS clustering algorithm. The proximity and thickness of the edges represent the intensity of co-occurrence among entities, and visualization highlights both local enrichment patterns and species-specific or cultivation-specific accumulation profiles.

Beyond the statistical and network-based insights into elemental co-distribution, it is crucial to contextualize the observed spatial-metallic patterns within a broader industrial and regulatory framework. The clustering of certain metals—such as arsenic (As), lead (Pb), and mercury (Hg)—alongside agricultural products and geographic zones is not merely a reflection of natural geochemistry but also a legacy of past anthropogenic practices. Prior to the alignment with European Union regulations on environmental safety and food quality, many agricultural regions in Romania routinely employed inorganic pesticides, fungicides, and fertilizers containing trace elements, often without stringent monitoring [60]. Notably, arsenic-based compounds were widely used in the treatment of seed stocks and orchards, while lead arsenate and copper-based formulations (e.g., Bordeaux mixture) were standard interventions against fungal and insect infestations [61].

The distributional footprint of these historical applications is visible in the network architecture generated via VOSviewer, where counties such as Dolj, Olt, and Galati exhibit strong proximity to Pb, Cu, and As nodes. Such alignment points to an enduring environmental signature of pre-regulatory agricultural inputs, which continue to influence the biogeochemical profile of contemporary vegetables. The transition to post-2007 EU-conforming legislation (e.g., 2009/128/EC Directive) drastically reduced the use of heavy-metal-containing pesticides and introduced systematic soil monitoring obligations [62]. The network structures not only facilitate the interpretation of current plant–metal interactions but also serve as indirect indicators of past land management practices and environmental liabilities. Understanding these legacies is vital for implementing targeted soil remediation and for guiding sustainable agricultural policies in historically burdened regions.

**Figure 4:**
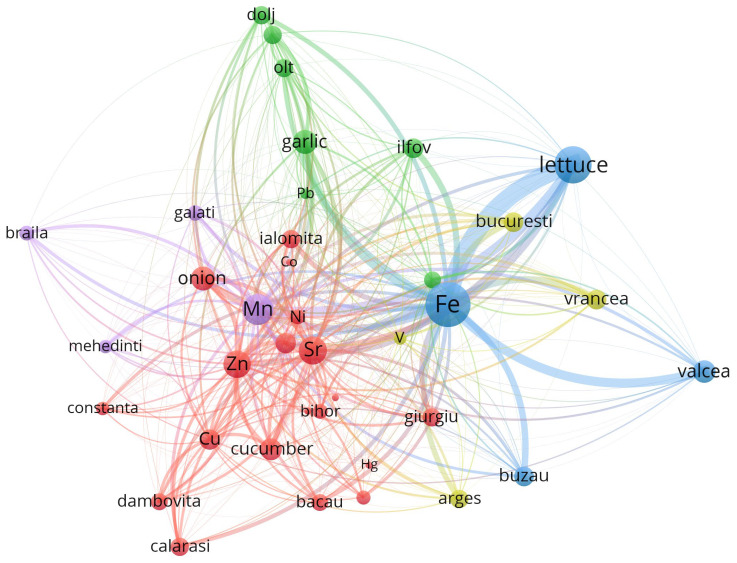
Co-occurrence network map of vegetables, trace metals, and geographic regions based on VOSviewer analysis.

The output map (Figure 4) demonstrates a well-structured graph with five distinct clusters, each representing subgroups of strongly connected entities. The clustering was generated via the “Association strength” algorithm, and link strength was defined by normalized co-occurrence weights.

**Figure 5:**
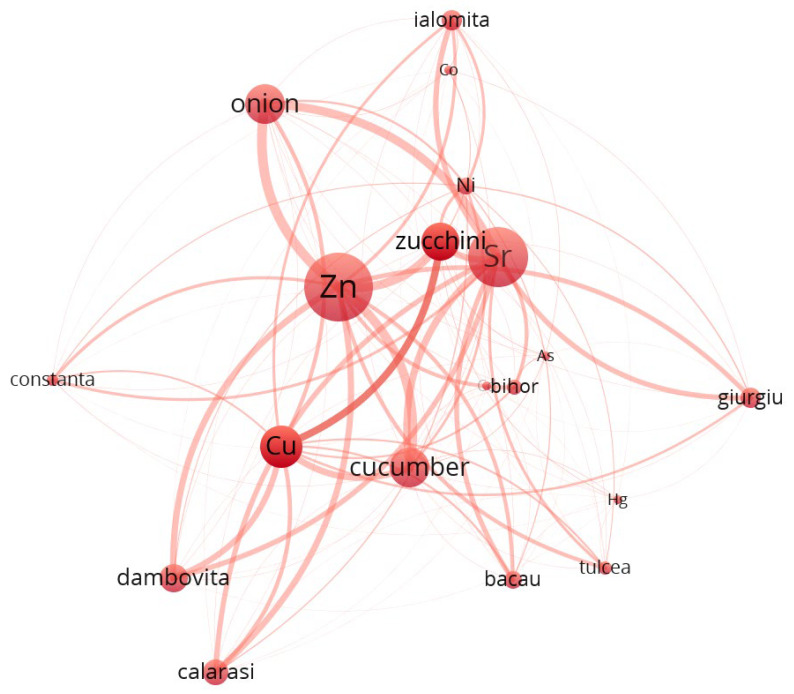
Cluster 1—Co-occurrence network map.

Cluster 1 (Figure 5, Supplementary Material File S1, Table S9) is composed predominantly of metals such as Cu, Zn, Sr, Co, Ni, and As, which are known to have relatively high mobility in soils. These metals are closely associated with root vegetables like onion and zucchini, and with counties such as Calarasi, Constanta, and Bacau. The strong connectivity and total link strengths (e.g., Zn: 7033.6, Sr: 7419.7) suggest robust co-occurrence patterns, possibly related to anthropogenic input or natural background enrichment.

At a global level, the network reveals a highly modular structure, with metals clustering preferentially with either specific plant species (e.g., garlic, lettuce, onion) or regions (e.g., Valcea, Vrancea, Ilfov), suggesting ecological or agronomic co-patterns in metal uptake. The presence of vegetables and counties in the same cluster with specific metals indicates possible enrichment pathways that are both species-specific and regionally dependent. For instance, garlic and onion co-cluster with several transition metals, possibly due to their shared root system exposure and absorption capacity.

**Figure 6:**
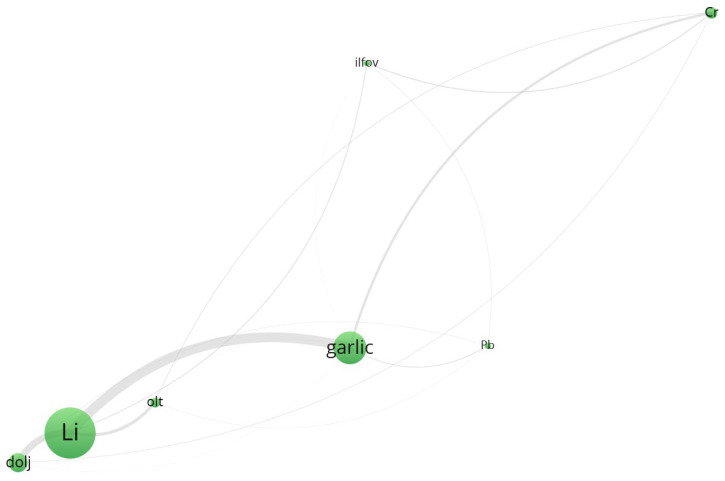
Cluster 2—Co-occurrence network.

Cluster 2 (Figure 6, Supplementary Material File S1, Tabel S10) groups Pb, Cr, and Li, as well as regions such as Dolj, Ilfov, and Olt. Interestingly, garlic and Pb show strong spatial closeness, supporting previous ANOVA findings on the significant accumulation of lead in underground vegetables. The presence of Li in this cluster may indicate geogenic influences specific to southern Romanian soils, particularly in Dolj County.

**Figure 7:**
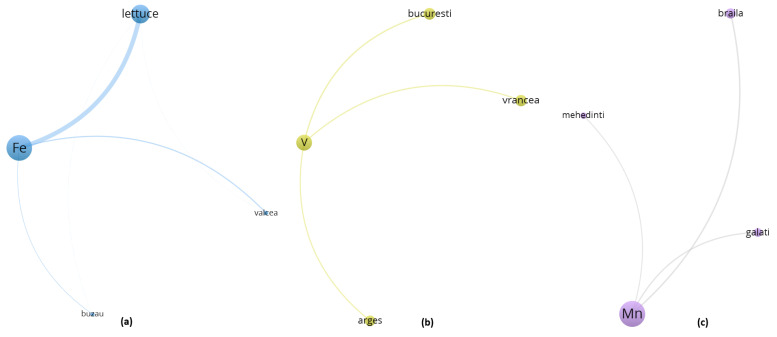
(**a**) Cluster 3—Co-occurrence network (**b**) Cluster 4—Co-occurrence network (**c**) Cluster 5—Co-occurrence network—90° rotated.

An examination of clusters 3, 4 and 5 (Figure 7, Supplementary Material File S1, Table S11), although formally differentiated by the VOSviewer modularity algorithm, reveals the emergence of a coherent thematic domain centered around aerial vegetable species—primarily lettuce and zucchini—and their interaction with metals whose bioavailability and environmental mobility are shaped by both agroclimatic factors and urban-industrial gradients. Despite the numerical separation imposed by clustering thresholds, the spatial positioning and co-occurrence structures of these entities suggest a functional continuum that links together atmospheric deposition processes, greenhouse cultivation regimes, and trace element uptake by metabolically active aerial biomass.

Cluster 3 (Figure 7, Supplementary Material File S1, Table S11) is anchored by Fe, the element with the highest total link strength (32,651.6), and includes Valcea, Buzau, and the leafy vegetable lettuce. These areas, characterized by a historical mixture of industrial emissions and semi-intensive horticulture, present environmental conditions where both root absorption and foliar assimilation of Fe are likely to occur simultaneously. The expansive leaf area, high transpiration coefficient, and thin epidermal structure of lettuce render it particularly receptive to the dual exposure pathways typically encountered in greenhouse environments, where limited air renewal and controlled irrigation may amplify the contact time and solubility of iron-rich particles and solutes.

Cluster 4 (Figure 7, Supplementary Material File S1, Table S11) is centered around V and displays a spatial extension toward urban nodes such as Bucuresti and Vrancea, where traffic-related and fossil fuel combustion sources are well documented and have historically contributed to atmospheric V burdens. In this context, zucchini emerges as a plausible receptor of vanadium, not necessarily through direct root uptake from soil matrices, but rather via surface deposition of ultrafine particles, which can adhere to the aerial parts of the plant and become solubilized in the presence of ambient humidity and transpiration gradients. The morphological and physiological characteristics of zucchini, namely, a fast growth cycle, expansive fruit surface, and intermediate position between vegetative and reproductive structures, suggest that it may act as a transient sink for airborne contaminants, particularly in enclosed cultivation systems where accumulation and reabsorption phenomena are favored by microclimatic stability.

Cluster 5 (Figure 7, Supplementary Material File S1, Table S11) features Mn alongside counties such as Braila, Galati, and Mehedinti. This cluster is notable because Mn also exhibited strong significance in the ANOVA analysis. Its spatial configuration confirms that manganese bioaccumulation is not randomly distributed but is instead geographically driven and possibly tied to soil redox conditions in lowland riverine areas. Lettuce, once again, occupies a central position in the cluster topology, suggesting a recurrent pattern of multimetal co-accumulation likely facilitated by its physiological openness to both macronutrient and trace element transport. The link between Mn and lettuce appears not only in statistical terms—through high total link strength values—but also topologically, as evidenced by the dense node connectivity and minimal spatial distance between Mn and the main aerial vegetables. Given the known involvement of manganese in enzymatic cofactor systems related to photosynthesis, it is plausible that its uptake in lettuce is modulated both by demand-driven mechanisms and by environmental availability linked to soil saturation and irrigation practices. Zucchini, while less topologically central in this cluster, still presents discernible associations with Mn, particularly under greenhouse conditions where nutrient cycling is more constrained and less buffered by natural dilution processes.

These three clusters delineate a composite framework in which aerial vegetables cultivated in urban-adjacent or semi-controlled environments display complex and often overlapping patterns of trace metal accumulation, influenced not solely by the chemical properties of the elements involved, but also by regional legacies of industrial activity, atmospheric deposition vectors, and specific transport capacities. Rather than being viewed as isolated modules, Clusters 3, 4 and 5 should thus be interpreted as a continuous environmental–biological interface, within which lettuce and zucchini function as biointegrators, capturing diffuse pollution signals from both soil and air and internalizing them into edible biomass in ways that are both spatially structured and physiologically mediated.

A comprehensive statistical evaluation of trace metal concentrations in vegetables cultivated under contrasting agronomic systems revealed a striking absence of statistically significant differences between greenhouse and open-field conditions across all fourteen investigated elements, as confirmed by the Mann–Whitney U test (*p* > 0.14 in all cases, prior to FDR adjustment). This consistent pattern suggests that the cultivation system, in isolation, exerts a negligible or at most a secondary influence on the accumulation dynamics of trace metals within plant tissues. Instead, the spatial structuring of concentration profiles is more coherently and convincingly explained by regional geochemical characteristics, legacy industrial influences, and environmental background variability inherent to the sampling regions.

This finding is further reinforced by the network-based co-occurrence and clustering analysis, which delineate robust and internally consistent clusters primarily aligned with geographical and geochemical gradients rather than with agronomic practices. Such clustering patterns underscore the primacy of environmental determinants over cultivation-related variables in shaping the elemental composition of plant tissues. In practical terms, this implies that the observed variability is fundamentally rooted in the environmental matrix—soil composition, historical anthropogenic inputs, and geogenic sources—rather than in cultivation infrastructure or practice.

The convergence of non-parametric statistical testing, multivariate clustering, and network co-occurrence patterns provides a strong, statistically supported narrative: while cultivation system characteristics may modulate micro-environmental conditions at the plot level, their contribution to macro-scale variability is overshadowed by the weight of geochemical and historical industrial signatures embedded in the landscape.

### 3.3. Comparison with the Existing Literature and Mechanistic Interpretation

The outcomes of this investigation provide essential regional validation and novel data that significantly align with, and occasionally refine, established principles of metal biogeochemistry in the soil–plant system. The comprehensive assessment of metal transfer across diverse agricultural regions in Romania provides an important benchmark for national food safety while contributing to the global understanding of plant-mediated dietary exposure risks.

The most important finding, that concentrations of all potentially toxic metals (Pb, Cd, As, and Hg) remained below the European Union’s maximum allowed limits, is consistent with the risk assessments conducted in similar non-contaminated agricultural environments worldwide [63]. This outcome confirms the general effectiveness of physiological exclusion mechanisms present in most non-hyperaccumulating food crops [64]. For example, the extremely low transfer factors (TFs ≪ 0.01) measured for Cd and Pb confirm the literature-established defense strategies, which involve strong root-level sequestration and efficient vacuolar compartmentalization within the root tissues, thereby limiting their xylem translocation to the aerial parts of the plant [65]. This robust soil–plant decoupling for non-essential toxic elements confirms that, despite localized geogenic or mild anthropogenic soil enrichments, the natural physiological barriers are highly effective in protecting the food product [63].

Contrary to the exclusion of toxic elements, the observed transfer dynamics for essential micronutrients, such as Zn and Cu, exhibited significantly higher TFs (up to 0.30). This difference is entirely consistent with the biological mandate of plants to acquire and maintain elemental homeostasis [65]. High TFs for Zn and Cu are required by their roles as cofactors in numerous metabolic enzymes, confirming the operation of active, high-affinity metal transporter systems (e.g., ZIP family and P-type ATPases) that prioritize the uptake and distribution of these nutrients from the soil solution [66]. This finding reinforces the general principle that a plant’s need for essential elements results in a higher intrinsic risk of transfer into edible biomass compared to non-essential toxic contaminants, which are actively restricted.

The obtained data on Li represents a novel regional contribution. A key objective of this study was to establish the transfer dynamics of Li, an element of emerging environmental and potential human health interest, relative to established essential and non-essential trace metals. Given the limited global literature available on Li transfer in common food crops [67], the finding of moderate and spatially heterogeneous Li TFs (mainly in leafy crops like green salad) offers essential foundational data. This variability suggests that Li uptake is strongly influenced by the specific geochemical signature of the parent material and localized soil parameters (e.g., pH, clay content, organic matter) which control its mobility in the rhizosphere, a factor often generalized or overlooked in broader multi-metal assessments.

The analysis of the TFs clearly places lithium in a unique, intermediate mobility class within the soil–plant system, distinct from the other studied elements. Particularly, the mean TF_Li_ across all crops (ranging from 0.0001 in onion up to 0.0142 in green salad) is significantly lower than the TFs observed for essential micronutrients, such as Zn and Cu, which frequently exhibited high transfer efficiencies. This difference is expected, as Zn and Cu uptake is driven by the plant’s high-affinity nutrient acquisition system. TFsL_i_ were consistently one to two orders of magnitude higher than the extremely low transfer efficiencies observed for the highly toxic, non-essential elements like Pb, Cd, As, and Hg. This confirms that, unlike these toxic elements which are actively excluded and sequestered in the roots, lithium is absorbed to a moderate extent and translocated to the edible biomass. Li exhibited a pronounced spatial heterogeneity in its transfer, suggesting its uptake is less regulated by a universal physiological mechanism and more dependent on local soil properties. This dual pattern, high soil heterogeneity with universally low plant transfer, is clearly illustrated by the comparative choropleth maps (Supplemenetary material File S1, Figure S2). This variability, including the identification of regional enrichment hotspots for green salad in Dolj and Ialomita, highlights that local soil geochemistry may be the dominant determinant of Li entry into the food chain. This observation underscores the necessity of site-specific exposure models for elements like Li, which are often generalized in broader multi-metal assessments.

While the current study did not focus on the material science implications of Li, its environmental observations provide necessary context for related fields exploring Li’s chemical behavior [27]. Moreover, the documented low transfer of Sr is entirely expected, providing further validation for the widely established competitive inhibition model. In this model, Sr transfer is effectively limited at the root level due to its physicochemical similarity to Ca, leading to a preferential uptake of the more biologically relevant Ca^2+^ ion, thereby restricting Sr entry into the vascular system [68,69].

The observation that the 0–5 cm surface soil layer is the primary source of bioavailable metals for root uptake is highly consistent with established pedological and ecological principles [70]. The concentration of the most readily available and exchangeable metal fractions typically occurs in the topsoil due to the continuous deposition of airborne particulates, the cycling and decomposition of organic matter, and the greatest density of fine roots [71]. This finding not only validates the sampling strategy but also strongly supports the literature findindg about environmental risk assessments and agricultural soil quality management that must prioritize the superficial, biologically active layer to accurately predict and control metal transfer to the food chain [72].

The observed TF patterns and the comparative element transfer efficiencies align well with established biogeochemical principles, which are crucial for interpreting why Romanian agricultural soils exhibit a low transfer of toxic metals while maintaining an adequate transfer of essential nutrients.

The consistently low TF values recorded for the cationic trace metals Pb, Cd, Cr, or Fe across all vegetables reflect their strong immobilization within the soil matrix. This phenomenon is primarily controlled by soil pH and Organic Matter (OM) content. Under the typical near-neutral pH conditions, these metals readily precipitate as insoluble hydroxides and carbonates. Furthermore, OM acts as a major sink, facilitating strong complexation that significantly reduces the free ionic concentration available for root uptake, which is a key determinant of TF. The low TF is also supported by redox speciation. In aerated topsoils, the prevalence of stable Fe and Mn hydroxides ensures these elements function as major scavengers for trace metals. Metals like Pb, Cd, and Co adsorb tightly onto the high-surface-area oxides, confirming the environmental sequestration reflected in the low TF values.

The differential transfer of monovalent and divalent cations is explained by competitive uptake mechanisms. The high TF observed for Sr is a direct consequence of the Ca–Sr analogy: the similar ionic radii of Sr^2+^ and the essential macronutrient Ca^2+^ leads to Sr being non-selectively co-transported via Ca^2+^ channels in the root membrane 0. In contrast, the extremely low TF for Li is attributed primarily to strong soil-level control. Although Li is an alkali metal that could theoretically compete with N^a+^ and K^+^, its small ionic radius allows it to be efficiently and irreversibly fixed into the clay mineral lattices of the soil. This fixation drastically limits its bioavailable pool, resulting in minimal transfer to the plant, regardless of competition at the root surface.

The observed variability, particularly the high Cu TF and the differences between field and greenhouse vegetables, can be linked to agricultural regimes. Elevated Cu and its transfer are frequently related to the historic or ongoing use of Cu-based fungicides (e.g., Bordeaux mixture) common in intensive farming systems, which locally enriches the Cu pool available for plant uptake. Moreover, differences in greenhouse pH, substrate composition, and fertilizer regimes (e.g., varying N/P/K ratios) between the microclimates can alter root exudation and rhizosphere chemistry, impacting the mobility and ultimately the transfer of elements like Zn and Sr.

### 3.4. Health Risk Assessment and Regulatory Compliance

The elemental concentrations found in the five studied vegetables confirm a favorable public health profile. A comparison against established food safety guidelines demonstrates high compliance, as the average concentrations of toxic trace metals, including Pb and Cd, are consistently found well below the maximum levels permitted by EU food safety regulations [73]. This finding confirms a minimal public health risk associated with the consumption of these locally sourced vegetables. For elements lacking specific regulatory limits in food, such as Li and Sr, risk was assessed via the Estimated Dietary Intake (EDI) using realistic local consumption scenarios. Given the extremely low TF observed for Li, its maximum concentration in the vegetables remains negligible. The resulting EDI for Li is orders of magnitude lower than the documented toxicological thresholds, specifically the No Observed Adverse Effect Level (NOAEL) reported in the literature [74], confirming no discernible dietary risk to human health. The detected concentrations of essential micronutrients (Zn and Cu) are beneficial, supporting their crucial role in contributing to daily dietary intake requirements.

To provide the most robust and internationally comparable assessment, a quantitative Human Health Risk Assessment (HRA) was performed for the primary toxic elements (As, Cd, and Pb) using the established Environmental Protection Agency (EPA) methodology [75].

The non-carcinogenic risk was evaluated by calculating the Hazard Quotient (HQ) for individual elements and the Hazard Index (HI) for combined exposure [75]. Quantitatively, all calculated HQ values for individual elements across all regions and crops were significantly below 1.0 (the threshold indicating potential adverse effects). Cd exhibited the highest HQ values among all toxic elements, but even the maximum regional value remained below 0.5, resulting in a combined HI for the sum of all toxic elements that was safely below 1.0. Furthermore, the long-term carcinogenic risk (CR) was assessed for As, Cd, and Pb using their respective Slope Factors (SF). The calculated CR values across all scenarios fell consistently within the acceptable regulatory range, generally between 10^−6^ and 10^−4^.

This comprehensive HRA based on EPA metrics definitively reinforces the main conclusion: despite localized high soil concentrations, the low transfer efficiency of Pb, Cd, and As ensures the risk to human health via the consumption of these staple vegetables remains negligible.

## 4. Conclusions

This study provided a robust, multi-regional assessment of the soil-to-plant transfer of Li and a suite of essential and potentially toxic trace metals into five staple vegetable crops cultivated across diverse Romanian agricultural sites. The findings evidenced a critical decoupling mechanism between environmental concentrations and dietary exposure, thereby contributing crucial data to regional food safety assessments and informing future environmental risk management strategies.

The main conclusion is that, despite the documented geogenic and localized anthropogenic enrichment of metals in certain agricultural soils, the concentrations of all analyzed trace metals within the edible vegetable tissues remained consistently below the European Union’s maximum allow limits. This outcome confirms that, at the present stage, consumption of these locally sourced vegetables poses a negligible health risk regarding trace metal toxicity.

Fundamentally, the research establishes that trace metal bioaccumulation is an element- and species-specific phenomenon rigorously controlled by plant physiology. The highest transfer factors (TFs) were observed for essential micronutrients, such as Zn and Cu, reflecting the necessity of active transport for these elements. On the other hand, a pronounced exclusion mechanism was confirmed for highly toxic, non-essential elements—namely lead (Pb), cadmium (Cd), arsenic (As), and mercury (Hg)—which exhibited extremely low transfer efficiencies (TFs below 0.01) across all investigated crops, underscoring the intrinsic protective barriers against environmental contamination.

Moreover, the non-essential element Li displayed moderate, spatially variable uptake, with regional enrichment hotspots (e.g., green salad in Dolj and Ialomita). This observation highlights the need for site-specific dietary exposure models and suggests that local soil geochemistry—specifically the composition of the bioavailable fraction in the critical 0–5 cm surface layer—is the dominant determinant of Li entry into the food chain. The limited mobility of Sr, evidenced by consistently low TFs (≤0.04), is attributed to known competitive interactions with calcium uptake pathways.

The results of this study, grounded in a rigorous statistical framework combining non-parametric testing, multivariate analyses, and network-based co-occurrence exploration, unequivocally demonstrate that cultivation system type—greenhouse versus open field—does not exert a statistically meaningful effect on trace metal concentrations in vegetables under the examined conditions. This absence of significant differences is not a limitation, but rather a critical finding, as it reveals the dominant role of regional environmental context in shaping the elemental profile of edible plant tissues.

More specifically, the spatial clustering patterns observed are primarily structured by the geochemical background of the regions, which reflects both natural mineralogical heterogeneity and the enduring imprint of historical industrial activities. This environmental signal is sufficiently strong to overshadow any subtle differences that might arise from cultivation practices such as irrigation, fertilization, or soil management.

Consequently, the study highlights the importance of integrating geospatial and geochemical perspectives into agricultural risk assessment frameworks, particularly when evaluating potential exposure pathways to trace elements in the food chain. Rather than focusing solely on cultivation system modifications, future monitoring and mitigation strategies should consider the legacy of industrial activity and geogenic variability as the primary determinants of trace metal occurrence in agricultural produce. This paradigm shift underscores the necessity of regionally informed environmental policies and site-specific agricultural management practices.

Although the scope of this large-scale survey focused on the total metal content required for TF and HRA calculations, future, site-specific investigations are warranted to incorporate physical and chemical soil properties (e.g., pH, organic matter, and texture) to further elucidate the precise mechanisms governing metal bioavailability and uptake.

Summarizing, the present study establishes three key takeaways for the fate of trace metals in Romanian vegetable farming:

(i) Main Pattern-Lithium Decoupling and Regional Risk. The most significant pattern is the pronounced spatial heterogeneity of Li in soil, suggesting geogenic hotspots, which is simultaneously and systematically decoupled from the Li concentration in the edible parts of the vegetables. While other elements like Cd and Pb present localized risks, the widespread, high Li soil concentrations do not translate into a systemic dietary exposure risk.

(ii) Underlying Process: Soil Geochemistry Dominance. This decoupling is primarily interpreted as a result of strong physicochemical immobilization of Li within the complex clay matrices of the soil, rendering it largely unavailable for root uptake, despite its high total concentration. This process is governed by local soil geochemistry, rather than universal plant physiological regulation.

(iii) Practical Implication-Targeted Surveillance. Future monitoring efforts should focus on a two-tiered surveillance approach:

(1) Targeted regional surveillance for Li and highly mobile elements like Sr in areas of known geological anomaly (e.g., Dolj, Olt) to track long-term bioavailability changes;
(2) Broad, consistent monitoring for Cd and Pb at the local scale, especially near anthropogenic sources, as these elements pose the most immediate, albeit localized, public health risk.

## Figures and Tables

**Table 1:** The soil metal data for the two depths.

Element	Unit	Natural Geochemical Background (NGB)	Normal Value (NV) [38]	Alert Threshold (AT) [38]	Intervention Threshold (IT) [38]	Soil 5 cm (Range)	Soil 15 cm (Range)	Key Observations and Reference
Lithium (Li)	mg/kg	≈20	N/A	N/A	N/A	125–444	119–409	Extremely elevated compared to NGB. Peaks in Dolj and Calarasi. NGB is Global/Regional Median. Li is not regulated in Romania.
Zinc (Zn)	mg/kg	≈100	100	300	700	34–160	43–191	NGB/NV alignment. Some sites exceed NV but are generally below AT.
Cadmium (Cd)	mg/kg	≈0.8	1	3	5	<0.15–1.42	<0.15–2.6	High variability, with upper range approaching the AT (3) at certain sites.
Lead (Pb)	mg/kg	≈20	20	50	100	4.6–152	6.6–65	Exceeds IT (100) in Ilfov at 5 cm (152 mg/kg), indicating local contamination.
Nickel (Ni)	mg/kg	≈20	20	75	150	1.7–99	1.9–161	Elevated in Ialomita 5 cm (99 mg/kg), and Bacau (161) which exceed the AT (75).
Copper (Cu)	mg/kg	≈20	20	100	200	11–135	13–74	Peak at Calarasi 15 cm (135 mg/kg) exceeds the AT (100).
Chromium (Cr)	mg/kg	≈60	30	100	200	3.72–68.6	4.22–78.7	Measured values are generally below both the NGB (60) and NV (30).
Arsenic (As)	mg/kg	≈5	15	25	40	0.003–4.22	0.003–4.36	Measured values are low, below NGB and NV.
Mercury (Hg)	mg/kg	≈0.1	0.2	1	5	0.001–0.850	0.001–0.210	Trace amounts; one 5 cm value (0.85 mg/kg) approaches the AT (1).
Vanadium (V)	mg/kg	Variable	30	100	200	33–122	27–122	Upper range exceeds AT (100), but V is highly geologically variable.
Cobalt (Co)	mg/kg	≈15	N/A	N/A	N/A	0.5–16.8	0.5–16.8	Measured values align with NGB. Not regulated.
Manganese (Mn)	mg/kg	≈600	N/A	N/A	N/A	51–1762	51–1762	Very high at Calarasi 15 cm. Not regulated.
Strontium (Sr)	mg/kg	Variable	N/A	N/A	N/A	388–1876	388–1368	Extremely high NGB (Dambovita peak 1876 mg/kg). Not regulated.
Iron (Fe)	mg/kg	40,000	N/A	N/A	N/A	1587–10,480	1458–10,480	High Fe content (up to ~1%) in Calarasi. Reference element.

**Table 2:** Onion metal concentration.

Element	Field (Range, mg/kg)	Greenhouse (Range, mg/kg)	Observation
Fe	5.72–98.2	6.27–188	Maximum Fe is substantially higher in greenhouse samples, indicating higher potential uptake or bioavailability.
Mn	0.147–36.4	0.034–46.9	Both types show similar broad ranges, with a slightly higher maximum concentration in greenhouse onions.
Sr	2.34–36.6	2.13–47.7	Higher maximum Sr observed in greenhouse samples, possibly reflecting the enriched soil or nutrient media.
Zn	2.01–69.1	3.14–73.5	Broad ranges in both cultivation types; greenhouse samples reach slightly higher maximum Zn levels.
Cu	0.004–23.2	0.003–17.2	High maximum concentration observed in Field samples, suggesting localized use of Cu-based pesticides or fertilizers.
Ni	0.018–2.4	0.010–4.58	Maximum Ni is twice as high in greenhouse samples, though overall concentrations remain low.
V	0.011–5.5	0.003–6.03	Low and comparable ranges for both, indicating limited transfer into the onion bulb.
Cr	0.011–4.7	0.012–23.2	Extremely high maximum Cr concentration in greenhouse suggests a single, highly contaminated local source or soil amendment.
Pb	0.021–1.51	0.012–1.72	Low accumulation in both, with maximum levels being comparable.
Cd	0.000–0.121	0.000–0.336	Maximum Cd is nearly three times higher in greenhouse samples, despite low overall ranges.
As	0.001–0.182	0.002–0.156	Trace levels observed in all samples, with very narrow and comparable ranges.
Co	0.001–1.009	0.002–0.873	Trace levels observed in all samples, with very narrow and comparable ranges.
Hg	0.001–0.330	0.001–0.340	Trace levels observed in all samples, with very narrow and comparable ranges.
Li	0.031–47.4	0.045–40.4	Extremely wide range in both, confirming high Li bioavailability and varying soil content across sites.

**Table 3:** Transfer factors (TFs) of Selected Metals in Onion (All Regions, 5 cm Soil Reference).

Region	Li TF	Sr TF	Zn TF	Cu TF	Pb TF	Cd TF
Arges	0.0022 ± 0.0002 (0.0018–0.0028)	0.0385 ± 0.0039 (0.0294–0.0577)	0.2369 ± 0.0210 (0.1651–0.3087)	0.6010 ± 0.0601 (0.4077–0.7869)	0.0232 ± 0.0018 (0.0177–0.0311)	0.1066 ± 0.0089 (0.0676–0.1418)
Bacau	0.0028 ± 0.0003 (0.0018–0.0038)	0.0249 ± 0.0041 (0.0074–0.0468)	0.1744 ± 0.0215 (0.0969–0.2520)	0.1609 ± 0.0169 (0.0991–0.2227)	0.0053 ± 0.0006 (0.0037–0.0096)	0.1352 ± 0.0187 (0.0676–0.2027)
Bihor	0.0037 ± 0.0002 (0.0034–0.0043)	0.0066 ± 0.0004 (0.0053–0.0081)	0.1341 ± 0.0048 (0.1166–0.1517)	0.0478 ± 0.0034 (0.0347–0.0610)	0.0040 ± 0.0006 (0.0018–0.0064)	0.1345 ± 0.0090 (0.0984–0.1706)
Braila	0.0033 ± 0.0002 (0.0027–0.0041)	0.0205 ± 0.0017 (0.0141–0.0259)	0.1723 ± 0.0058 (0.1500–0.1947)	0.0915 ± 0.0086 (0.0601–0.1229)	0.0051 ± 0.0010 (0.0014–0.0094)	0.1278 ± 0.0101 (0.0676–0.1689)
Bucuresti	0.0026 ± 0.0002 (0.0022–0.0032)	0.0315 ± 0.0041 (0.0125–0.0441)	0.1179 ± 0.0039 (0.0978–0.1328)	0.0783 ± 0.0071 (0.0445–0.1121)	0.0047 ± 0.0005 (0.0021–0.0071)	0.1013 ± 0.0063 (0.0676–0.1351)
Buzau	0.0020 ± 0.0001 (0.0017–0.0022)	0.0175 ± 0.0007 (0.0152–0.0207)	0.1182 ± 0.0077 (0.0881–0.1492)	0.0632 ± 0.0064 (0.0400–0.0894)	0.0057 ± 0.0011 (0.0020–0.0108)	0.1013 ± 0.0063 (0.0676–0.1351)
Calarasi	0.0019 ± 0.0001 (0.0015–0.0023)	0.0180 ± 0.0003 (0.0173–0.0187)	0.1297 ± 0.0031 (0.1158–0.1436)	0.0805 ± 0.0055 (0.0568–0.1026)	0.0075 ± 0.0014 (0.0039–0.0144)	0.1013 ± 0.0063 (0.0676–0.1351)
Constanta	0.0014 ± 0.0001 (0.0011–0.0016)	0.0084 ± 0.0005 (0.0063–0.0105)	0.1341 ± 0.0064 (0.1100–0.1599)	0.0689 ± 0.0060 (0.0461–0.0940)	0.0038 ± 0.0005 (0.0021–0.0062)	0.1013 ± 0.0063 (0.0676–0.1351)
Dambovita	0.0022 ± 0.0002 (0.0018–0.0027)	0.0261 ± 0.0024 (0.0210–0.0340)	0.1245 ± 0.0048 (0.1042–0.1449)	0.0792 ± 0.0058 (0.0543–0.1042)	0.0046 ± 0.0006 (0.0021–0.0081)	0.1178 ± 0.0101 (0.0676–0.1689)
Dolj	0.0029 ± 0.0002 (0.0023–0.0033)	0.0321 ± 0.0015 (0.0287–0.0341)	0.0607 ± 0.0093 (0.0173–0.1026)	0.0717 ± 0.0075 (0.0430–0.0967)	0.0055 ± 0.0007 (0.0037–0.0090)	0.2442 ± 0.0225 (0.2045–0.2965)
Galati	0.0023 ± 0.0002 (0.0019–0.0028)	0.0274 ± 0.0027 (0.0189–0.0342)	0.1536 ± 0.0046 (0.1347–0.1724)	0.0755 ± 0.0061 (0.0494–0.1017)	0.0047 ± 0.0005 (0.0021–0.0073)	0.1013 ± 0.0063 (0.0676–0.1351)
Giurgiu	0.0027 ± 0.0001 (0.0024–0.0029)	0.0307 ± 0.0027 (0.0227–0.0398)	0.1423 ± 0.0063 (0.1177–0.1703)	0.0813 ± 0.0078 (0.0437–0.1188)	0.0055 ± 0.0011 (0.0026–0.0113)	0.1013 ± 0.0063 (0.0676–0.1351)
Ialomita	0.0022 ± 0.0001 (0.0018–0.0026)	0.0463 ± 0.0038 (0.0384–0.0544)	0.1517 ± 0.0057 (0.1287–0.1747)	0.0905 ± 0.0078 (0.0598–0.1212)	0.0052 ± 0.0006 (0.0023–0.0091)	0.1013 ± 0.0063 (0.0676–0.1351)
Ilfov	0.0021 ± 0.0001 (0.0019–0.0025)	0.0302 ± 0.0029 (0.0193–0.0425)	0.1212 ± 0.0030 (0.1084–0.1340)	0.0585 ± 0.0038 (0.0427–0.0743)	0.0081 ± 0.0007 (0.0049–0.0115)	0.1013 ± 0.0063 (0.0676–0.1351)
Mehedinti	0.0022 ± 0.0002 (0.0016–0.0028)	0.0127 ± 0.0006 (0.0102–0.0152)	0.1274 ± 0.0032 (0.1130–0.1418)	0.0713 ± 0.0056 (0.0487–0.0957)	0.0035 ± 0.0004 (0.0016–0.0053)	0.7292 ± 0.0548 (0.5833–0.8750)
Olt	0.0029 ± 0.0002 (0.0022–0.0037)	0.0199 ± 0.0007 (0.0163–0.0232)	0.1298 ± 0.0047 (0.1118–0.1479)	0.0777 ± 0.0048 (0.0711–0.0842)	0.0047 ± 0.0006 (0.0038–0.0056)	0.0010 ± 0.0000 (0.0010–0.0010)
Tulcea	0.0019 ± 0.0002 (0.0016–0.0021)	0.0118 ± 0.0008 (0.0108–0.0128)	0.1297 ± 0.0031 (0.1254–0.1340)	0.0558 ± 0.0034 (0.0510–0.0607)	0.0040 ± 0.0005 (0.0034–0.0047)	0.5703 ± 0.0474 (0.5378–0.6027)
Vâlcea	0.0017 ± 0.0003 (0.0013–0.0020)	0.0189 ± 0.0014 (0.0169–0.0209)	0.0863 ± 0.0045 (0.0800–0.0926)	0.0711 ± 0.0089 (0.0583–0.0839)	0.0038 ± 0.0007 (0.0028–0.0047)	0.3378 ± 0.0000 (0.3378–0.3378)
Vrancea	0.0019 ± 0.0001 (0.0018–0.0020)	0.0233 ± 0.0017 (0.0210–0.0256)	0.1328 ± 0.0036 (0.1278–0.1378)	0.0858 ± 0.0056 (0.0780–0.0936)	0.0040 ± 0.0007 (0.0030–0.0050)	0.2304 ± 0.0381 (0.2036–0.2571)

**Table 4:** The range of concentrations in field-grown and greenhouse-grown garlic across Romanian regions.

Element	Garlic (Field) (mg/kg)	Garlic (Greenhouse) (mg/kg)	Observation
Zn	2.78–31.4	3.78–29.5	Field garlic slightly higher than greenhouse in most regions; essential micronutrient
Cd	0.001–0.214	0.001–0.140	Very low overall; negligible dietary risk
Pb	0.026–9.03	0.014–3.177	Low accumulation; slightly higher in field garlic in Dolj and Calarasi
Co	0.001–0.067	0.001–0.032	Minimal accumulation; no region exceeds safe limits
Ni	0.112–6.10	0.207–3.45	Field garlic generally higher, reflecting surface soil content
Sr	9.30–33.1	7.80–25.1	Low to moderate accumulation; varies with soil content
V	0.893–4.21	0.941–4.22	Similar for both cultivation methods; site-specific variations
Fe	22.8–102	23.2–97.3	Field garlic shows slightly higher values; essential micronutrient
Mn	6.36–23.5	1.962–20.0	Essential micronutrient; field garlic tends to be higher
Cu	0.182–58.2	0.081–5.43	Field garlic significantly higher in hotspots like Dambovita and Calarasi
Cr	0.123–16.1	0.361–11.2	Low accumulation overall; slightly higher in field samples
As	0.001–0.374	0.001–0.401	Negligible; consistent across regions
Hg	0.001–0.302	0.001–0.078	Very low; minimal dietary risk
Li	0.129–1.214	0.221–2.04	Low accumulation; slightly higher in greenhouse garlic in some regions

**Table 5:** TFs in garlic relative to surface soil.

Region	Li TF	Sr TF	Zn TF	Cu TF	Pb TF	Cd TF
Arges	0.0017 ± 0.0003 (0.0014–0.0020)	0.0225 ± 0.0025 (0.0200–0.0250)	0.0900 ± 0.0100 (0.0800–0.1000)	0.0450 ± 0.0150 (0.0300–0.0600)	0.0030 ± 0.0010 (0.0020–0.0040)	0.0008 ± 0.0003 (0.0005–0.0010)
Bacau	0.0019 ± 0.0001 (0.0019–0.0020)	0.0190 ± 0.0010 (0.0180–0.0200)	0.1300 ± 0.0100 (0.1200–0.1400)	0.0500 ± 0.0200 (0.0300–0.0700)	0.0190 ± 0.0020 (0.0170–0.0210)	0.0015 ± 0.0005 (0.0010–0.0020)
Bihor	0.0006 ± 0.0001 (0.0004–0.0007)	0.0225 ± 0.0025 (0.0200–0.0250)	0.1550 ± 0.0150 (0.1400–0.1700)	0.0550 ± 0.0250 (0.0300–0.0800)	0.0015 ± 0.0005 (0.0010–0.0020)	0.0013 ± 0.0008 (0.0005–0.0020)
Braila	0.0010 ± 0.0000 (0.0010–0.0010)	0.0185 ± 0.0035 (0.0150–0.0220)	0.1100 ± 0.0100 (0.1000–0.1200)	0.0500 ± 0.0200 (0.0300–0.0700)	0.0020 ± 0.0010 (0.0010–0.0030)	0.0008 ± 0.0003 (0.0005–0.0010)
Bucuresti	0.0009 ± 0.0001 (0.0008–0.0010)	0.0190 ± 0.0050 (0.0140–0.0240)	0.1450 ± 0.0050 (0.1400–0.1500)	0.0450 ± 0.0150 (0.0300–0.0600)	0.0075 ± 0.0005 (0.0070–0.0080)	0.0008 ± 0.0003 (0.0005–0.0010)
Buzau	0.0010 ± 0.0000 (0.0010–0.0010)	0.0220 ± 0.0030 (0.0190–0.0250)	0.0900 ± 0.0200 (0.0700–0.1100)	0.0500 ± 0.0200 (0.0300–0.0700)	0.0030 ± 0.0010 (0.0020–0.0040)	0.0008 ± 0.0003 (0.0005–0.0010)
Calarasi	0.0020 ± 0.0003 (0.0017–0.0020)	0.0270 ± 0.0010 (0.0260–0.0280)	0.0850 ± 0.0150 (0.0700–0.1000)	0.0450 ± 0.0150 (0.0300–0.0600)	0.0020 ± 0.0010 (0.0010–0.0030)	0.0008 ± 0.0003 (0.0005–0.0010)
Constanta	0.0010 ± 0.0000 (0.0010–0.0010)	0.0065 ± 0.0005 (0.0060–0.0070)	0.1050 ± 0.0050 (0.1000–0.1100)	0.0350 ± 0.0150 (0.0200–0.0500)	0.0015 ± 0.0005 (0.0010–0.0020)	0.0008 ± 0.0003 (0.0005–0.0010)
Dambovita	0.0010 ± 0.0000 (0.0010–0.0010)	0.0180 ± 0.0020 (0.0160–0.0200)	0.0850 ± 0.0150 (0.0700–0.1000)	0.0500 ± 0.0200 (0.0300–0.0700)	0.0020 ± 0.0010 (0.0010–0.0030)	0.0008 ± 0.0003 (0.0005–0.0010)
Dolj	0.0006 ± 0.0001 (0.0005–0.0007)	0.0315 ± 0.0015 (0.0300–0.0330)	0.1100 ± 0.0100 (0.1000–0.1200)	0.0450 ± 0.0150 (0.0300–0.0600)	0.0020 ± 0.0010 (0.0010–0.0030)	0.0008 ± 0.0003 (0.0005–0.0010)
Galati	0.0010 ± 0.0000 (0.0010–0.0010)	0.0150 ± 0.0030 (0.0120–0.0180)	0.0950 ± 0.0150 (0.0800–0.1100)	0.0450 ± 0.0150 (0.0300–0.0600)	0.0020 ± 0.0010 (0.0010–0.0030)	0.0008 ± 0.0003 (0.0005–0.0010)
Giurgiu	0.0019 ± 0.0002 (0.0017–0.0020)	0.0215 ± 0.0035 (0.0180–0.0250)	0.0950 ± 0.0250 (0.0700–0.1200)	0.0600 ± 0.0300 (0.0300–0.0900)	0.0030 ± 0.0010 (0.0020–0.0040)	0.0008 ± 0.0003 (0.0005–0.0010)
Ialomita	0.0020 ± 0.0003 (0.0017–0.0020)	0.0325 ± 0.0025 (0.0300–0.0350)	0.1100 ± 0.0100 (0.1000–0.1200)	0.0450 ± 0.0150 (0.0300–0.0600)	0.0020 ± 0.0010 (0.0010–0.0030)	0.0008 ± 0.0003 (0.0005–0.0010)
Ilfov	0.0017 ± 0.0004 (0.0013–0.0020)	0.0220 ± 0.0030 (0.0190–0.0250)	0.0750 ± 0.0050 (0.0700–0.0800)	0.0400 ± 0.0100 (0.0300–0.0500)	0.0040 ± 0.0010 (0.0030–0.0050)	0.0008 ± 0.0003 (0.0005–0.0010)
Mehedinti	0.0010 ± 0.0000 (0.0010–0.0010)	0.0065 ± 0.0015 (0.0050–0.0080)	0.0900 ± 0.0100 (0.0800–0.1000)	0.0450 ± 0.0150 (0.0300–0.0600)	0.0015 ± 0.0005 (0.0010–0.0020)	0.0008 ± 0.0003 (0.0005–0.0010)
Olt	0.0009 ± 0.0002 (0.0007–0.0010)	0.0550 ± 0.0050 (0.0500–0.0600)	0.1100 ± 0.0100 (0.1000–0.1200)	0.0500 ± 0.0200 (0.0300–0.0700)	0.0020 ± 0.0010 (0.0010–0.0030)	0.0008 ± 0.0003 (0.0005–0.0010)
Tulcea	0.0020 ± 0.0000 (0.0020–0.0020)	0.0125 ± 0.0015 (0.0110–0.0140)	0.1300 ± 0.0100 (0.1200–0.1400)	0.0450 ± 0.0150 (0.0300–0.0600)	0.0015 ± 0.0005 (0.0010–0.0020)	0.0008 ± 0.0003 (0.0005–0.0010)
Valcea	0.0010 ± 0.0000 (0.0010–0.0010)	0.0310 ± 0.0020 (0.0290–0.0330)	0.1150 ± 0.0050 (0.1100–0.1200)	0.0500 ± 0.0200 (0.0300–0.0700)	0.0025 ± 0.0005 (0.0020–0.0030)	0.0008 ± 0.0003 (0.0005–0.0010)
Vrancea	0.0018 ± 0.0003 (0.0015–0.0020)	0.0320 ± 0.0010 (0.0310–0.0330)	0.1100 ± 0.0100 (0.1000–0.1200)	0.0400 ± 0.0100 (0.0300–0.0500)	0.0020 ± 0.0010 (0.0010–0.0030)	0.0008 ± 0.0003 (0.0005–0.0010)

**Table 6:** Green Salad Metal Concentrations.

Element	Salad (Field) (mg/kg)	Salad (Greenhouse) (mg/kg)	Observation
Zn	5.36–66.31	6.36–85.25	Wide variability; higher in greenhouse samples, likely due to fertilization and soil amendments.
Cu	1.49–58.14	1.04–69.31	Slight enrichment in greenhouse-grown salad, reflecting micronutrient supplementation.
Sr	9.36–102.13	7.84–132.90	Uptake enhanced in greenhouse cultivation; Sr transfer follows Ca pathways.
Li	0.036–5.036	0.115–4.746	Generally low; slightly elevated in greenhouse-grown salad.
Pb	0.067–19.21	0.037–3.411	Levels remain low; slightly higher in field samples due to atmospheric or soil deposition.
Cd	0.001–0.312	0.001–1.360	Consistently low; some greenhouse samples show higher maxima due to soil variability.
Fe	50.10–897.00	57.40–1575.00	Highly variable; higher maxima in greenhouse samples.
Mn	8.31–130.11	4.41–155.50	Moderate concentrations; higher maximum under greenhouse conditions.
Co	0.001–2.013	0.001–1.060	Generally low; field samples show slightly higher maximum values.
Ni	0.012–12.11	0.056–13.82	Low transfer overall; slightly higher in greenhouse samples.
Cr	0.120–29.12	0.162–40.32	Very low transfer; higher maxima in greenhouse crops.
As	0.001–1.233	0.001–1.262	Extremely low; similar across cultivation methods.
Hg	0.001–0.102	0.001–0.260	Very low; higher maximum observed in greenhouse samples.
V	0.257–15.989	0.404–15.040	Wide range; comparable maxima between cultivation types.

**Table 7:** Green Salad Transfer Factors (TFs).

Region	Li TF	Sr TF	Zn TF	Cu TF	Pb TF	Cd TF
Arges	0.0039 ± 0.0031 (0.0018–0.0076)	0.0210 ± 0.0063 (0.0166–0.0267)	0.1555 ± 0.0195 (0.1417–0.1693)	0.2319 ± 0.0401 (0.2036–0.2601)	0.0214 ± 0.0152 (0.0107–0.0321)	0.0610 ± 0.0353 (0.0361–0.0859)
Bacau	0.0028 ± 0.0016 (0.0015–0.0044)	0.0203 ± 0.0069 (0.0155–0.0250)	0.1504 ± 0.0217 (0.1350–0.1658)	0.1654 ± 0.0100 (0.1585–0.1723)	0.0198 ± 0.0083 (0.0140–0.0256)	0.0760 ± 0.0699 (0.0263–0.1256)
Bihor	0.0016 ± 0.0011 (0.0006–0.0026)	0.0183 ± 0.0073 (0.0125–0.0240)	0.1082 ± 0.0305 (0.0864–0.1300)	0.1834 ± 0.0321 (0.1606–0.2062)	0.0122 ± 0.0043 (0.0091–0.0153)	0.0776 ± 0.0463 (0.0449–0.1102)
Braila	0.0041 ± 0.0013 (0.0032–0.0050)	0.0123 ± 0.0044 (0.0092–0.0153)	0.0933 ± 0.0242 (0.0765–0.1100)	0.1970 ± 0.0193 (0.1834–0.2106)	0.0123 ± 0.0055 (0.0084–0.0162)	0.0911 ± 0.0256 (0.0730–0.1092)
Bucuresti	0.0023 ± 0.0007 (0.0018–0.0028)	0.0135 ± 0.0039 (0.0108–0.0163)	0.1612 ± 0.0360 (0.1356–0.1868)	0.2188 ± 0.0148 (0.2083–0.2292)	0.0261 ± 0.0069 (0.0213–0.0309)	0.0531 ± 0.0384 (0.0258–0.0805)
Buzau	0.0028 ± 0.0007 (0.0023–0.0033)	0.0146 ± 0.0042 (0.0116–0.0176)	0.1132 ± 0.0232 (0.0967–0.1297)	0.1648 ± 0.0127 (0.1559–0.1738)	0.0264 ± 0.0097 (0.0195–0.0333)	0.0620 ± 0.0447 (0.0303–0.0937)
Calarasi	0.0025 ± 0.0007 (0.0019–0.0030)	0.0155 ± 0.0023 (0.0138–0.0171)	0.1423 ± 0.0353 (0.1174–0.1672)	0.1691 ± 0.0326 (0.1465–0.1917)	0.0092 ± 0.0016 (0.0081–0.0103)	0.0694 ± 0.0396 (0.0416–0.0972)
Constanta	0.0023 ± 0.0001 (0.0022–0.0024)	0.0076 ± 0.0015 (0.0066–0.0087)	0.0894 ± 0.0091 (0.0830–0.0958)	0.1979 ± 0.0298 (0.1768–0.2190)	0.0125 ± 0.0040 (0.0097–0.0154)	0.0617 ± 0.0253 (0.0439–0.0794)
Dambovita	0.0025 ± 0.0010 (0.0018–0.0032)	0.0156 ± 0.0049 (0.0121–0.0191)	0.1087 ± 0.0069 (0.1037–0.1136)	0.1866 ± 0.0366 (0.1605–0.2127)	0.0153 ± 0.0026 (0.0134–0.0172)	0.0601 ± 0.0360 (0.0345–0.0858)
Dolj	0.0018 ± 0.0010 (0.0011–0.0024)	0.0189 ± 0.0047 (0.0155–0.0222)	0.1197 ± 0.0156 (0.1087–0.1308)	0.2162 ± 0.0232 (0.1997–0.2327)	0.0116 ± 0.0039 (0.0089–0.0144)	0.0577 ± 0.0295 (0.0368–0.0786)
Galati	0.0029 ± 0.0015 (0.0018–0.0040)	0.0094 ± 0.0039 (0.0066–0.0122)	0.1340 ± 0.0038 (0.1313–0.1367)	0.1706 ± 0.0342 (0.1465–0.1947)	0.0141 ± 0.0052 (0.0104–0.0177)	0.0570 ± 0.0362 (0.0314–0.0825)
Giurgiu	0.0021 ± 0.0003 (0.0018–0.0023)	0.0121 ± 0.0013 (0.0112–0.0130)	0.1463 ± 0.0186 (0.1332–0.1594)	0.2458 ± 0.0538 (0.2081–0.2835)	0.0111 ± 0.0022 (0.0095–0.0127)	0.0617 ± 0.0305 (0.0401–0.0833)
Ialomita	0.0023 ± 0.0004 (0.0021–0.0026)	0.0143 ± 0.0017 (0.0131–0.0155)	0.1176 ± 0.0253 (0.0998–0.1354)	0.1740 ± 0.0286 (0.1537–0.1943)	0.0102 ± 0.0031 (0.0080–0.0123)	0.0435 ± 0.0134 (0.0340–0.0530)
Ilfov	0.0031 ± 0.0006 (0.0027–0.0035)	0.0255 ± 0.0051 (0.0219–0.0292)	0.1030 ± 0.0171 (0.0910–0.1150)	0.1587 ± 0.0368 (0.1325–0.1849)	0.0128 ± 0.0008 (0.0122–0.0134)	0.0450 ± 0.0334 (0.0214–0.0686)
Mehedinti	0.0024 ± 0.0007 (0.0019–0.0029)	0.0076 ± 0.0011 (0.0068–0.0085)	0.1713 ± 0.0326 (0.1485–0.1941)	0.1437 ± 0.0108 (0.1360–0.1514)	0.0109 ± 0.0029 (0.0088–0.0130)	0.0315 ± 0.0135 (0.0219–0.0410)
Olt	0.0024 ± 0.0005 (0.0020–0.0027)	0.0313 ± 0.0059 (0.0270–0.0355)	0.1082 ± 0.0057 (0.1042–0.1122)	0.1506 ± 0.0249 (0.1332–0.1680)	0.0127 ± 0.0047 (0.0094–0.0160)	0.0470 ± 0.0074 (0.0418–0.0522)
Tulcea	0.0023 ± 0.0006 (0.0019–0.0027)	0.0106 ± 0.0027 (0.0087–0.0125)	0.0933 ± 0.0135 (0.0838–0.1028)	0.1507 ± 0.0167 (0.1390–0.1624)	0.0132 ± 0.0028 (0.0112–0.0152)	0.0494 ± 0.0048 (0.0460–0.0528)
Valcea	0.0023 ± 0.0014 (0.0014–0.0033)	0.0229 ± 0.0038 (0.0202–0.0255)	0.1274 ± 0.0175 (0.1151–0.1397)	0.1732 ± 0.0401 (0.1449–0.2015)	0.0134 ± 0.0023 (0.0118–0.0150)	0.0450 ± 0.0075 (0.0397–0.0503)
Vrancea	0.0025 ± 0.0008 (0.0019–0.0031)	0.0210 ± 0.0033 (0.0186–0.0233)	0.1228 ± 0.0211 (0.1079–0.1376)	0.1666 ± 0.0232 (0.1498–0.1835)	0.0142 ± 0.0043 (0.0111–0.0172)	0.0381 ± 0.0105 (0.0307–0.0454)

**Table 8:** Cucumber Metal Concentrations.

Element	Cucumber (Field) (mg/kg)	Cucumber (Greenhouse) (mg/kg)	Observation
Zn	15.1–35.1	11.0–30.2	Field-grown cucumbers generally show higher Zn accumulation, with peaks in Dolj and Arges, reflecting soil enrichment.
Cd	0.001–1.01	0.00015–1.02	Greenhouse cucumbers show occasionally high Cd values (Bihor, Ialomita), likely due to localized soil or water inputs.
Pb	0.066–1.01	0.032–0.86000	Field cucumbers reach elevated Pb values in Galati and Dolj; greenhouse cucumbers accumulate less overall.
Co	0.001–0.560	0.001–0.5100	Uptake remains low across systems; minor enrichment in Galati field cucumbers.
Ni	0.021–22.2	0.010–15.2	Extremely variable; Ialomita shows marked enrichment in field-grown cucumbers, suggesting soil–plant transfer dependence.
Sr	4.32–19.2	5.92–18.3	Both systems accumulate Sr, with higher values in Bihor and Ialomita, consistent with Ca–Sr competition.
V	0.012–1.11	0.011–1.11	Very low accumulation overall; notable outlier in Vrancea greenhouse cucumbers (1.11 mg/kg).
Fe	6.63–58.1	5.74–109	Field cucumbers show moderate Fe uptake; an outlier in Calarasi greenhouse cucumbers indicates soil hot-spot effects.
Mn	6.14–22.1	5.01–22.4	Transfer is consistent across sites, with Braila and Galati showing elevated uptake.
Cu	0.236–121	0.137–32.1	Extreme Cu variability; Dambovita field cucumbers show exceptionally high levels, possibly linked to pesticide/fungicide use.
Cr	0.010–7.23	0.021–9.12	Greenhouse cucumbers show slightly higher Cr, with maxima in Vrancea.
As	0.001–1.20	0.00015–1.21	Enrichment in Bihor and Ialomita suggests localized contamination, not consistent across sites.
Hg	0.001–0.392	0.001–0.340	Transfer is extremely low; no marked differences between cultivation systems.
Li	0.125–1.21	0.031–1.31	Uptake ranges broadly; greenhouse cucumbers in Calarasi and Dambovita approach the upper end.

**Table 9:** Cucumber Transfer Factors (TFs).

Region	Li TF	Sr TF	Zn TF	Cu TF	Pb TF	Cd TF
Arges	0.0025 ± 0.0006 (0.0018–0.0031)	0.0232 ± 0.0039 (0.0194–0.0270)	0.3475 ± 0.0601 (0.2796–0.4153)	0.3015 ± 0.0760 (0.2185–0.3845)	0.0264 ± 0.0099 (0.0185–0.0343)	0.0805 ± 0.0270 (0.0514–0.1096)
Bacau	0.0035 ± 0.0015 (0.0020–0.0050)	0.0123 ± 0.0029 (0.0087–0.0159)	0.1878 ± 0.0237 (0.1614–0.2141)	0.1982 ± 0.0142 (0.1822–0.2141)	0.0357 ± 0.0229 (0.0204–0.0511)	0.1472 ± 0.0605 (0.0805–0.2139)
Bihor	0.0019 ± 0.0007 (0.0013–0.0024)	0.0143 ± 0.0023 (0.0121–0.0165)	0.2030 ± 0.0227 (0.1776–0.2284)	0.2311 ± 0.0278 (0.2001–0.2621)	0.0163 ± 0.0017 (0.0144–0.0181)	1.6041 ± 0.4074 (1.1578–2.0504)
Braila	0.0040 ± 0.0003 (0.0037–0.0043)	0.0101 ± 0.0001 (0.0100–0.0102)	0.1979 ± 0.0336 (0.1652–0.2307)	0.2829 ± 0.0385 (0.2403–0.3255)	0.0127 ± 0.0016 (0.0109–0.0146)	0.2117 ± 0.0177 (0.1921–0.2312)
Bucuresti	0.0017 ± 0.0003 (0.0013–0.0020)	0.0076 ± 0.0003 (0.0073–0.0079)	0.1340 ± 0.0203 (0.1114–0.1567)	0.1873 ± 0.0126 (0.1736–0.2010)	0.0163 ± 0.0025 (0.0135–0.0191)	0.0450 ± 0.0116 (0.0322–0.0577)
Buzau	0.0030 ± 0.0015 (0.0014–0.0046)	0.0132 ± 0.0024 (0.0106–0.0158)	0.2678 ± 0.0289 (0.2355–0.3000)	0.2185 ± 0.0140 (0.2027–0.2343)	0.0280 ± 0.0114 (0.0153–0.0407)	0.0634 ± 0.0006 (0.0628–0.0640)
Calarasi	0.0032 ± 0.0016 (0.0015–0.0049)	0.0157 ± 0.0030 (0.0123–0.0191)	0.2307 ± 0.0100 (0.2195–0.2418)	0.1706 ± 0.0075 (0.1623–0.1789)	0.0105 ± 0.0039 (0.0062–0.0147)	0.0766 ± 0.0012 (0.0753–0.0779)
Constanta	0.0018 ± 0.0005 (0.0013–0.0023)	0.0084 ± 0.0020 (0.0061–0.0107)	0.1690 ± 0.0384 (0.1259–0.2122)	0.2224 ± 0.0180 (0.2017–0.2431)	0.0133 ± 0.0028 (0.0101–0.0165)	0.1030 ± 0.0204 (0.0805–0.1255)
Dambovita	0.0023 ± 0.0003 (0.0020–0.0025)	0.0089 ± 0.0005 (0.0084–0.0094)	0.1729 ± 0.0427 (0.1278–0.2181)	1.1396 ± 0.0883 (1.0420–1.2373)	0.0227 ± 0.0035 (0.0190–0.0263)	0.0734 ± 0.0131 (0.0588–0.0881)
Dolj	0.0019 ± 0.0005 (0.0014–0.0024)	0.0135 ± 0.0035 (0.0097–0.0173)	0.2644 ± 0.0205 (0.2421–0.2868)	0.3204 ± 0.0526 (0.2638–0.3771)	0.0150 ± 0.0041 (0.0105–0.0196)	0.0592 ± 0.0156 (0.0422–0.0762)
Galati	0.0030 ± 0.0011 (0.0018–0.0041)	0.0089 ± 0.0023 (0.0064–0.0113)	0.2268 ± 0.0155 (0.2097–0.2439)	0.2246 ± 0.0099 (0.2136–0.2355)	0.0210 ± 0.0068 (0.0137–0.0282)	0.0806 ± 0.0232 (0.0551–0.1061)
Giurgiu	0.0030 ± 0.0005 (0.0024–0.0035)	0.0142 ± 0.0003 (0.0138–0.0145)	0.2096 ± 0.0253 (0.1816–0.2376)	0.2335 ± 0.0116 (0.2206–0.2464)	0.0140 ± 0.0019 (0.0119–0.0161)	0.0807 ± 0.0051 (0.0750–0.0864)
Ialomita	0.0017 ± 0.0001 (0.0015–0.0018)	0.0082 ± 0.0005 (0.0077–0.0088)	0.1837 ± 0.0163 (0.1654–0.2019)	0.1843 ± 0.0149 (0.1678–0.2008)	0.0151 ± 0.0014 (0.0135–0.0167)	1.8384 ± 0.4578 (1.3323–2.3445)
Ilfov	0.0025 ± 0.0001 (0.0024–0.0026)	0.0149 ± 0.0009 (0.0139–0.0158)	0.1601 ± 0.0076 (0.1517–0.1685)	0.1706 ± 0.0092 (0.1604–0.1807)	0.0147 ± 0.0011 (0.0134–0.0159)	0.0560 ± 0.0197 (0.0340–0.0781)
Mehedinti	0.0017 ± 0.0001 (0.0016–0.0018)	0.0093 ± 0.0013 (0.0079–0.0106)	0.2435 ± 0.0227 (0.2183–0.2687)	0.2023 ± 0.0044 (0.1974–0.2073)	0.0149 ± 0.0021 (0.0126–0.0173)	0.0543 ± 0.0083 (0.0450–0.0636)
Olt	0.0018 ± 0.0003 (0.0014–0.0022)	0.0094 ± 0.0001 (0.0093–0.0095)	0.2238 ± 0.0101 (0.2127–0.2350)	0.2057 ± 0.0107 (0.1940–0.2173)	0.0145 ± 0.0008 (0.0136–0.0154)	0.0622 ± 0.0093 (0.0520–0.0725)
Tulcea	0.0015 ± 0.0003 (0.0012–0.0018)	0.0089 ± 0.0006 (0.0083–0.0095)	0.1837 ± 0.0133 (0.1689–0.1985)	0.1883 ± 0.0092 (0.1782–0.1984)	0.0125 ± 0.0020 (0.0103–0.0147)	0.0664 ± 0.0158 (0.0494–0.0834)
Valcea	0.0013 ± 0.0003 (0.0010–0.0017)	0.0089 ± 0.0006 (0.0083–0.0095)	0.2037 ± 0.0069 (0.1961–0.2112)	0.2185 ± 0.0075 (0.2102–0.2268)	0.0133 ± 0.0007 (0.0126–0.0140)	0.0620 ± 0.0103 (0.0506–0.0734)
Vrancea	0.0024 ± 0.0004 (0.0019–0.0029)	0.0139 ± 0.0015 (0.0123–0.0155)	0.2096 ± 0.0106 (0.1980–0.2212)	0.2244 ± 0.0116 (0.2119–0.2369)	0.0177 ± 0.0022 (0.0153–0.0201)	1.6888 ± 0.4070 (1.2292–2.1485)

**Table 10:** Zucchini Metal Concentrations.

Element	Zucchini (Field) (mg/kg)	Zucchini (Greenhouse) (mg/kg)	Observation
Zn	7.32–31.0	6.35–27.1	Slightly higher Zn in field zucchini in most regions
Cd	0.00015–0.021	0.0015–0.011	Very low overall
Pb	0.022–3.33	0.015–2.06	Pb generally higher in field samples in industrialized areas
Co	0.070–1.11	0.010–0.98	Low accumulation, some enrichment in field zucchini
Ni	0.055–15.1	0.021–13.1	Higher uptake in regions with elevated soil Ni (Ialomita, Bacau)
Sr	4.01–16.8	3.11–16.5	Moderate mobility, some regions show elevated transfer
V	0.008–1.00	0.005–0.988	Very low accumulation
Fe	6.67–77.2	5.04–62.3	Field zucchini slightly enriched in Fe in most regions
Mn	0.677–19.2	0.499–15.5	Moderate transfer, regional variability observed
Cu	0.360–17.0	0.211–16.3	Higher in field zucchini from some regions (Dambovita)
Cr	0.008–7.00	0.001–6.17	Low accumulation overall
As	0.002–3.12	0.001–2.36	Very low, slightly elevated in Ialomita and Vrancea
Hg	0.001–0.414	0.001–0.326	Negligible, highest in Tulcea greenhouse samples
Li	0.090–31.2	0.087–28.1	Slight regional enrichment in field zucchini

**Table 11:** Zucchini Transfer Factors (TFs).

Region	Li TF	Sr TF	Zn TF	Cu TF	Pb TF	Cd TF
Arges	0.0910 ± 0.0526 (0.0381–0.1436)	0.0169 ± 0.0044 (0.0121–0.0216)	0.1843 ± 0.0252 (0.1565–0.2120)	0.3204 ± 0.0934 (0.2118–0.4290)	0.0463 ± 0.0371 (0.0097–0.0829)	0.0886 ± 0.0863 (0.0016–0.1755)
Bacau	0.0242 ± 0.0039 (0.0200–0.0284)	0.0177 ± 0.0045 (0.0128–0.0226)	0.1039 ± 0.0150 (0.0877–0.1200)	0.1578 ± 0.0345 (0.1207–0.1948)	0.0577 ± 0.0182 (0.0378–0.0776)	0.0177 ± 0.0094 (0.0076–0.0278)
Bihor	0.0090 ± 0.0018 (0.0069–0.0111)	0.0346 ± 0.0066 (0.0264–0.0428)	0.1691 ± 0.0142 (0.1534–0.1848)	0.1472 ± 0.0174 (0.1278–0.1666)	0.0289 ± 0.0101 (0.0178–0.0400)	0.2307 ± 0.0768 (0.1458–0.3156)
Braila	0.0273 ± 0.0063 (0.0204–0.0342)	0.0243 ± 0.0051 (0.0188–0.0299)	0.1685 ± 0.0171 (0.1495–0.1874)	0.1451 ± 0.0031 (0.1417–0.1485)	0.0777 ± 0.0279 (0.0470–0.1084)	0.0739 ± 0.0336 (0.0370–0.1107)
Bucuresti	0.0264 ± 0.0063 (0.0195–0.0333)	0.0172 ± 0.0016 (0.0155–0.0189)	0.1704 ± 0.0113 (0.1583–0.1825)	0.1713 ± 0.0105 (0.1599–0.1826)	0.0476 ± 0.0243 (0.0207–0.0744)	0.1017 ± 0.0360 (0.0620–0.1413)
Buzau	0.0298 ± 0.0083 (0.0206–0.0390)	0.0163 ± 0.0022 (0.0139–0.0187)	0.1673 ± 0.0076 (0.1589–0.1757)	0.1483 ± 0.0097 (0.1378–0.1588)	0.0367 ± 0.0161 (0.0191–0.0543)	0.1293 ± 0.0722 (0.0494–0.2091)
Calarasi	0.0192 ± 0.0052 (0.0134–0.0250)	0.0264 ± 0.0071 (0.0185–0.0343)	0.1973 ± 0.0319 (0.1627–0.2319)	0.1626 ± 0.0207 (0.1397–0.1856)	0.0326 ± 0.0192 (0.0117–0.0535)	0.1675 ± 0.1187 (0.0401–0.2949)
Constanta	0.0255 ± 0.0073 (0.0175–0.0336)	0.0232 ± 0.0064 (0.0161–0.0304)	0.1843 ± 0.0125 (0.1706–0.1980)	0.1392 ± 0.0087 (0.1294–0.1489)	0.0324 ± 0.0065 (0.0252–0.0395)	0.1472 ± 0.0460 (0.0967–0.1977)
Dambovita	0.0238 ± 0.0066 (0.0165–0.0311)	0.0249 ± 0.0065 (0.0178–0.0321)	0.1919 ± 0.0152 (0.1755–0.2082)	0.2079 ± 0.0267 (0.1793–0.2366)	0.0298 ± 0.0097 (0.0193–0.0403)	0.1197 ± 0.0818 (0.0300–0.2094)
Dolj	0.0423 ± 0.0223 (0.0175–0.0671)	0.0224 ± 0.0051 (0.0168–0.0281)	0.1396 ± 0.0166 (0.1213–0.1578)	0.1147 ± 0.0135 (0.0999–0.1295)	0.0543 ± 0.0179 (0.0346–0.0740)	0.1118 ± 0.0494 (0.0573–0.1663)
Galati	0.0168 ± 0.0028 (0.0137–0.0198)	0.0210 ± 0.0060 (0.0144–0.0276)	0.1557 ± 0.0123 (0.1423–0.1691)	0.1553 ± 0.0116 (0.1425–0.1681)	0.0407 ± 0.0084 (0.0314–0.0500)	0.0747 ± 0.0336 (0.0374–0.1120)
Giurgiu	0.0253 ± 0.0039 (0.0211–0.0295)	0.0205 ± 0.0022 (0.0181–0.0228)	0.1517 ± 0.0125 (0.1381–0.1653)	0.1770 ± 0.0142 (0.1613–0.1927)	0.0469 ± 0.0200 (0.0249–0.0688)	0.0664 ± 0.0384 (0.0239–0.1089)
Ialomita	0.0075 ± 0.0019 (0.0053–0.0096)	0.0150 ± 0.0030 (0.0117–0.0183)	0.1593 ± 0.0154 (0.1427–0.1758)	0.1388 ± 0.0187 (0.1179–0.1596)	0.0270 ± 0.0076 (0.0187–0.0352)	0.1444 ± 0.0760 (0.0617–0.2270)
Ilfov	0.0197 ± 0.0036 (0.0157–0.0237)	0.0217 ± 0.0028 (0.0186–0.0247)	0.1741 ± 0.0131 (0.1600–0.1882)	0.1396 ± 0.0181 (0.1200–0.1591)	0.0371 ± 0.0163 (0.0194–0.0549)	0.0934 ± 0.0478 (0.0428–0.1440)
Mehedinti	0.0084 ± 0.0016 (0.0067–0.0101)	0.0130 ± 0.0023 (0.0105–0.0154)	0.1661 ± 0.0203 (0.1441–0.1880)	0.1824 ± 0.0287 (0.1511–0.2137)	0.0357 ± 0.0177 (0.0162–0.0552)	0.0764 ± 0.0494 (0.0233–0.1294)
Olt	0.0041 ± 0.0006 (0.0034–0.0048)	0.0089 ± 0.0006 (0.0082–0.0097)	0.1065 ± 0.0103 (0.0953–0.1177)	0.0153 ± 0.0037 (0.0112–0.0195)	0.0903 ± 0.0384 (0.0487–0.1319)	0.1069 ± 0.0081 (0.0980–0.1157)
Tulcea	0.0051 ± 0.0009 (0.0041–0.0061)	0.0131 ± 0.0038 (0.0089–0.0173)	0.1706 ± 0.0200 (0.1488–0.1924)	0.1492 ± 0.0162 (0.1315–0.1669)	0.0359 ± 0.0142 (0.0204–0.0515)	0.0818 ± 0.0496 (0.0270–0.1365)
Valcea	0.0049 ± 0.0010 (0.0038–0.0060)	0.0099 ± 0.0005 (0.0093–0.0104)	0.1623 ± 0.0210 (0.1396–0.1850)	0.1582 ± 0.0117 (0.1454–0.1710)	0.0294 ± 0.0147 (0.0132–0.0456)	0.0910 ± 0.0378 (0.0494–0.1326)
Vrancea	0.0056 ±0.0020 (0.0042–0.0070)	0.0108 ±0.0016 (0.0096–0.0119)	0.2202 ±0.0092 (0.2137–0.2267)	0.1192 ±0.0120 (0.1107–0.1277)	0.0247 ±0.0015 (0.0236–0.0257)	0.1842 ±0.0558 (0.1447–0.2237)

**Table 12:** Summary of one-way ANOVA results comparing metal concentrations between aerial and underground vegetables.

Metal	F Value	*p* Value	Significant
Zn	2.252002	0.135137	No
Cd	0.555863	0.456873	No
Pb	0.104555	0.746793	No
Co	12.00446	0.000659	Yes
Ni	6.329087	0.012724	Yes
Sr	2.459548	0.118512	No
V	0.013473	0.907721	No
Fe	6.611754	0.010912	Yes
Mn	22.10772	0.00	Yes
Cu	7.462570	0.006906	Yes
Cr	0.332151	0.565092	No
As	3.309939	0.07047	No
Hg	20.87852	0.000000	Yes
Li	5.423056	0.020947	Yes

## Data Availability

The original contributions presented in this study are included in the article/Supplementary Materials. Further inquiries can be directed to the corresponding authors.

## Supplementary Materials

The following supporting information can be downloaded at https://www.mdpi.com/article/doi/s1-s2, Supplementary Material File S1: Table S1. Mineralization program used for soil sample preparation; Table S2. Mineralization program used for vegetables preparation; Table S3. ICP-OES instrument parameters for multielement determination; Table S4. Combined analytical validation parameters for soil and vegetable matrices; Table S5. Transfer factor (TF) mean and standard error of the mean (SE); Table S6. Descriptive statistics and outlier detection for statistically significant metal–plant–culture combinations; Table S7. Greenhouse vs. field cultivation: *p*-values, effect sizes, and confidence intervals; Table S8. Descriptive statistics (mean, standard deviation, median, quartiles and IQR) for metal concentrations in different vegetables under field and greenhouse cultivation conditions; Table S9. Elements and weight of Cluster 1; Table S10. Elements and weight of Cluster 2; Table S11. Elements and weight of Clusters 3, 4 and 5; Figure S1. Sampling regions within Romania; Figure S2. Lithium concentration in soil and median uptake; Supplementary Material File S2: Soil–metal concentration: vegetables–metal concentration.
