# Supplementary material for: From Soil to Plate: Lithium and Other Trace Metals Uptake in Vegetables Under Variable Soil Conditions
Source: Toxics. 2025 Nov 5;13(11):956. doi: 10.3390/toxics13110956 (PMC12656363; doi:10.3390/toxics13110956)
Supplement: Supplementary file 1 [file toxics-13-00956-s001.zip › toxics-3937158-supplementary-11-3 - review/Supplementary Material_1.pdf]

- *Supplementary Material_1* –

From Soil to Plate: Lithium and Other Trace Metals Uptake in Vegetables under Variable Soil Conditions

Nadia Paun [1], Ramona Zgavarogea [1], Violeta-Carolina Niculescu [1]*, Ana Maria Nasture [1], Iulian Voicea [2] and Diana Ionela Popescu (Stegarus) [1]*

[1] National Research and Development Institute for Cryogenic and Isotopic Technologies—ICSI Ramnicu Valcea, 4th Uzinei Street, P.O. Box Raureni 7, 240050 Ramnicu Valcea, Romania; nadia.paun@icsi.ro (N.P.), ramona.zgavarogea@icsi.ro (R.Z.),
[2] National Institute of Research—Development for Machines and Installations Designed for Agriculture and Food Industry, 013813 Bucharest, Romania, voicea@inma.ro (I.V.);
* Correspondence: violeta.niculescu@icsi.ro: diana.stegarus@icsi.ro

Table S1. Mineralization program used for soil samples preparation

Parameter	Microwave Power (W)	Ramp Time (min)	Pressure (psi)	Holding Temperature (°C)	Holding Time (min)
Value	900–1800	10	100	175	15

Table S2. Mineralization program used for vegetables preparation

Parameter	Microwave Power (W)	Ramp Time (min)	Pressure (psi)	Holding Temperature (°C)	Holding Time (min)
Value	900–1800	100	15	200	15

Table S3. ICP-OES instrument parameters for multielement determination

Parameters/Element	Wavelength (nm)	Gas type	Plasma view	Calibration range, soil matrix	Calibration range, vegetables matrix
**Zn**	213.856	Ar	Axial	0.5 – 3.0 mg/L	0.5–25.0 µg/L
**Pb**	220.353	Ar	Axial		
**As**	193.698	Ar	Axial		
**Co**	228.615	Ar	Axial		
**V**	309,311	Ar	Axial		
**Ni**	231,604	Ar	Axial		
**Cd**	226,502	Ar	Axial		
**Li**	670,791	Ar	Radial		
**Hg**	194.159	Ar	Axial		
**Mn**	257,610	Ar	Axial		
**Cu**	324,754	Ar	Axial		
**Cr**	267,716	Ar	Axial		
**Sr**	407,771	Ar	Radial		
**Fe**	259.940	Ar	Axial		

Table S4. Combined analytical validation parameters for soil and vegetable matrices

Matrix	Element	R^2	Standards	Matrix Match	LOD (mg/L)	LOQ (mg/L)	Precision (%RSDr)	Uncertainty (%)	Recovery (%)	Spectral Interference Correction
Soil	Zn	0.99967	5	Yes	0.0082	0.0247	2.8931	9.0	104	Corrected by spectral separation and background correction
	Cd	0.99996	5	Yes	0.00177	0.0053	0.3341	14.0	110	No correction required
	Pb	0.99996	5	Yes	0.0254	0.0763	3.3467	8.0	97.8	No correction required
	Co	0.99995	5	Yes	0.00349	0.0104	6.0489	11.0	96.6	No correction required
	Ni	0.99986	5	Yes	0.00438	0.0131	1.2334	4.0	102	No correction required
	Sr	0.99997	5	Yes	0.00668	0.0201	1.2376	10.0	98.1	No correction required
	V	0.99998	5	Yes	0.01183	0.03548	1.2214	12.0	91.6	No correction required
	Fe	0.99869	5	Yes	0.02192	0.08177	6.3612	14.0	98.7	Background corrected by background shift; evaluated with blank matrix solution
	Mn	0.99997	5	Yes	0.00176	0.00529	4.5702	9.0	99.8	No correction required
	Cu	0.99986	5	Yes	0.00337	0.01011	3.0348	7.0	101	Corrected by spectral separation and background correction
	Cr	0.99996	5	Yes	0.00192	0.00576	2.5615	6.0	107	No correction required
	As	0.99955	5	Yes	0.02391	0.07174	6.1413	13.0	102	No correction required
	Hg	0.99818	5	Yes	0.03671	0.1101	5.6606	12.0	108	Background corrected by background shift; evaluated with blank matrix solution
	Li	0.99995	5	Yes	0.00526	0.01579	3.9938	9.0	101	No correction required
Vegetables	Zn	0.99991	5	Yes	0.00095	0.00285	2.8031	8.0	103	No correction required
	Cd	0.99997	5	Yes	0.00854	0.0108	6.4716	3.0	97.3	No correction required
	Pb	0.99977	5	Yes	0.0157	0.0471	3.666	13.0	106	Corrected by spectral separation and background correction
	Co	0.99996	5	Yes	0.00104	0.0031	6.0485	13.0	97.6	No correction required
	Ni	0.99971	5	Yes	0.00391	0.0117	1.4346	11.0	103	Corrected by spectral separation and background correction
	Sr	0.99996	5	Yes	0.00848	0.0254	1.4404	13.0	97.9	No correction required
	V	0.99999	5	Yes	0.00103	0.0031	1.2214	8.0	95.9	No correction required
	Fe	0.99878	5	Yes	0.00219	0.08213	5.9312	11.0	98.7	Background corrected by background shift;

									evaluated with blank matrix solution
Mn	0.99991	5	Yes	0.00186	0.00559	2.9967	8.0	99.3	No correction required
Cu	0.99993	5	Yes	0.00097	0.00291	1.6857	4.0	98.8	No correction required
Cr	0.99991	5	Yes	0.00097	0.00291	2.7829	6.0	106	No correction required
As	0.99958	5	Yes	0.02591	0.0777	6.7358	11.0	101	No correction required
Hg	0.99978	5	Yes	0.0157	0.04735	5.8359	13.0	105	Corrected by spectral separation and background correction
Li	0.99987	5	Yes	0.00362	0.0108	1.1774	8.0	94.4	Corrected by spectral separation and background correction

Table S5. Transfer factor (TF) mean and standard error of the mean (SE)

Vegetable	Mean TF (Li)	SE (Li)	Mean TF (Sr)	SE (Sr)	Mean TF (Zn)	SE (Zn)	Mean TF (Cu)	SE (Cu)	Mean TF (Pb)	SE (Pb)	Mean TF (Cd)	SE (Cd)
Cucumbers	0.00036	0.00004	0.01026	0.00114	0.11300	0.00806	0.03367	0.00160	0.00201	0.00017	0.00067	0.00007
Garlic	0.00047	0.00003	0.02100	0.00259	0.12658	0.00696	0.05442	0.00392	0.00494	0.00067	0.00120	0.00018
Onion	0.00035	0.00004	0.01758	0.00196	0.10777	0.00511	0.04369	0.00378	0.00305	0.00037	0.00085	0.00006
Salad	0.00040	0.00004	0.01625	0.00114	0.12028	0.00662	0.05315	0.00376	0.00277	0.00028	0.00091	0.00005
Zucchini	0.00041	0.00004	0.01817	0.00196	0.11797	0.00619	0.04077	0.00351	0.00281	0.00026	0.00086	0.00006

Table S6. Descriptive statistics and outlier detection for statistically significant metal–plant–culture combinations

Plant	Metal	Culture	n (no. of samples)	Min	Q1	Median	Q3	Max	Outliers
garlic	Co	Greenhouse	19	0.001	0.002	0.0035	0.01875	0.367	0.064<br>0.354<br>0.367
garlic	Co	Field	19	0.001	0.00675	0.0205	0.06125	0.647	0.647<br>0.214
garlic	Mn	Greenhouse	19	1.501	3.39	6.1415	10.59	20.01	None
garlic	Mn	Field	19	6.36	12.44	14.7875	18.16	23.50	None
cucumber	Zn	Greenhouse	19	11.01	13.13	15.23	17.87	30.20	30.205
cucumber	Zn	Field	19	15.14	17.09	18.71	20.44	35.12	35.125
cucumber	Cu	Greenhouse	19	0.137	4.17	6.22	7.15	32.14	32.140
cucumber	Cu	Field	19	0.236	6.27	7.22	8.91	121	121.0<br>0.236
zucchini	Cd	Greenhouse	19	0.000148	0.001	0.002	0.00495	0.011	0.011
zucchini	Cd	Field	19	0.000148	0.0035	0.007	0.009	0.021	0.021
zucchini	V	Greenhouse	19	0.005	0.0125	0.021	0.072	0.988	0.310<br>0.988
zucchini	V	Field	19	0.008	0.04	0.054	0.084	1.001	0.236<br>1.001

Table S7. Greenhouse vs. field cultivation: p-values, effect sizes, and confidence intervals

Vegetable	Metal	Test	p value	Effect size	Effect metric	n greenhouse	n field	CI95 low	CI95 high	p value FDR
cucumber	Zn	Mann-Whitney U	0.007233	0.512465	Rank-biserial r	19	19	-5.82	-0.864	0.10126
cucumber	Cd	Mann-Whitney U	0.813852	-0.047091	Rank-biserial r	19	19	-0.009	0.013	0.90952
cucumber	Pb	Mann-Whitney U	0.599232	-0.102493	Rank-biserial r	19	19	-0.122	0.145	0.90952
cucumber	Co	Mann-Whitney U	0.113873	0.301939	Rank-biserial r	19	19	-0.038	0.008	0.53141
cucumber	Ni	Mann-Whitney U	0.988353	0.00554	Rank-biserial r	19	19	-0.537	0.42	0.98835
cucumber	Sr	t-test (Welch)	0.844557	-0.064074	Cohen_d	19	19	-2.409	2.004	0.90952
cucumber	V	Mann-Whitney U	0.725956	-0.069252	Rank-biserial r	19	19	-0.017	0.041	0.90952
cucumber	Fe	Mann-Whitney U	0.715146	-0.072022	Rank-biserial r	19	19	-4.05	7.622	0.90952
cucumber	Mn	Mann-Whitney U	0.559248	0.113573	Rank-biserial r	19	19	-5.59	4.300	0.90952
cucumber	Cu	Mann-Whitney U	0.040979	0.390582	Rank-biserial r	19	19	-3.763	0.110	0.28685
cucumber	Cr	Mann-Whitney U	0.388999	-0.166205	Rank-biserial r	19	19	-0.326	0.566	0.90952
cucumber	As	Mann-Whitney U	0.46432	-0.141274	Rank-biserial r	19	19	-0.113	0.125	0.90952
cucumber	Hg	Mann-Whitney U	0.537808	0.119114	Rank-biserial r	19	19	-0.229	0.122	0.90952
cucumber	Li	t-test (Welch)	0.458902	-0.243036	Cohen_d	19	19	-0.266	0.127	0.90952
garlic	Zn	Mann-Whitney U	0.384267	0.17284	Rank-biserial r	18	18	-6.053	2.887	0.53797
garlic	Cd	Mann-Whitney U	0.709313	-0.074074	Rank-biserial r	18	18	-0.047	0.049	0.82753
garlic	Pb	Mann-Whitney U	0.318858	-0.197531	Rank-biserial r	18	18	-0.421	0.459	0.53797
garlic	Co	Mann-Whitney U	0.033622	0.416667	Rank-biserial r	18	18	-0.054	-0.002	0.19586
garlic	Ni	Mann-Whitney U	0.090519	-0.333333	Rank-biserial r	18	18	-0.144	1.666	0.22565
garlic	Sr	t-test (Welch)	0.041971	-0.706699	Cohen_d	18	18	-9.0605	-0.575	0.19586
garlic	V	Mann-Whitney U	0.073844	-0.351852	Rank-biserial r	18	18	-0.009	1.155	0.22565
garlic	Fe	Mann-Whitney U	0.096708	-0.32716	Rank-biserial r	18	18	-0.844	18.459	0.22565
garlic	Mn	Mann-Whitney U	0.001118	0.638889	Rank-biserial r	18	18	-12.921	-4.339	0.01565
garlic	Cu	Mann-Whitney U	0.53724	0.123457	Rank-biserial r	18	18	-1.955	1.277	0.68376
garlic	Cr	Mann-Whitney U	0.384267	-0.17284	Rank-biserial r	18	18	-0.815	3.005	0.53797
garlic	As	Mann-Whitney U	0.222564	0.237654	Rank-biserial r	18	18	-0.144	0.035	0.44513
garlic	Hg	Mann-Whitney U	0.931322	0.018519	Rank-biserial r	18	18	-0.0008	0.0008	1.00000
garlic	Li	Mann-Whitney U	1	-0.003086	Rank-biserial r	18	18	-0.2600	0.226	1.00000
lettuce	Zn	Mann-Whitney U	0.804004	-0.049861	Rank-biserial r	19	19	-13.908	17.058	0.95344
lettuce	Cd	Mann-Whitney U	0.106901	-0.299169	Rank-biserial r	19	19	-0.009	0.189	0.85023
lettuce	Pb	Mann-Whitney U	0.815328	0.047091	Rank-biserial r	19	19	-0.806	0.666	0.95344
lettuce	Co	Mann-Whitney U	0.714832	0.072022	Rank-biserial r	19	19	-0.262	0.191	0.95344
lettuce	Ni	Mann-Whitney U	0.860947	-0.036011	Rank-biserial r	19	19	-3.362	3.700	0.95344
lettuce	Sr	Mann-Whitney U	0.661443	0.085873	Rank-biserial r	19	19	-32.26	28.376	0.95344
lettuce	V	Mann-Whitney U	0.559291	-0.113573	Rank-biserial r	19	19	-5.277	6.949	0.95344
lettuce	Fe	Mann-Whitney U	0.930207	0.019391	Rank-biserial r	19	19	-202.792	193.670	0.95344
lettuce	Mn	Mann-Whitney U	0.953438	0.01385	Rank-biserial r	19	19	-21.744	24.530	0.95344
lettuce	Cu	Mann-Whitney U	0.79273	0.052632	Rank-biserial r	19	19	-6.125	4.890	0.95344

lettuce	Cr	Mann-Whitney U	0.704293	-0.074792	Rank-biserial r	19	19	-12.204	12.875	0.95344
lettuce	As	Mann-Whitney U	0.883866	0.030471	Rank-biserial r	19	19	-0.115	0.116	0.95344
lettuce	Hg	Mann-Whitney U	0.121461	-0.274238	Rank-biserial r	19	19	-0.0002	0.0049	0.850
lettuce	Li	Mann-Whitney U	0.804004	0.049861	Rank-biserial r	19	19	-0.366	0.079	0.95344
onion	Zn	Mann-Whitney U	0.5791	-0.108033	Rank-biserial r	19	19	-4.630	6.580	0.72295
onion	Cd	Mann-Whitney U	0.871944	-0.033241	Rank-biserial r	19	19	-0.014	0.026	0.87194
onion	Pb	Mann-Whitney U	0.156701	-0.271468	Rank-biserial r	19	19	-0.159	0.443	0.36564
onion	Co	Mann-Whitney U	0.074709	-0.34072	Rank-biserial r	19	19	-0.008	0.081	0.34399
onion	Ni	Mann-Whitney U	0.070207	-0.34626	Rank-biserial r	19	19	-0.069	0.920	0.34399
onion	Sr	t-test (Welch)	0.122854	0.513476	Cohen_d	19	19	-1.149	12.655	0.34399
onion	V	Mann-Whitney U	0.065831	-0.351801	Rank-biserial r	19	19	0.040	1.997	0.34399
onion	Fe	Mann-Whitney U	0.114907	-0.301939	Rank-biserial r	19	19	-27.590	62.757	0.34399
onion	Mn	Mann-Whitney U	0.267257	-0.213296	Rank-biserial r	19	19	-1.811	6.012	0.41573
onion	Cu	Mann-Whitney U	0.619675	-0.096953	Rank-biserial r	19	19	-0.896	1.290	0.72295
onion	Cr	Mann-Whitney U	0.260962	-0.216066	Rank-biserial r	19	19	-0.724	1.760	0.41573
onion	As	Mann-Whitney U	0.183434	-0.254848	Rank-biserial r	19	19	-0.039	0.114	0.36687
onion	Hg	Mann-Whitney U	0.390739	0.163435	Rank-biserial r	19	19	-0.009	0.009	0.54703
onion	Li	Mann-Whitney U	0.7591	0.060942	Rank-biserial r	19	19	-0.253	0.218	0.81749
zucchini	Zn	t-test (Welch)	0.414731	-0.267751	Cohen_d	19	19	-5.243	1.990	0.58062
zucchini	Cd	Mann-Whitney U	0.003501	0.554017	Rank-biserial r	19	19	-0.008	-0.001	0.04902
zucchini	Pb	Mann-Whitney U	0.38109	0.168975	Rank-biserial r	19	19	-0.566	0.407	0.58062
zucchini	Co	Mann-Whitney U	0.161064	0.268698	Rank-biserial r	19	19	-0.286	0.101	0.33909
zucchini	Ni	Mann-Whitney U	0.114887	0.301939	Rank-biserial r	19	19	-1.641	0.482	0.32168
zucchini	Sr	t-test (Welch)	0.631776	-0.156821	Cohen_d	19	19	-3.049	1.776	0.63177
zucchini	V	Mann-Whitney U	0.027459	0.421053	Rank-biserial r	19	19	-0.060	0.017	0.19221
zucchini	Fe	Mann-Whitney U	0.619656	0.096953	Rank-biserial r	19	19	-6.662	4.814	0.63177
zucchini	Mn	t-test (Welch)	0.352007	-0.305975	Cohen_d	19	19	-4.102	1.369	0.58062
zucchini	Cu	t-test (Welch)	0.521084	-0.210246	Cohen_d	19	19	-3.055	1.529	0.60793
zucchini	Cr	Mann-Whitney U	0.079795	0.33518	Rank-biserial r	19	19	-0.404	-0.001	0.27928
zucchini	As	Mann-Whitney U	0.067041	0.34903	Rank-biserial r	19	19	-0.051	0.012	0.27928
zucchini	Hg	Mann-Whitney U	0.169548	0.263158	Rank-biserial r	19	19	-0.287	0.124	0.33909
zucchini	Li	Mann-Whitney U	0.483483	0.135734	Rank-biserial r	19	19	-0.398	0.198	0.60793

Table S8. Descriptive statistics (mean, standard deviation, median, quartiles and IQR) for metal concentrations in different vegetables under field and greenhouse cultivation conditions

Vegetable	CultivationType	Metal	n	mean	sd	median	q1	q3	iqr
cucumber	Field	As	19	0.1652	0.3288	0.01	0.003	0.1765	0.1735
cucumber	Field	Cd	19	0.0697	0.2303	0.01	0.0022	0.0125	0.0103
cucumber	Field	Co	19	0.1042	0.151	0.051	0.031	0.07	0.039
cucumber	Field	Cr	19	0.9163	2.065	0.336	0.0405	0.422	0.3815
cucumber	Field	Cu	19	13.1	26.2	7.22	6.2745	8.91	2.6355
cucumber	Field	Fe	19	23.1	13.1	21.22	19.6	23.625	3.9845
cucumber	Field	Hg	19	0.1384	0.1354	0.123	0.0024	0.257	0.2546
cucumber	Field	Li	19	0.5781	0.2819	0.549	0.3715	0.7685	0.397
cucumber	Field	Mn	19	12.7	3.932	11.72	10.2	13.48	3.2905
cucumber	Field	Ni	19	2.3622	4.8909	1.037	0.736	1.61	0.874
cucumber	Field	Pb	19	0.3393	0.2792	0.226	0.1765	0.434	0.2575
cucumber	Field	Sr	19	11.836	3.7582	11.9	9.5725	13.94	4.3675
cucumber	Field	V	19	0.0751	0.0552	0.061	0.0495	0.0715	0.022
cucumber	Field	Zn	19	19.4	4.4085	18.7	17.1	20.4	3.3565
cucumber	Greenhouse	As	19	0.248	0.3913	0.039	0.004	0.4135	0.4094
cucumber	Greenhouse	Cd	19	0.0861	0.2414	0.01	0.001	0.02	0.019
cucumber	Greenhouse	Co	19	0.0697	0.1173	0.032	0.02	0.049	0.029
cucumber	Greenhouse	Cr	19	1.2167	2.6524	0.41	0.035	0.665	0.63
cucumber	Greenhouse	Cu	19	6.8176	6.527	6.22	4.17	7.155	2.985
cucumber	Greenhouse	Fe	19	28.7	24.2	22.6	16.6	30.1	13.452
cucumber	Greenhouse	Hg	19	0.1	0.1161	0.017	0.0018	0.18	0.1782
cucumber	Greenhouse	Li	19	0.5006	0.3521	0.43	0.2625	0.65	0.3875
cucumber	Greenhouse	Mn	19	12.3	5.9164	9.6	7.28	16.9	9.65
cucumber	Greenhouse	Ni	19	1.853	3.3174	1.017	0.905	1.5445	0.6395
cucumber	Greenhouse	Pb	19	0.3646	0.2621	0.29	0.184	0.5085	0.3245
cucumber	Greenhouse	Sr	19	11.6	3.5983	10.7	8.97	14.565	5.595
cucumber	Greenhouse	V	19	0.1526	0.2721	0.064	0.05	0.1015	0.0515
cucumber	Greenhouse	Zn	19	16.3	4.5643	15.2	13.1	17.9	4.7315
garlic	Field	As	18	0.1872	0.33	0.02	0.0031	0.1925	0.1894
garlic	Field	Cd	18	0.0415	0.0638	0.0122	0.001	0.0507	0.0497
garlic	Field	Co	18	0.072	0.152	0.0205	0.0068	0.0612	0.0545
garlic	Field	Cr	18	4.0835	3.9394	2.659	2.1258	3.8625	1.7367
garlic	Field	Cu	18	5.523	13.2442	2.7085	1.1288	3.5638	2.435
garlic	Field	Fe	18	46.1	17.3	43.8	38.596	49.2	10.6
garlic	Field	Hg	18	0.0075	0.0249	0.0014	0.001	0.0018	0.0008
garlic	Field	Li	18	16.5	50.1	0.7045	0.4462	0.9058	0.4595
garlic	Field	Mn	18	14.9	4.9181	14.8	12.4	18.2	5.7227
garlic	Field	Ni	18	1.6973	1.3396	1.461	1.0312	2.0708	1.0395
garlic	Field	Pb	18	1.5033	2.703	0.191	0.1023	0.8998	0.7975
garlic	Field	Sr	18	20.6	7.7993	20.3	13.5	26.3	12.8
garlic	Field	V	18	1.5926	0.8509	1.2985	1.0252	1.8178	0.7925
garlic	Field	Zn	18	14.5	8.7102	12.2	9.0225	15.5	6.5
garlic	Greenhouse	As	18	0.1006	0.2508	0.0024	0.001	0.051	0.05
garlic	Greenhouse	Cd	18	0.075	0.1762	0.0175	0.001	0.0632	0.0622
garlic	Greenhouse	Co	18	0.0488	0.1144	0.0035	0.002	0.0188	0.0168
garlic	Greenhouse	Cr	18	4.3791	2.766	3.8665	2.1525	5.582	3.4295
garlic	Greenhouse	Cu	18	2.4016	1.7346	2.15	1.0412	2.9618	1.9205
garlic	Greenhouse	Fe	18	54.5	19.7	54.6	43.1	61.4	18.4
garlic	Greenhouse	Hg	18	0.0027	0.0038	0.001	0.001	0.0019	0.0009
garlic	Greenhouse	Li	18	17.4	53.5	0.684	0.4732	0.8732	0.4
garlic	Greenhouse	Mn	18	7.9942	5.8088	6.1415	3.3865	10.6	7.2045
garlic	Greenhouse	Ni	18	2.2277	1.3319	2.093	1.306	3.1093	1.8033
garlic	Greenhouse	Pb	18	1.5027	2.7486	0.3405	0.1315	0.6192	0.4878
garlic	Greenhouse	Sr	18	15.8	5.8414	14.9	10.9	17.7	6.6965
garlic	Greenhouse	V	18	2.0183	0.8543	1.97	1.46	2.4025	0.9425
garlic	Greenhouse	Zn	18	12.7	8.1724	9.724	8.0908	14.3	6.211
lettuce	Field	As	19	0.242	0.3648	0.115	0.0115	0.251	0.2395
lettuce	Field	Cd	19	0.0459	0.0876	0.0027	0.001	0.0255	0.0245
lettuce	Field	Co	19	0.3695	0.5788	0.129	0.0045	0.409	0.4045
lettuce	Field	Cr	19	9.4381	9.8663	3.609	2.3745	17.9	15.6
lettuce	Field	Cu	19	9.9534	13.8	4.63	2.2485	9.829	7.5805

lettuce	Field	Fe	19	274	235	212	98.9	408	310
lettuce	Field	Hg	19	0.0099	0.0252	0.001	0.001	0.0031	0.002
lettuce	Field	Li	19	0.8049	1.3741	0.276	0.2055	0.6485	0.443
lettuce	Field	Mn	19	82.8	36.9	90.1	75.6	111	35.6
lettuce	Field	Ni	19	3.4282	3.1692	2.128	1.16	5.655	4.495
lettuce	Field	Pb	19	2.0947	4.2494	1.009	0.426	1.8055	1.3795
lettuce	Field	Sr	19	43.9	30.6	38.6	18.3	60.84	42.6
lettuce	Field	V	19	4.5673	4.531	2.348	1.022	7.7115	6.6895
lettuce	Field	Zn	19	23.8	15.7	18.3	13.2	34.6	21.3
lettuce	Greenhouse	As	19	0.1787	0.288	0.098	0.013	0.21	0.197
lettuce	Greenhouse	Cd	19	0.2291	0.4066	0.026	0.001	0.235	0.234
lettuce	Greenhouse	Co	19	0.1942	0.256	0.14	0.0045	0.274	0.2695
lettuce	Greenhouse	Cr	19	10.5	11.4	5.44	2.9365	17.01	14.1
lettuce	Greenhouse	Cu	19	9.3211	15.1	5.036	2.6745	9.0385	6.364
lettuce	Greenhouse	Fe	19	310	356	188	95.9	400	305
lettuce	Greenhouse	Hg	19	0.0271	0.0652	0.0018	0.001	0.0063	0.0053
lettuce	Greenhouse	Li	19	0.6798	1.1836	0.239	0.1965	0.444	0.2475
lettuce	Greenhouse	Mn	19	86.2	39.6	98.6	75.7	109	33.1
lettuce	Greenhouse	Ni	19	3.8134	3.778	2.308	1.1485	5.385	4.2365
lettuce	Greenhouse	Pb	19	1.0927	0.8784	0.85	0.5165	1.387	0.8705
lettuce	Greenhouse	Sr	19	42.3	33.5	36.1	14.7	53.7	38.9
lettuce	Greenhouse	V	19	5.0787	4.3468	2.739	1.2535	8.585	7.3315
lettuce	Greenhouse	Zn	19	26.6	20.4	20.1	13.276	37.1	23.8
onion	Field	As	19	0.044	0.0544	0.011	0.003	0.085	0.082
onion	Field	Cd	19	0.0416	0.033	0.04	0.0125	0.052	0.0395
onion	Field	Co	19	0.0703	0.2279	0.009	0.0045	0.035	0.0305
onion	Field	Cr	19	1.5451	1.4383	1.26	0.25	2.3615	2.1115
onion	Field	Cu	19	4.4934	5.2018	3.129	2.315	4.0685	1.7535
onion	Field	Fe	19	32.6	28.4	14.8	9.7965	46.5	36.7
onion	Field	Hg	19	0.0504	0.1058	0.0034	0.0018	0.0116	0.0099
onion	Field	Li	19	2.9604	10.8	0.375	0.1515	0.411	0.2595
onion	Field	Mn	19	13.9	7.7857	13.9	9.241	16.9	7.691
onion	Field	Ni	19	1.1912	0.6567	1.28	0.9005	1.6295	0.729
onion	Field	Pb	19	0.3007	0.3315	0.19	0.1065	0.419	0.3125
onion	Field	Sr	19	13.0	9.3365	14.1	3.836	17.1	13.3
onion	Field	V	19	1.1095	1.2811	1.12	0.0745	1.385	1.3105
onion	Field	Zn	19	19.3	18.2	14.4	9.71	18.4	8.69
onion	Greenhouse	As	19	0.0674	0.061	0.07	0.005	0.12	0.115
onion	Greenhouse	Cd	19	0.0534	0.0715	0.039	0.0322	0.05	0.0178
onion	Greenhouse	Co	19	0.1021	0.1963	0.05	0.009	0.101	0.092
onion	Greenhouse	Cr	19	3.8372	6.2287	1.73	0.713	2.875	2.162
onion	Greenhouse	Cu	19	4.3755	3.9073	3.412	2.3615	4.63	2.2685
onion	Greenhouse	Fe	19	58.4	51.3	49.8	13.2	87.3	74.1
onion	Greenhouse	Hg	19	0.0319	0.0789	0.0018	0.0017	0.016	0.0143
onion	Greenhouse	Li	19	2.6759	9.2054	0.312	0.124	0.6215	0.4975
onion	Greenhouse	Mn	19	17.1	10.7	15.2	13.8	18.6	4.805
onion	Greenhouse	Ni	19	1.8849	1.295	1.611	1.2795	2.22	0.9405
onion	Greenhouse	Pb	19	0.4744	0.4552	0.27	0.125	0.651	0.526
onion	Greenhouse	Sr	19	18.6	12.2	18.2	13.1	21.8	8.6825
onion	Greenhouse	V	19	1.8309	1.4987	1.48	1.1905	2.334	1.1435
onion	Greenhouse	Zn	19	20.8	19.4	12.5	11.1	18.9	7.755
zucchini	Field	As	19	0.2163	0.7107	0.009	0.007	0.0585	0.0515
zucchini	Field	Cd	19	0.0073	0.0051	0.007	0.0035	0.009	0.0055
zucchini	Field	Co	19	0.3346	0.2843	0.307	0.099	0.4595	0.3605
zucchini	Field	Cr	19	1.0404	1.8051	0.505	0.32	0.6935	0.3735
zucchini	Field	Cu	19	7.7952	3.8858	8.014	5.2145	9.7255	4.511
zucchini	Field	Fe	19	19.8	15.4	17.2	11.3	22.1	10.8
zucchini	Field	Hg	19	0.1822	0.163	0.201	0.002	0.351	0.349
zucchini	Field	Li	19	3.6383	9.3539	0.414	0.304	0.7745	0.4705
zucchini	Field	Mn	19	12.1	4.6895	13.0	9.701	15.6	5.8545
zucchini	Field	Ni	19	3.1764	3.283	2.008	1.9175	3.554	1.6365
zucchini	Field	Pb	19	0.6725	0.9071	0.213	0.131	0.749	0.618
zucchini	Field	Sr	19	10.1	3.8138	9.258	6.6995	13.1	6.3525
zucchini	Field	V	19	0.1135	0.2203	0.054	0.04	0.084	0.044

zucchini	Field	Zn	19	17.3	6.2022	18.1	11.5	20.7	9.161
zucchini	Greenhouse	As	19	0.1545	0.5386	0.003	0.0025	0.0255	0.023
zucchini	Greenhouse	Cd	19	0.0032	0.003	0.002	0.001	0.0049	0.0039
zucchini	Greenhouse	Co	19	0.2182	0.2288	0.135	0.0765	0.3075	0.231
zucchini	Greenhouse	Cr	19	0.8178	1.6322	0.302	0.2055	0.433	0.2275
zucchini	Greenhouse	Cu	19	6.9881	3.7914	7.143	4.7375	9.161	4.4235
zucchini	Greenhouse	Fe	19	18.1	12.6	16.67	10.1	21.6	11.5
zucchini	Greenhouse	Hg	19	0.1255	0.1181	0.114	0.0018	0.2385	0.2367
zucchini	Greenhouse	Li	19	3.2646	8.5882	0.465	0.1755	0.6005	0.425
zucchini	Greenhouse	Mn	19	10.7	4.1866	11.8	8.5145	13.4	4.9325
zucchini	Greenhouse	Ni	19	2.3874	2.8892	1.512	1.0505	2.56	1.5095
zucchini	Greenhouse	Pb	19	0.4606	0.5804	0.162	0.1445	0.6225	0.478
zucchini	Greenhouse	Sr	19	9.4921	3.9906	8.23	6.5735	12.7	6.157
zucchini	Greenhouse	V	19	0.0958	0.2267	0.021	0.0125	0.072	0.0595
zucchini	Greenhouse	Zn	19	15.	5.5161	17.1	9.734	18.9	9.231

Table S9. Elements and weight of Cluster 1

**ID**	**Weight**
Metals/Elements	
As	60.4
Cd	24.6
Co	59.9
Cu	2673
Hg	25.7
Ni	905
Sr	7420
Zn	7034
Locations (Counties)	
bacau	1479
bihor	952
calarasi	1809
constanta	736
dambovita	1508
giurgiu	1815
ialomita	1956
tulcea	799
Species/Crops	
cucumber	3178
onion	4235
zucchini	2793

Table S10. Elements and weight of Cluster 2

**ID**	Cr	Li	Pb	dolj	garlic	ilfov	olt
**Weight**	1420	1797	329	1853	4510	2417	1872

Table S11. Elements and weight of Clusters 3, 4 and 5

**Cluster 3**		**Cluster 4**		**Cluster 5**	
ID	Weight	ID	Weight	ID	Weight
Fe	32651.6	arges	625	Mn	10249
buzau	2339.16	bucuresti	1798	braila	1028
lettuce	18108.1	vrancea	2374	galati	1174
valcea	3768.57	V	2416	mehedinti	732

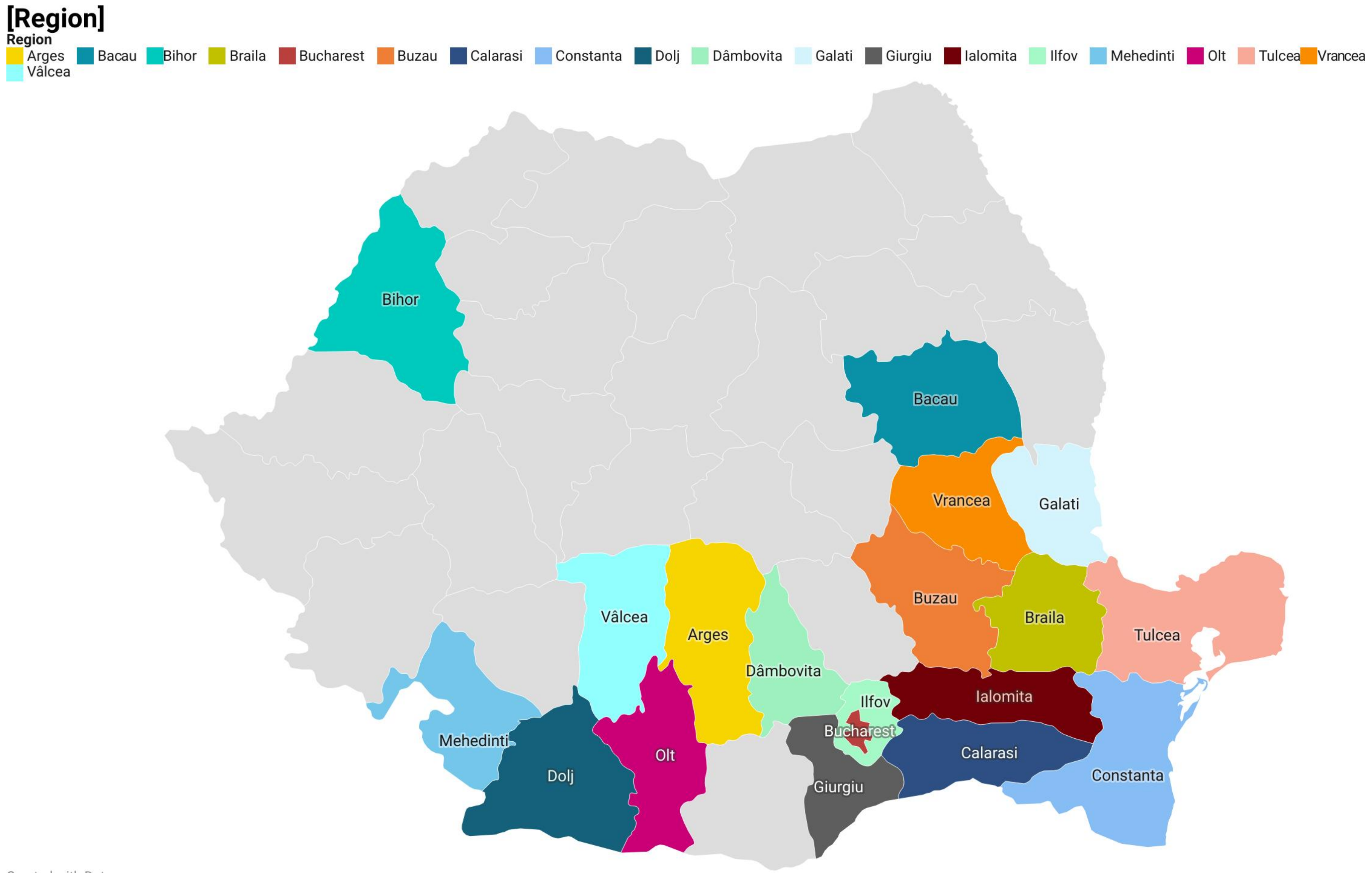

Figure S1. Sampling regions within Romania

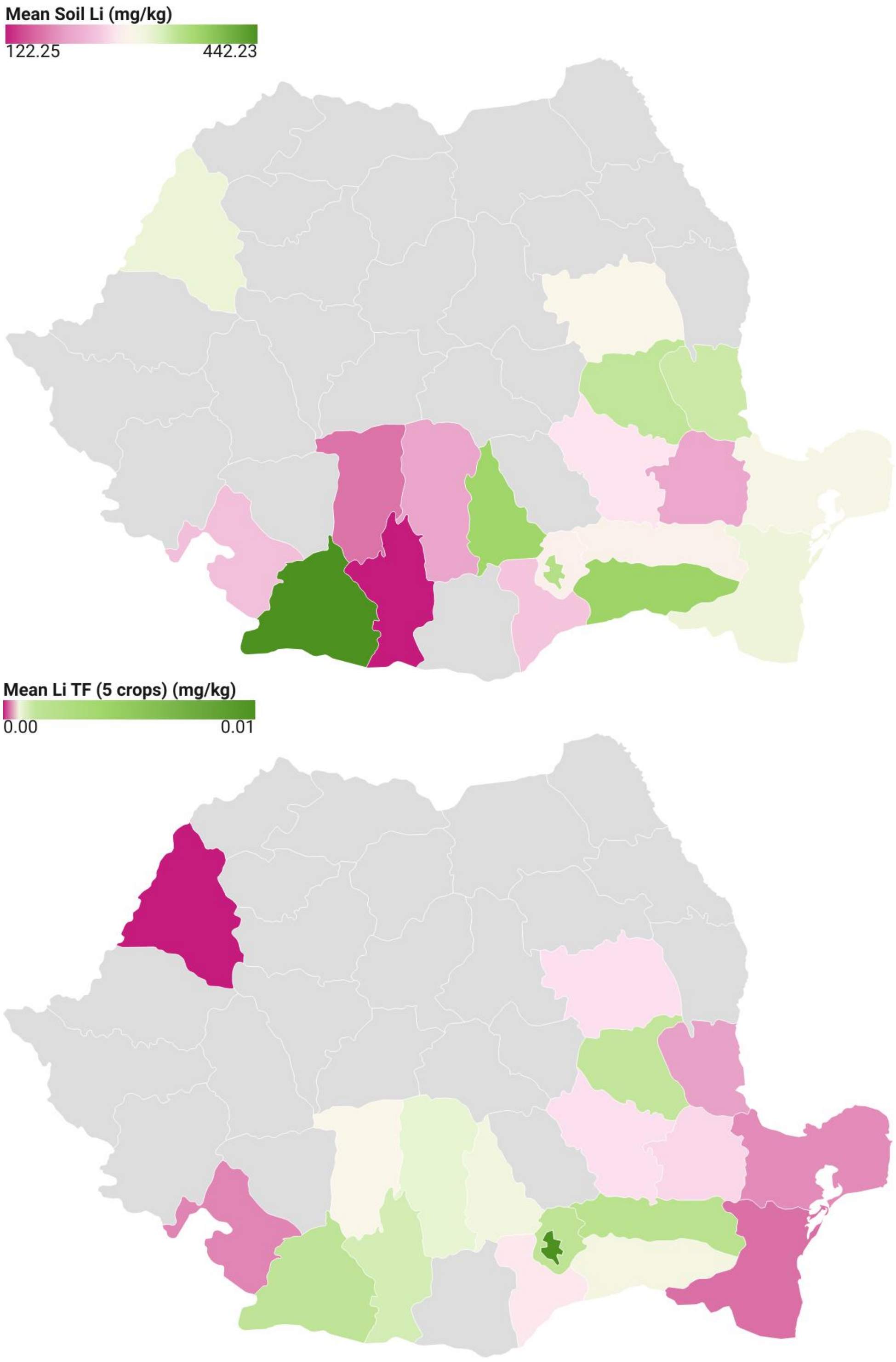

Figure S2. Lithium concentration in soil and median uptake
