# Supplementary material for: From Soil to Plate: Lithium and Other Trace Metals Uptake in Vegetables Under Variable Soil Conditions
Source: Toxics. 2025 Nov 5;13(11):956. doi: 10.3390/toxics13110956 (PMC12656363; doi:10.3390/toxics13110956)
Supplement: Supplementary file 1 [file toxics-13-00956-s001.zip › toxics-3937158-supplementary-11-3 - review/Supplementary Material_2.pdf]

Sample	Region	Zn	Cd	Pb	Co	Ni	Sr	V	Fe	Mn	Cu	Cr	As	Hg	Li	Sample Type	Cultivation Type	Vegetable Type
		mg/kg																
cucumber	arges	21.450000	0.015000	0.196000	0.047000	1.118000	12.850000	0.051000	20.190000	11.330000	6.931000	0.434000	0.003000	0.246000	0.514000	vegetable	field	aerial
cucumber	arges	13.980000	0.010000	0.270000	0.028000	1.769000	12.840000	0.050000	31.440000	7.330000	4.310000	0.410000	0.040000	0.190000	0.390000	vegetable	greenhouse	aerial
cucumber	bacau	17.780000	0.017000	0.230000	0.030000	1.550000	8.290000	0.040000	25.990000	11.720000	6.410000	0.580000	0.003000	0.330000	0.820000	vegetable	field	aerial
cucumber	bacau	12.200000	0.030000	0.760000	0.030000	1.320000	14.810000	0.050000	25.670000	7.620000	4.080000	0.690000	0.010000	0.190000	0.430000	vegetable	greenhouse	aerial
cucumber	bihor	21.078000	0.169000	0.226000	0.001000	0.021000	19.220000	0.098000	11.460000	19.320000	8.560000	0.012000	1.201000	0.124000	0.345000	vegetable	field	aerial
cucumber	bihor	20.056000	1.021000	0.229000	0.001000	0.017000	18.340000	0.102000	10.531000	21.220000	6.220000	0.024000	1.213000	0.012000	0.031200	vegetable	greenhouse	aerial
cucumber	braila	16.236000	0.001000	0.222000	0.068000	1.216000	8.660000	0.048000	21.078000	22.136000	8.123000	0.041000	0.001000	0.001000	0.322000	vegetable	field	aerial
cucumber	braila	16.101000	0.001000	0.032000	0.012000	1.017000	9.620000	0.037000	19.780000	20.149000	6.520000	0.045000	0.001000	0.001000	0.365000	vegetable	greenhouse	aerial
cucumber	bucuresti	17.152000	0.010000	0.412000	0.051000	1.670000	6.230000	0.060000	26.370000	13.720000	7.170000	0.598000	0.123000	0.392000	0.920000	vegetable	field	aerial
cucumber	bucuresti	13.210000	0.001000	0.860000	0.048000	1.010000	10.120000	0.051000	25.360000	7.230000	7.001000	0.632000	0.001000	0.320000	0.670000	vegetable	greenhouse	aerial
cucumber	buzau	15.920000	0.032000	0.120000	0.030000	3.940000	12.950000	0.061000	56.320000	9.120000	7.640000	7.230000	0.230000	0.183000	0.840000	vegetable	field	aerial
cucumber	buzau	12.080000	0.020000	0.290000	0.050000	2.020000	16.210000	0.110000	57.820000	7.980000	5.120000	8.250000	0.740000	0.170000	0.630000	vegetable	greenhouse	aerial
cucumber	calarasi	19.812000	0.002000	0.211000	0.032000	1.210000	12.180000	0.056000	21.370000	13.240000	10.480000	0.380000	0.001000	0.123000	0.678000	vegetable	field	aerial
cucumber	calarasi	22.380000	0.005000	0.152000	0.078000	2.107000	14.560000	0.068000	109.200000	22.360000	32.140000	0.890000	0.012000	0.012000	1.312000	vegetable	greenhouse	aerial
cucumber	constanta	15.291000	0.010000	0.148000	0.041000	1.013000	10.122000	0.068000	20.130000	10.256000	7.220000	0.010000	0.037000	0.001000	0.612000	vegetable	field	aerial
cucumber	constanta	15.232000	0.098000	0.216000	0.046000	1.221000	7.330000	0.062000	17.200000	14.600000	7.120000	0.021000	0.039000	0.001000	0.452000	vegetable	greenhouse	aerial
cucumber	dambovita	18.210000	0.001000	0.231000	0.030000	0.415000	15.360000	0.052000	22.460000	10.123000	121.000000	0.410000	0.310000	0.032000	0.717000	vegetable	field	aerial
cucumber	dambovita	15.370000	0.001000	0.105000	0.032000	1.241000	14.210000	0.064000	20.130000	18.230000	7.560000	0.387000	0.930000	0.017000	1.201000	vegetable	greenhouse	aerial
cucumber	dolj	35.125000	0.002400	1.002000	0.128000	1.037000	9.023000	0.236000	8.789000	6.145000	4.025000	0.033000	0.005000	0.003000	0.125000	vegetable	field	aerial
cucumber	dolj	30.205000	0.001900	0.657000	0.009700	0.010000	8.123000	0.101000	7.023000	5.014000	4.010000	0.025000	0.004100	0.001766	0.085000	vegetable	greenhouse	aerial
cucumber	galati	22.660000	0.001000	1.012000	0.560000	2.630000	10.231000	0.038000	21.220000	18.219000	9.260000	0.066000	0.037000	0.001000	0.421000	vegetable	field	aerial
cucumber	galati	20.312000	0.001000	0.360000	0.510000	2.790000	10.100000	0.041000	18.690000	20.660000	7.213000	0.068000	0.007000	0.001000	0.305000	vegetable	greenhouse	aerial
cucumber	giurgiu	19.790000	0.010000	0.170000	0.061000	0.650000	14.930000	0.064000	23.830000	13.240000	5.932000	0.338000	0.003000	0.308000	0.549000	vegetable	field	aerial
cucumber	giurgiu	14.340000	0.020000	0.220000	0.040000	0.900000	14.570000	0.070000	28.700000	9.600000	4.110000	0.580000	0.130000	0.140000	0.740000	vegetable	greenhouse	aerial
cucumber	ialomita	15.136000	1.008000	0.456000	0.455000	22.214000	17.197000	0.103000	19.158000	8.252000	11.034000	0.025000	0.888000	0.005400	0.315000	vegetable	field	aerial
cucumber	ialomita	13.057000	0.367000	0.324000	0.218000	15.222000	15.037000	0.670000	16.036000	7.014000	10.004000	0.022000	0.697000	0.003900	0.214000	vegetable	greenhouse	aerial
cucumber	ilfov	19.800000	0.010000	0.243000	0.052000	0.801000	11.920000	0.063000	21.250000	11.460000	6.139000	0.388000	0.003000	0.285000	0.954000	vegetable	field	aerial
cucumber	ilfov	14.450000	0.020000	0.330000	0.030000	1.260000	13.410000	0.070000	36.730000	8.370000	4.360000	0.640000	0.050000	0.250000	0.450000	vegetable	greenhouse	aerial
cucumber	mehedinti	17.320000	0.007000	0.066000	0.001000	1.002000	11.221000	0.012000	12.368000	10.122000	7.220000	0.056000	0.023000	0.001000	0.398000	vegetable	field	aerial
cucumber	mehedinti	12.890000	0.017000	0.114000	0.001000	1.013000	10.698000	0.011000	11.590000	15.630000	6.350000	0.062000	0.004000	0.001000	0.349000	vegetable	greenhouse	aerial
cucumber	olt	17.025000	0.000148	0.555000	0.219000	0.412000	12.190000	0.202000	6.625000	16.158000	0.236000	0.040000	0.001480	0.001766	0.213000	vegetable	field	aerial
cucumber	olt	16.357000	0.000148	0.315000	0.103000	0.267000	10.087000	0.143000	5.743000	14.051000	0.137000	0.021000	0.000148	0.001766	0.167000	vegetable	greenhouse	aerial
cucumber	tulcea	18.710000	0.008000	0.183000	0.051000	0.671000	16.520000	0.071000	23.420000	11.310000	9.952000	0.336000	0.003000	0.268000	0.611000	vegetable	field	aerial
cucumber	tulcea	18.420000	0.008000	0.150000	0.030000	0.910000	6.100000	0.050000	23.310000	13.590000	7.190000	0.720000	0.003000	0.340000	0.540000	vegetable	greenhouse	aerial
cucumber	valcea	22.110000	0.010000	0.123000	0.051000	1.001000	11.470000	0.031000	20.123000	12.780000	6.530000	0.203000	0.010000	0.112000	0.420000	vegetable	field	aerial
cucumber	valcea	11.010000	0.001000	0.720000	0.012000	0.101000	8.320000	0.038000	22.630000	5.890000	4.230000	0.510000	0.110000	0.127000	0.220000	vegetable	greenhouse	aerial
cucumber	vrancea	18.771000	0.010000	0.640000	0.072000	2.310000	4.320000	0.072000	57.320000	13.210000	4.780000	6.230000	0.256000	0.213000	1.210000	vegetable	field	aerial
cucumber	vrancea	17.310000	0.012000	0.823000	0.046000	1.012000	5.920000	1.112000	58.090000	7.230000	1.860000	9.120000	0.721000	0.120000	0.960000	vegetable	greenhouse	aerial
garlic	arges	8.410000	0.001000	0.110000	0.010000	0.580000	12.627000	1.101000	43.114000	13.650000	0.522000	2.255000	0.001000	0.001766	0.315000	vegetable	field	underground
garlic	arges	6.563000	0.005000	0.121000	0.003000	1.208000	10.741000	1.308000	62.612000	5.211000	0.155000	2.002000	0.001000	0.001766	0.454000	vegetable	greenhouse	underground
garlic	bacau	11.560000	0.060000	0.270000	0.020000	1.920000	20.030000	0.990000	38.000000	12.180000	3.080000	1.640000	0.140000	0.001766	0.522000	vegetable	field	underground
garlic	bacau	8.813000	0.030000	0.321000	0.002000	3.132000	13.456000	2.431000	46.812000	1.501000	2.660000	2.220000	0.001000	0.002100	0.449000	vegetable	greenhouse	underground
garlic	bihor	14.520000	0.190000	1.019000	0.001000	0.112000	20.620000	2.032000	22.400000	9.001000	1.209000	0.123000	1.022000	0.107000	0.718000	vegetable	field	underground
garlic	bihor	13.780000	0.760000	1.069000	0.001000	0.312000	20.100000	2.163000	22.600000	8.700000	1.101000	0.361000	1.031000	0.012000	0.657000	vegetable	greenhouse	underground
garlic	braila	7.890000	0.001000	0.112000	0.067000	1.890000	9.320000	1.032000	40.120000	21.121000	1.102000	2.012000	0.007000	0.001000	0.612000	vegetable	field	underground

garlic	braila	3.298000	0.001000	0.014000	0.021000	1.600000	9.760000	1.001000	38.017000	16.320000	1.066000	2.078000	0.001000	0.001000	0.741000	vegetable	greenhouse	underground
garlic	buzau	12.810000	0.214000	0.483000	0.021000	1.062000	25.382000	0.893000	39.904000	18.020000	6.186000	2.134000	1.060000	0.001000	0.317000	vegetable	field	underground
garlic	buzau	8.081000	0.052000	0.622000	0.025000	2.106000	16.190000	2.217000	48.663000	2.803000	4.906000	2.825000	0.121000	0.001766	0.221000	vegetable	greenhouse	underground
garlic	calarasi	31.422000	0.001000	0.026000	0.005000	2.528000	30.421000	1.823000	47.170000	18.211000	4.237000	2.123000	0.001000	0.001000	0.714000	vegetable	field	underground
garlic	calarasi	29.532000	0.001000	0.121000	0.012000	3.158000	14.961000	4.216000	97.274000	11.121000	5.182000	6.716000	0.001000	0.001000	1.072000	vegetable	greenhouse	underground
garlic	constanta	6.217000	0.078000	0.011000	0.009000	1.672000	16.200000	1.322000	38.160000	7.570000	1.024000	1.712000	0.012000	0.001000	0.807000	vegetable	field	underground
garlic	constanta	4.212000	0.097000	0.098000	0.008000	1.918000	15.001000	1.523000	40.022000	2.314000	1.033000	2.130000	0.031000	0.001000	0.633000	vegetable	greenhouse	underground
garlic	dambovita	12.112000	0.001000	0.100000	0.004000	0.980000	29.412000	1.317000	49.283000	13.222000	58.231000	2.826000	0.028000	0.001000	0.911000	vegetable	field	underground
garlic	dambovita	11.511000	0.001000	0.122000	0.003000	1.958000	18.041000	2.021000	47.206000	5.342000	5.112000	3.773000	0.048000	0.001000	0.793000	vegetable	greenhouse	underground
garlic	dolj	29.147000	0.023000	7.141000	0.647000	2.121000	21.330000	1.006000	60.221000	14.145000	4.004000	16.037000	0.001000	0.001766	199.222000	vegetable	field	underground
garlic	dolj	28.364000	0.067000	9.035000	0.354000	1.057000	23.047000	0.941000	74.101000	17.007000	2.871000	11.222000	0.001900	0.014000	216.004000	vegetable	greenhouse	underground
garlic	galati	15.620000	0.001000	2.330000	0.072000	2.960000	10.127000	1.802000	41.320000	9.312000	1.789000	5.012000	0.302000	0.001000	0.980000	vegetable	field	underground
garlic	galati	14.312000	0.001000	0.320000	0.064000	3.221000	9.780000	2.317000	41.789000	9.001000	1.021000	5.667000	0.078000	0.001000	0.780000	vegetable	greenhouse	underground
garlic	giurgiu	11.210000	0.051000	0.110000	0.050000	1.124000	26.614000	1.318000	49.112000	13.917000	3.272000	3.171000	0.008000	0.002000	0.421000	vegetable	field	underground
garlic	giurgiu	9.227000	0.140000	0.304000	0.001000	2.080000	16.536000	1.439000	52.113000	2.207000	2.291000	4.816000	0.001000	0.001000	0.612000	vegetable	greenhouse	underground
garlic	ialomita	22.124000	0.003400	9.025000	0.214000	2.204000	33.017000	4.014000	101.520000	20.104000	3.007000	6.224000	0.374000	0.005700	1.214000	vegetable	field	underground
garlic	ialomita	18.036000	0.003000	7.341000	0.367000	3.041000	25.141000	1.578000	92.547000	16.009000	5.436000	6.587000	0.401000	0.003400	2.036000	vegetable	greenhouse	underground
garlic	ilfov	12.350000	0.050000	0.510000	0.020000	1.250000	24.390000	1.280000	47.150000	15.490000	2.990000	3.890000	0.080000	0.001766	0.324000	vegetable	field	underground
garlic	ilfov	10.128000	0.040000	0.525000	0.001000	3.450000	16.313000	1.919000	57.520000	3.210000	2.330000	5.327000	0.001000	0.001766	0.531000	vegetable	greenhouse	underground
garlic	mehedinti	2.780000	0.021000	0.078000	0.006000	1.120000	12.920000	0.918000	22.780000	6.360000	1.002000	3.780000	0.210000	0.001000	0.890000	vegetable	field	underground
garlic	mehedinti	8.120000	0.100000	0.160000	0.007000	0.207000	11.650000	0.978000	23.160000	7.870000	1.021000	3.960000	0.036000	0.001000	0.900000	vegetable	greenhouse	underground
garlic	olt	32.111000	0.000148	5.004000	0.003000	6.101000	33.057000	3.207000	62.058000	21.004000	3.661000	12.121000	0.001766	0.003000	88.147000	vegetable	field	underground
garlic	olt	27.031000	0.000320	5.544000	0.001000	5.883000	30.007000	2.883000	57.111000	20.011000	2.009000	9.008000	0.001766	0.002000	86.559000	vegetable	greenhouse	underground
garlic	tulcea	15.175000	0.001000	0.109000	0.065000	1.021000	15.144000	2.348000	50.103000	15.430000	1.489000	3.609000	0.011000	0.001000	0.695000	vegetable	field	underground
garlic	tulcea	14.271000	0.001000	0.360000	0.004000	2.478000	7.839000	2.739000	59.420000	4.411000	2.992000	5.280000	0.003000	0.001000	0.711000	vegetable	greenhouse	underground
garlic	valcea	5.213000	0.050000	0.079000	0.032000	0.178000	12.921000	1.023000	32.820000	23.502000	0.182000	2.492000	0.001000	0.001000	0.129000	vegetable	field	underground
garlic	valcea	3.778000	0.050000	0.360000	0.002000	1.161000	10.719000	1.543000	57.400000	6.941000	0.081000	1.917000	0.001000	0.001000	0.237000	vegetable	greenhouse	underground
garlic	vrancea	10.860000	0.001000	0.542000	0.050000	1.729000	17.630000	1.240000	44.507000	17.540000	2.427000	2.342000	0.110000	0.001766	0.675000	vegetable	field	underground
garlic	vrancea	9.320000	0.001000	0.611000	0.003000	2.129000	14.232000	3.112000	62.122000	3.916000	1.962000	2.935000	0.052000	0.001000	0.329000	vegetable	greenhouse	underground
lettuce	arges	12.169000	0.001000	0.698000	0.129000	4.360000	43.127000	9.287000	301.002000	43.123000	4.560000	17.810000	1.120000	0.001000	0.276000	vegetable	field	aerial
lettuce	arges	40.630000	0.280000	1.060000	0.180000	4.920000	49.670000	8.270000	293.900000	71.250000	8.930000	16.900000	0.260000	0.001766	0.211000	vegetable	greenhouse	aerial
lettuce	bacau	19.062000	0.001000	3.220000	0.113000	0.012000	89.232000	6.912000	196.212000	111.214000	9.326000	2.389000	0.001000	0.001000	0.111000	vegetable	field	aerial
lettuce	bacau	20.140000	1.360000	2.240000	0.250000	2.250000	55.930000	8.070000	172.800000	122.100000	8.240000	5.690000	0.002791	0.001766	0.115000	vegetable	greenhouse	aerial
lettuce	bihor	14.103000	0.312000	1.230000	0.001000	0.023000	19.689000	2.361000	61.023000	102.360000	4.630000	0.120000	1.233000	0.102000	0.303000	vegetable	field	aerial
lettuce	bihor	14.022000	1.201000	1.364000	0.001000	0.056000	18.362000	2.698000	61.320000	98.780000	4.001000	0.162000	1.262000	0.012000	0.259000	vegetable	greenhouse	aerial
lettuce	braila	8.270000	0.001000	0.432000	0.006000	1.321000	9.360000	1.021000	100.100000	121.012000	1.892000	1.980000	0.001000	0.001000	0.156000	vegetable	field	aerial
lettuce	braila	6.360000	0.001000	0.126000	0.002900	1.023000	9.862000	1.004000	96.301000	100.200000	1.637000	2.113000	0.001000	0.001000	0.214000	vegetable	greenhouse	aerial
lettuce	bucuresti	27.925000	0.027000	1.578000	0.402000	5.698000	102.130000	15.989000	600.113000	130.114000	7.891000	20.113000	0.115000	0.011000	0.201000	vegetable	field	aerial
lettuce	bucuresti	27.040000	0.013000	1.410000	0.390000	5.470000	132.900000	15.040000	598.200000	126.800000	7.250000	21.500000	0.050000	0.001766	0.236000	vegetable	greenhouse	aerial
lettuce	buzau	21.220000	0.217000	1.327000	0.207000	7.214000	92.201000	7.112000	523.120000	86.170000	9.348000	28.412000	0.201000	0.052000	0.119000	vegetable	field	aerial
lettuce	buzau	22.023000	0.001000	1.110000	0.198000	5.631000	88.712000	9.130000	549.780000	84.312000	9.107000	26.712000	0.098000	0.002000	0.182000	vegetable	greenhouse	aerial
lettuce	calarasi	46.120000	0.010000	0.420000	0.001000	1.120000	60.300000	1.121000	98.710000	75.670000	34.120000	2.360000	0.012000	0.001000	0.036000	vegetable	field	aerial
lettuce	calarasi	85.250000	0.160000	0.840000	0.050000	0.900000	63.590000	4.630000	141.900000	155.500000	69.310000	5.610000	0.140000	0.260000	0.121000	vegetable	greenhouse	aerial
lettuce	constanta	7.560000	0.011000	0.320000	0.003000	1.220000	16.860000	1.756000	78.660000	8.310000	1.756000	0.890000	0.003000	0.001000	0.602000	vegetable	field	aerial
lettuce	constanta	6.390000	0.032000	0.512000	0.002000	1.364000	8.390000	1.498000	79.840000	10.012000	1.732000	0.989000	0.005000	0.001000	0.587000	vegetable	greenhouse	aerial
lettuce	dambovita	66.310000	0.001000	0.560000	0.002000	1.200000	61.380000	1.023000	99.100000	14.316000	58.140000	2.630000	0.003000	0.001000	0.210000	vegetable	field	aerial
lettuce	dambovita	58.190000	0.011000	0.600000	0.100000	1.239000	49.620000	2.360000	98.210000	15.260000	15.712000	2.612000	0.210000	0.121000	0.134000	vegetable	greenhouse	aerial

lettuce	dolj	33.041000	0.024000	0.067000	0.147000	2.331000	21.244000	0.257000	212.113000	88.254000	2.121000	4.025000	0.044000	0.001000	1.027000	vegetable	field	aerial
lettuce	dolj	26.235000	0.036000	0.037000	0.005000	1.058000	23.007000	1.009000	188.140000	80.111000	2.357000	3.533000	0.042000	0.001000	0.987000	vegetable	greenhouse	aerial
lettuce	galati	15.230000	0.001000	2.033000	0.007000	1.369000	11.630000	1.562000	100.230000	101.200000	3.660000	3.456000	0.113000	0.001000	0.314000	vegetable	field	aerial
lettuce	galati	12.530000	0.001000	0.562000	0.005000	1.622000	10.298000	1.635000	95.480000	98.560000	2.001000	3.621000	0.210000	0.001000	0.301000	vegetable	greenhouse	aerial
lettuce	giurgiu	37.121000	0.001000	1.003000	2.013000	6.250000	56.610000	10.120000	260.130000	76.400000	10.310000	7.216000	0.127000	0.002000	0.298000	vegetable	field	aerial
lettuce	giurgiu	36.120000	0.480000	0.850000	0.140000	5.300000	51.420000	9.350000	251.800000	71.890000	8.970000	5.440000	0.002791	0.001766	0.252000	vegetable	greenhouse	aerial
lettuce	ialomita	12.417000	0.002700	3.010000	1.025000	6.444000	39.417000	0.367000	218.190000	90.147000	8.111000	1.121000	0.647000	0.004300	5.036000	vegetable	field	aerial
lettuce	ialomita	10.022000	0.003800	3.411000	0.358000	12.367000	44.084000	0.414000	204.258000	88.014000	5.036000	1.364000	0.444000	0.005900	4.746000	vegetable	greenhouse	aerial
lettuce	ilfov	40.021000	0.001000	19.210000	0.217000	4.691000	87.156000	6.981000	499.100000	98.789000	12.133000	18.170000	0.301000	0.001000	0.211000	vegetable	field	aerial
lettuce	ilfov	38.112000	0.001000	2.314000	0.200000	3.721000	88.612000	8.239000	468.170000	110.120000	11.012000	17.120000	0.128000	0.004000	0.239000	vegetable	greenhouse	aerial
lettuce	mehedinti	5.360000	0.010000	0.116000	0.001000	1.012000	11.265000	0.692000	56.630000	75.630000	2.360000	2.648000	0.120000	0.001000	0.215000	vegetable	field	aerial
lettuce	mehedinti	8.690000	0.026000	0.238000	0.001000	1.017000	11.001000	0.978000	57.398000	79.456000	3.102000	3.261000	0.021000	0.001000	0.226000	vegetable	greenhouse	aerial
lettuce	olt	17.222000	0.147000	1.009000	0.645000	2.128000	26.340000	0.358000	317.309000	111.205000	2.137000	11.141000	0.114000	0.004100	4.141000	vegetable	field	aerial
lettuce	olt	15.661000	0.555000	1.166000	0.444000	2.308000	26.141000	0.404000	333.071000	107.351000	1.041000	9.547000	0.167000	0.006700	3.001000	vegetable	greenhouse	aerial
lettuce	tulcea	15.175000	0.001000	0.109000	0.065000	1.021000	15.144000	2.348000	50.103000	15.430000	1.489000	3.609000	0.011000	0.001000	0.695000	vegetable	field	aerial
lettuce	tulcea	14.271000	0.001000	0.360000	0.004000	2.478000	7.839000	2.739000	59.420000	4.411000	2.992000	5.280000	0.003000	0.001000	0.711000	vegetable	greenhouse	aerial
lettuce	valcea	36.120000	0.102000	1.112000	1.620000	12.110000	32.100000	9.200000	897.000000	112.200000	10.570000	29.120000	0.120000	0.001000	1.120000	vegetable	field	aerial
lettuce	valcea	47.260000	0.190000	2.040000	1.060000	13.820000	28.040000	8.900000	1575.000000	104.100000	11.470000	40.320000	0.260000	0.090000	0.132000	vegetable	greenhouse	aerial
lettuce	vrancea	18.321000	0.001000	2.345000	0.416000	5.612000	38.612000	8.311000	532.140000	112.321000	4.560000	22.114000	0.312000	0.001000	0.222000	vegetable	field	aerial
lettuce	vrancea	16.521000	0.001000	0.521000	0.298000	5.911000	36.120000	10.128000	556.101000	110.421000	3.201000	28.312000	0.089000	0.001000	0.262000	vegetable	greenhouse	aerial
onion	arges	8.200000	0.050000	0.190000	0.030000	1.580000	15.670000	1.210000	34.140000	16.650000	2.520000	1.500000	0.130000	0.001766	0.375000	vegetable	field	underground
onion	arges	12.530000	0.050000	0.680000	0.170000	2.280000	47.740000	3.080000	187.600000	24.210000	4.150000	3.020000	0.120000	0.001766	0.254000	vegetable	greenhouse	underground
onion	bacau	16.840000	0.090000	0.100000	0.009000	0.800000	2.500000	0.030000	12.200000	7.710000	4.650000	1.300000	0.003000	0.330000	0.110000	vegetable	field	underground
onion	bacau	10.910000	0.050000	0.270000	0.020000	1.320000	17.520000	1.430000	49.810000	13.510000	3.660000	2.170000	0.070000	0.001766	0.119000	vegetable	greenhouse	underground
onion	bihor	9.320000	0.121000	1.506000	0.002000	0.018000	4.422000	2.523000	5.717000	16.211000	2.723000	0.133000	0.002000	0.011000	0.102000	vegetable	field	underground
onion	bihor	11.360000	0.336000	1.721000	0.003000	1.009000	3.962000	4.216000	6.271000	17.222000	2.412000	0.417000	0.004000	0.002000	0.089000	vegetable	greenhouse	underground
onion	braila	21.220000	0.001000	0.110000	0.008000	1.360000	8.160000	0.011000	10.310000	16.170000	2.110000	1.001000	0.001000	0.010000	0.312000	vegetable	field	underground
onion	braila	16.130000	0.001000	0.012000	0.003000	1.420000	22.420000	0.003000	15.610000	14.360000	2.012000	1.210000	0.003000	0.021000	0.361000	vegetable	greenhouse	underground
onion	bucuresti	15.370000	0.080000	0.120000	0.004000	0.382000	3.250000	0.032000	14.790000	10.270000	3.129000	2.691000	0.004000	0.261000	0.200000	vegetable	field	underground
onion	bucuresti	12.120000	0.060000	0.270000	0.060000	1.770000	18.760000	1.480000	52.890000	19.720000	3.640000	4.980000	0.070000	0.001766	0.215000	vegetable	greenhouse	underground
onion	buzau	7.860000	0.040000	0.430000	0.020000	1.060000	15.820000	1.330000	44.040000	18.020000	2.860000	3.340000	0.060000	0.001766	0.417000	vegetable	field	underground
onion	buzau	10.800000	0.050000	0.220000	0.050000	2.160000	14.100000	1.270000	41.630000	15.030000	2.960000	1.250000	0.120000	0.001766	0.321000	vegetable	greenhouse	underground
onion	calarasi	39.420000	0.012100	0.526000	0.007000	1.280000	22.420000	0.123000	9.170000	11.210000	6.230000	1.230000	0.001000	0.012300	0.402000	vegetable	field	underground
onion	calarasi	42.520000	0.036000	0.721000	0.032000	1.580000	20.961000	1.260000	10.271000	17.210000	5.112000	2.730000	0.002000	0.072000	0.721000	vegetable	greenhouse	underground
onion	constanta	19.960000	0.011000	0.079000	0.011000	1.290000	2.612000	0.012000	12.310000	14.130000	1.113000	0.012000	0.006000	0.010000	0.031200	vegetable	field	underground
onion	constanta	18.160000	0.021000	0.120000	0.112000	1.611000	2.581000	0.009000	38.612000	15.207000	2.311000	0.066000	0.007000	0.011000	0.045000	vegetable	greenhouse	underground
onion	dambovita	69.112000	0.013000	0.428000	0.005000	1.280000	23.420000	0.117000	9.283000	8.212000	23.231000	1.260000	0.001000	0.011000	0.411000	vegetable	field	underground
onion	dambovita	73.510000	0.039000	0.622000	0.012000	1.580000	21.941000	1.121000	8.276000	9.382000	5.110000	1.730000	0.002000	0.061000	0.713000	vegetable	greenhouse	underground
onion	dolj	4.324000	0.054000	0.065000	0.004000	0.141000	12.124000	1.201000	79.325000	0.147000	0.004000	0.011000	0.030000	0.003400	1.001000	vegetable	field	underground
onion	dolj	5.547000	0.032500	0.130000	0.002000	0.010000	14.202000	0.111000	77.547000	0.034000	0.003000	0.012000	0.024000	0.002400	0.871000	vegetable	greenhouse	underground
onion	galati	11.211000	0.032000	0.214000	0.041000	1.679000	16.613000	1.440000	48.517000	17.214000	3.207000	2.032000	0.101000	0.001000	0.125000	vegetable	field	underground
onion	galati	12.003000	0.039000	0.100000	0.060000	1.729000	18.100000	2.120000	61.212000	18.160000	3.412000	2.305000	0.156000	0.001000	0.119000	vegetable	greenhouse	underground
onion	giurgiu	11.200000	0.050000	0.410000	0.050000	1.540000	23.640000	1.680000	76.100000	13.970000	3.270000	4.710000	0.080000	0.001766	0.411000	vegetable	field	underground
onion	giurgiu	21.270000	0.040000	0.440000	0.210000	4.080000	21.560000	1.430000	113.000000	15.070000	17.210000	23.160000	0.150000	0.001766	0.326000	vegetable	greenhouse	underground
onion	ialomita	2.004000	0.024700	0.143100	1.009000	2.367000	36.581000	5.471000	98.212400	36.357000	11.311000	0.114000	0.005700	0.001700	47.390000	vegetable	field	underground
onion	ialomita	3.141000	0.010500	0.106000	0.873000	4.579000	41.374000	6.030000	97.047000	38.541000	10.555000	1.009000	0.141000	0.001600	40.414000	vegetable	greenhouse	underground
onion	ilfov	14.440000	0.040000	0.470000	0.030000	2.070000	14.090000	1.120000	41.960000	12.800000	3.060000	4.310000	0.090000	0.001766	0.312000	vegetable	field	underground

onion	ilfov	10.280000	0.040000	0.500000	0.090000	4.450000	32.330000	1.990000	112.500000	14.000000	3.330000	18.370000	0.090000	0.001766	0.530000	vegetable	greenhouse	underground
onion	mehedinti	12.310000	0.010000	0.021000	0.001000	1.001000	2.560000	1.039000	6.170000	13.222000	2.033000	0.291000	0.011000	0.001000	0.410000	vegetable	field	underground
onion	mehedinti	19.620000	0.032000	1.231000	0.033000	1.239000	2.132000	2.301000	12.003000	16.126000	2.160000	0.311000	0.006000	0.001000	0.312000	vegetable	greenhouse	underground
onion	olt	62.154000	0.000148	0.103000	0.004000	1.004000	14.235000	1.257000	12.050000	3.257000	0.147000	1.001000	0.025000	0.002700	3.541000	vegetable	field	underground
onion	olt	68.327000	0.000151	0.141000	0.002000	0.087000	12.036000	2.367000	14.370000	4.001000	0.678000	1.367000	0.041000	0.001600	5.036700	vegetable	greenhouse	underground
onion	tulcea	16.750000	0.081000	0.179000	0.009000	0.210000	2.344000	0.023000	8.596000	7.430000	3.487000	0.209000	0.003000	0.267000	0.395000	vegetable	field	underground
onion	tulcea	17.710000	0.090000	1.060000	0.006000	0.470000	2.390000	0.020000	9.420000	7.410000	2.990000	0.280000	0.003000	0.340000	0.110000	vegetable	greenhouse	underground
onion	valcea	15.213000	0.050000	0.379000	0.052000	1.780000	8.921000	1.021000	52.820000	23.520000	6.820000	2.892000	0.182000	0.026200	0.128000	vegetable	field	underground
onion	valcea	17.780000	0.050000	0.560000	0.140000	2.610000	20.790000	2.430000	137.400000	46.940000	7.810000	6.170000	0.120000	0.080000	0.157000	vegetable	greenhouse	underground
onion	vrancea	10.100000	0.030000	0.240000	0.040000	1.790000	17.630000	1.440000	44.570000	17.240000	3.470000	1.320000	0.100000	0.001766	0.175000	vegetable	field	underground
onion	vrancea	12.300000	0.038000	0.110000	0.062000	1.829000	18.232000	2.120000	63.220000	18.960000	3.620000	2.350000	0.152000	0.001000	0.129000	vegetable	greenhouse	underground
zucchini	arges	21.021000	0.009000	0.225000	0.315000	7.303000	9.214000	0.087000	26.321000	13.057000	9.014000	0.414000	0.008000	0.358000	0.808000	vegetable	field	aerial
zucchini	arges	17.020000	0.004200	0.147000	0.260000	5.670000	7.040000	0.030000	25.510000	11.820000	7.070000	0.350000	0.003000	0.260000	0.590000	vegetable	greenhouse	aerial
zucchini	bacau	22.036000	0.021000	0.075000	0.099000	4.125000	9.258000	0.060000	29.328000	16.025000	6.258000	5.047000	0.008000	0.119700	0.388000	vegetable	field	aerial
zucchini	bacau	18.970000	0.004900	0.050000	0.080000	2.490000	7.960000	0.030000	25.990000	12.740000	4.750000	4.560000	0.003000	0.160000	0.210000	vegetable	greenhouse	aerial
zucchini	bihor	19.107000	0.003000	0.749000	0.097000	0.055000	11.337000	0.081000	8.041000	16.014000	10.527000	0.008000	3.121000	0.357000	0.509000	vegetable	field	aerial
zucchini	bihor	17.210000	0.002000	0.620000	0.023000	0.021000	10.140000	0.078000	7.260000	15.260000	9.111000	0.001000	2.360000	0.121000	0.465000	vegetable	greenhouse	aerial
zucchini	braila	9.556000	0.009000	0.589000	0.704000	1.674000	6.058000	0.047000	15.009000	12.414000	9.006000	0.376000	0.005000	0.002000	0.314000	vegetable	field	aerial
zucchini	braila	9.210000	0.001000	0.512000	0.410000	1.560000	5.420000	0.007000	14.560000	11.210000	8.690000	0.302000	0.001000	0.001000	0.126000	vegetable	greenhouse	aerial
zucchini	bucuresti	26.020000	0.009000	0.022000	0.307000	2.064000	12.144000	0.236000	23.010000	14.111000	4.308000	0.378000	0.009000	0.355000	31.241000	vegetable	field	aerial
zucchini	bucuresti	22.130000	0.007000	0.015000	0.201000	1.512000	8.230000	0.021000	18.010000	13.114000	2.530000	0.243000	0.001000	0.296000	28.130000	vegetable	greenhouse	aerial
zucchini	buzau	18.067000	0.007000	0.205000	0.099000	0.966000	15.222000	0.054000	17.222000	19.244000	6.202000	0.641000	0.009000	0.347000	0.101000	vegetable	field	aerial
zucchini	buzau	16.560000	0.002000	0.162000	0.073000	0.865000	13.520000	0.017000	16.670000	15.040000	4.725000	0.168000	0.003000	0.272000	0.087000	vegetable	greenhouse	aerial
zucchini	calarasi	18.111000	0.007000	0.213000	0.401000	4.101000	11.324000	0.036000	24.101000	13.047000	9.204000	0.519000	0.007000	0.201000	0.741000	vegetable	field	aerial
zucchini	calarasi	17.120000	0.003000	0.156000	0.372000	3.210000	10.260000	0.013000	23.450000	12.620000	8.280000	0.360000	0.002000	0.168000	0.626000	vegetable	greenhouse	aerial
zucchini	constanta	9.541000	0.004000	0.069000	0.327000	2.007000	8.547000	0.036000	14.101000	5.047000	10.247000	0.041000	0.006600	0.001400	0.414000	vegetable	field	aerial
zucchini	constanta	8.230000	0.001000	0.042000	0.200000	1.620000	6.830000	0.012000	12.360000	4.560000	9.211000	0.011000	0.001000	0.001000	0.302000	vegetable	greenhouse	aerial
zucchini	dambovita	19.207000	0.009000	0.206000	0.311000	1.888000	13.047000	0.025000	16.003000	11.404000	17.009000	0.307000	0.047000	0.233000	0.505000	vegetable	field	aerial
zucchini	dambovita	18.960000	0.002000	0.148000	0.203000	1.230000	12.460000	0.011000	14.780000	9.360000	16.280000	0.231000	0.012000	0.109000	0.498000	vegetable	greenhouse	aerial
zucchini	dolj	17.333000	0.005000	2.369000	0.687000	0.777000	7.009000	0.089000	8.002000	9.136000	4.225000	0.505000	0.066000	0.001766	0.333000	vegetable	field	aerial
zucchini	dolj	15.057000	0.001000	1.759000	0.355000	0.612000	6.557000	0.073000	7.635000	9.004000	2.087000	0.413000	0.041000	0.001766	0.188000	vegetable	greenhouse	aerial
zucchini	galati	19.888000	0.006000	1.852000	0.518000	3.007000	5.881000	0.074000	15.369000	9.007000	10.525000	0.903000	0.063000	0.001100	0.808000	vegetable	field	aerial
zucchini	galati	18.021000	0.001000	1.012000	0.451000	2.630000	5.210000	0.021000	14.560000	7.220000	9.231000	0.678000	0.031000	0.001000	0.512000	vegetable	greenhouse	aerial
zucchini	giurgiu	31.025000	0.009000	0.178000	0.202000	2.888000	16.805000	0.046000	20.141000	15.697000	8.014000	0.325000	0.007000	0.405000	0.301000	vegetable	field	aerial
zucchini	giurgiu	27.100000	0.006000	0.142000	0.135000	2.091000	15.820000	0.012000	19.580000	15.470000	7.143000	0.210000	0.003000	0.326000	0.267000	vegetable	greenhouse	aerial
zucchini	ialomita	11.005000	0.000148	3.331000	1.111000	15.054000	13.057000	1.001000	7.136000	10.266000	14.142000	0.505000	0.255000	0.084000	0.209000	vegetable	field	aerial
zucchini	ialomita	9.367000	0.000148	2.058000	0.978000	13.121000	13.001000	0.988000	6.773000	8.025000	12.036000	0.453000	0.157000	0.043000	0.163000	vegetable	greenhouse	aerial
zucchini	ilfov	22.010000	0.009000	0.444000	0.647000	2.008000	15.999000	0.047000	19.205000	15.414000	7.014000	0.647000	0.008000	0.414000	29.008000	vegetable	field	aerial
zucchini	ilfov	20.330000	0.005000	0.151000	0.104000	1.459000	14.140000	0.019000	18.110000	13.780000	5.128000	0.201000	0.003000	0.217000	27.100000	vegetable	greenhouse	aerial
zucchini	mehedinti	7.323000	0.007000	0.085000	0.076000	1.947000	7.084000	0.008000	8.547000	5.414000	8.327000	0.987000	0.032000	0.002100	0.307000	vegetable	field	aerial
zucchini	mehedinti	6.350000	0.001000	0.057000	0.045000	1.001000	6.590000	0.005000	7.890000	4.680000	7.211000	0.528000	0.020000	0.001000	0.162000	vegetable	greenhouse	aerial
zucchini	olt	10.033000	0.002800	1.155000	0.101000	2.001000	4.012000	0.044000	6.666000	0.677000	0.360000	0.107000	0.001766	0.001766	0.109000	vegetable	field	aerial
zucchini	olt	9.125000	0.000148	0.625000	0.009700	1.366000	3.111000	0.310000	5.037000	0.499000	0.211000	0.086000	0.001766	0.001766	0.097000	vegetable	greenhouse	aerial
zucchini	tulcea	14.950000	0.001300	0.160000	0.070000	2.380000	6.390000	0.020000	19.000000	11.480000	3.940000	0.740000	0.003000	0.330000	0.090000	vegetable	field	aerial
zucchini	tulcea	18.710000	0.008000	0.183000	0.051000	0.671000	16.520000	0.071000	23.420000	11.310000	9.952000	0.336000	0.003000	0.268000	0.611000	vegetable	greenhouse	aerial
zucchini	valcea	12.025000	0.003000	0.102000	0.189000	2.005000	13.222000	0.067000	21.222000	15.007000	6.121000	0.315000	0.054000	0.212000	0.608000	vegetable	field	aerial
zucchini	valcea	10.101000	0.001000	0.223000	0.115000	1.100000	12.410000	0.022000	19.823000	13.111000	5.950000	0.243000	0.011000	0.114000	0.472000	vegetable	greenhouse	aerial

zucchini	vrancea	20.331000	0.017000	0.749000	0.097000	4.101000	6.369000	0.099000	77.212000	17.414000	3.666000	7.002000	0.399000	0.035700	2.333000	vegetable	field	aerial
zucchini	vrancea	19.160000	0.011000	0.689000	0.081000	3.131000	5.130000	0.081000	62.320000	15.210000	3.178000	6.165000	0.278000	0.023000	1.421000	vegetable	greenhouse	aerial

**Mean values**		18.71	0.07	0.87	0.16	2.41	19.73	1.66	86.84	27.26	7.11	3.78	0.16	0.07	4.78
**SE**		0.96	0.01	0.14	0.02	0.22	1.43	0.20	12.41	2.48	0.87	0.47	0.03	0.01	1.73
